# Experimental and Theoretical DFT Investigations in the [2,3]-Wittig-Type Rearrangement of Propargyl/Allyl-Oxy-Pyrazolones

**DOI:** 10.3390/molecules26216557

**Published:** 2021-10-29

**Authors:** Lucia De Crescentini, Gianfranco Favi, Giacomo Mari, Gianluca Ciancaleoni, Marcello Costamagna, Stefania Santeusanio, Fabio Mantellini

**Affiliations:** 1 Department of Biomolecular Sciences, Section of Organic Chemistry and Organic Natural Compounds, University of Urbino “Carlo Bo”, Via I Maggetti 24, 61029 Urbino, PU, Italy; gianfranco.favi@uniurb.it (G.F.); giacomo.mari@uniurb.it (G.M.); stefania.santeusanio@uniurb.it (S.S.); 2 Department of Chemistry and Industrial Chemistry, University of Pisa, Via G. Moruzzi 13, 56124 Pisa, PI, Italy; gianluca.ciancaleoni@unipi.it (G.C.); m.costamagna@studenti.unipi.it (M.C.)

**Keywords:** [2,3]-Wittig rearrangement, pyrazolones, propargyl alcohol, allyl alcohol, 1,2-diaza-,1,3-dienes, DFT study

## Abstract

Here we report the synthesis of interesting 3-alkyl-4-hydroxy-1-aryl-4-(propa-1,2-dienyl)1*H*-pyrazol-5(4*H*)-ones and 9-alkyl-7-aryl-1-oxa-7,8-diazaspiro[4.4]nona-3,8-dien-6-ones, starting from 1,2-diaza-1,3-dienes (DDs) and propargyl alcohol. The reaction proceeds through a sequence Michael-type nucleophilic attack/cyclization/[2,3]-Wittig rearrangement. In the same way, the reaction between the aforementioned DDs and allyl alcohol furnished 4-allyl-4-hydroxy-3-alkyl-1-aryl-1*H*-pyrazol-5(4*H*)-ones. A DFT study was also carried out, in order to have decisive clarifications about the mechanism.

## 1. Introduction

Heterocycles, in particular those that contain one or more nitrogen atoms, are an extremely important class of compounds widely distributed in nature, as they represent the majority of active molecules employed in biological, pharmacological, and industrial chemistry [1,2,3,4,5].

Among them, pyrazolones play a crucial role as they have a wide range of applications, in particular in the pharmaceutical field [6,7,8]. In fact, they are known to have, along with many others, antimicrobial [9], antitubercular [10], antifungal [7,11,12,13], and antibacterial [7,11,12,13] properties, and also antitumor [7,14], gastric secretion stimulatory [15], anticonvulsant [16], and antimalarial [17] activities. Besides, they are employed as starting materials for the preparation of dyes. [18].

It is for this cause that organic chemists [19,20] as well as our group [21] are actively engaged in the development of new synthetic strategies for their synthesis.

In particular, spirocyclic motifs [19,20,21] containing pyrazolone core are widely represented in many synthetic bioactive and natural products [22,23,24,25,26,27,28,29]. So, several methods for the preparation of such complex three-dimensional structures have been developed starting from simple and readily accessible precursors [22,23,24,25,26,27,28,29].

On the other hand, the α-hydroxyallenes are also a valuable class of derivatives that constitute both versatile synthetic intermediates as well as the core of different compounds of synthetic or natural origin, which manifest biological activities [30,31,32,33,34,35,36].

Among the several approaches that have been employed for their synthesis [37,38,39,40,41,42,43,44,45], the [2,3]-Wittig rearrangement of propargylic ethers represents an efficient way to assemble them [46,47]. The major limitation of this versatile bond reorganization process, which has many other applications in organic synthesis, is mainly correlated to the use of strong bases and very low temperatures together with the laborious, complicated, and expensive procedures required [48,49,50].

**Scheme 1:**
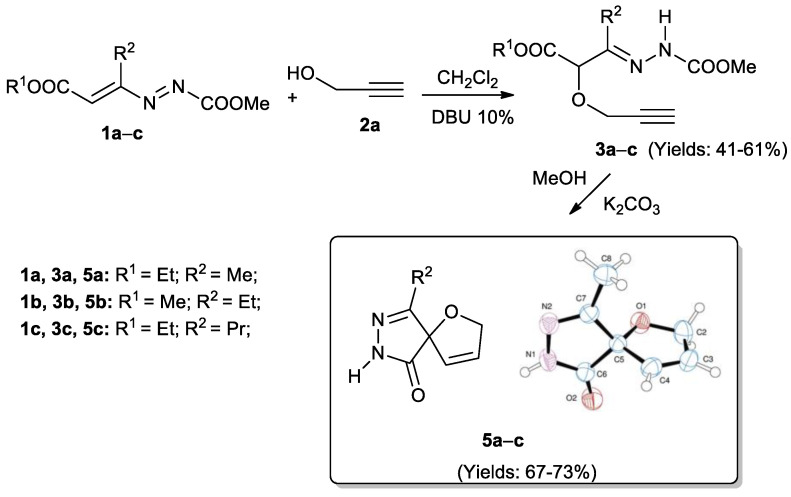
Synthesis of α-(prop-2-yn-1-yloxy)hydrazones **3a**–**c** and of 9-alkyl-1-oxa-7,8-diazaspiro[4.4]nona-3,8-dien-6-ones **5a**–**c** [59].

Quite recently, we have published a preliminary paper, in which, by exploiting the ability of 1,2-diaza-1,3-dienes (DDs) as Michael-acceptors [51,52,53,54,55,56,57,58], we have synthesized α-(prop-2-yn-1-yloxy)hydrazones **3** (Scheme 1) [59]. These compounds containing a propargylic ether function have been demonstrated to be able to give easily and in very mild conditions the [2,3]-Wittig rearrangements. In detail, the synthesis involves 1-methyloxycarbonyl-DDs **1a**–**c** and propargyl alcohol **2a** [60,61] and it is conducted at room temperature, in dichloromethane in the presence of a catalytic amount of a base such as the 1,8-diazabicyclo(5.4.0)undec-7-ene (DBU). In these conditions, the reaction has furnished the corresponding α-(prop-2-yn-1-yloxy)hydrazones **3a**–**c** (41–61%), in 24.0–72.0 h (Scheme 1) [59], by means of the nucleophilic Michael-type attack of the oxygen atom of propargyl alcohol **2a** to the terminal carbon atom of the DDs **1**.

The subsequent treatment of the so-obtained hydrazonic compounds **3a**–**c** with 4 equiv. of a weak base, as K_2_CO_3_, in methanol has furnished new and unexpected 9-alkyl-1-oxa-7,8-diazaspiro[4.4]nona-3,8-dien-6-ones **5a**–**c**, in good yields (67–73%) in 1.0–1.5 h whose structure was confirmed by X-ray spectroscopy (Scheme 1) [59].

For the formation of products **5a**–**c**, we hypothesized a preliminary base-promoted nucleophilic attack of the hydrazonic nitrogen at the ester group of Michael adducts **3**, with the loss of an alcohol molecule, to give the pyrazolone ring, followed by a further cyclization process passing through a [2,3]-Wittig-type rearrangement [59]. In order to better clarify the proposed mechanism and to have some variability on the substituents of DDs as well as to increase the scope of the reaction, we have decided to extend our studies to investigate the reactivity of differently substituted DDs such as 1-aryl-DDs **1d**–**g** with propargyl and allyl alcohols **2a**,**b**, respectively. The results of this study are the object of the present work.

## 2. Results and Discussion

**Scheme 2:**
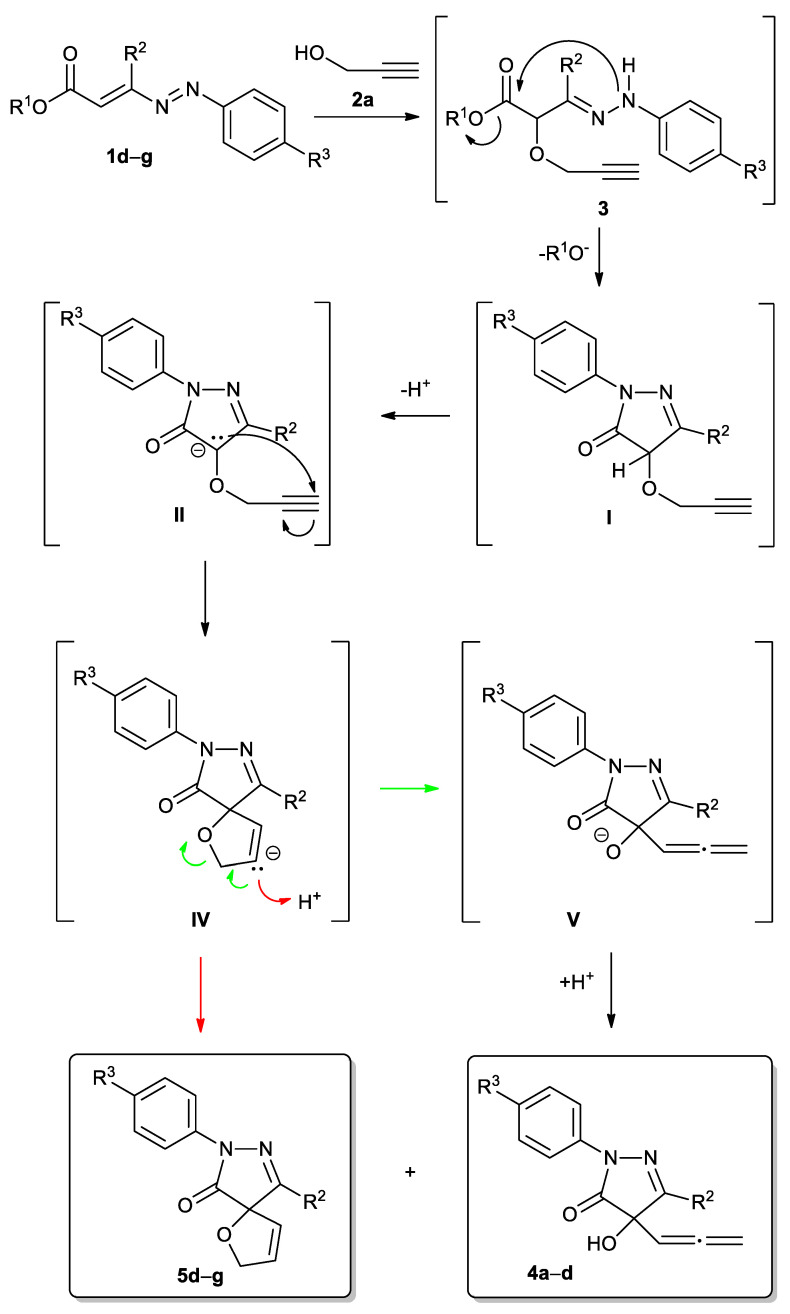
Synthesis and plausible mechanism for the base-promoted synthesis of 3-alkyl-4-hydroxy-1-aryl-4-(propa-1,2-dienyl)1*H*-pyrazol-5(4*H*)-ones **4a**–**d** and of 9-alkyl-7-aryl-1-oxa-7,8-diazaspiro[4.4]nona-3,8-dien-6-ones **5d**–**g**.

To conduct the optimization process, we chose as a representative model the 1-phenyl-DD **1d** and the same propargyl alcohol **2a** (Table 1). Initially, we have verified whether the conditions previously employed with the 1-methyloxycarbonyl-DDs **1a**–**c** and **2a** to obtain the adducts **3**, that is CH_2_Cl_2_, DBU (0.1 equiv.) (Scheme 1) [59], can also be adapted to the DDs otherwise substituted such as **1d** (Table 1, entry 1). Unfortunately, in this case, the reaction did not work at all. This occurrence is probably due to the lower electrophilicity of 1-aryl-DDs **1d**–**g** (Scheme 2) compared to that of 1-methoxycarbonyl-DDs **1a**–**c** (Scheme 1), as evidenced by kinetics studies previously published by some of us [62].

So, we have tested several solvents, such as dichloromethane, acetonitrile, and tetrahydrofuran, and also the same propargyl alcohol used both as solvent and reagent (Table 1). Furthermore, a series of organic and inorganic bases as promoters have been used, such as DBU, DIPEA, NaH, MeONa, and K_2_CO_3_ (Table 1).

The increment of the DBU to 1.0 equivalent in CH_2_Cl_2_, MeCN, THF, or using **2a** as solvent/reagent (entries 2, 5, 7, 8), as well as the use of a catalytic amount of the DBU at 60 °C in MeCN (entry 4), have produced complicated reaction mixtures, in which a TLC analysis revealed the presence of 3-methyl-4-hydroxy-1-phenyl-4-(propa-1,2-dienyl)1*H*-pyrazol-5(4*H*)-one **4a** and 9-methyl-7-phenyl-1-oxa-7,8-diazaspiro[4.4]nona-3,8-dien-6-one **5d** (Table 1), but only in traces. The reactions carried out with DIPEA (entries 9–14) or a catalytic amount of DBU, both at r.t. and by heating (entries 1, 3, 4, and 6) did not work at all, while with NaH (entries 15–20) or MeONa (entries 21–25), they gave complicated mixtures, regardless of the solvent conditions employed.

Additionally, the attempts to carry out the reaction using strong bases at lower temperatures such as NaH at –20 °C (entry 17) or –78 °C (entry 18), as well as MeONa at –20 °C (entry 23) or *t*-BuONa at room temperature (entry 26) or at −20 °C (entry 27) have also failed, giving complicated reaction mixtures.

Additionally in the case of the use of 4 equiv. of K_2_CO_3_ in any solvent at room temperature, only traces of **4a** and **5d** were obtained (entries 23–26).

The trend of the reaction improves by using four equiv. of K_2_CO_3_ using 3 mL of propargy alcohol **2a** as solvent/reagent at 60 °C, giving **4a** and **5d** in 11% and 29% yields, respectively, and these are the best conditions found in our screening (Table 1, entry 32).

At this point, to tentatively improve the yields, we have tried some different carbonates, such as Cs_2_CO_3_ (entry 33) or Na_2_CO_3_ (entry 34), in the same best conditions found with K_2_CO_3_ but we have obtained lower yields of **4a** and **5d**.

So, with these most optimal conditions possible in hand, we performed the reactions between 1-aryl-DDs **1d**–**g** and propargyl alcohol **2a** used as solvent-reagent, at 60 °C in the presence of 4 equiv. of K_2_CO_3_.

After 8.0–11.5 h, at the disappearance of the red color of the DDs, a TLC monitoring revealed a complicated reaction mixture in which there are two main spots, very close to each other. Their separation was very difficult having requested a first purification by column chromatography on silica gel and then by preparative thin-layer chromatography with more successive elutions. The so-obtained products were identified as 3-alkyl-4-hydroxy-1-aryl-4-(propa-1,2-dienyl)1*H*-pyrazol-5(4*H*)-ones **4a**–**d** and 9-alkyl-7-aryl-1-oxa-7,8-diazaspiro[4.4]nona-3,8-dien-6-ones **5d**–**g** (Scheme 2, Table 2). It is impossible to isolate other significant products as the reaction appears as a dirty complicated mixture of degradation compounds.

As the first step of the reaction, we hypothesized the formation of the non-isolable hydrazone intermediates **3**, by means of the nucleophilic Michael-type attack of the oxygen atom of propargyl alcohol **2a** to the terminal carbon atom of the azoene system of the DDs **1**. The base-promoted nucleophilic attack of the hydrazonic nitrogen at the ester group of hydrazones **3**, with the loss of an alcohol molecule, gives the corresponding non-isolable pyrazolone derivative **I** (Scheme 2).

In order to obtain decisive information to explain the subsequent Wittig rearrangement involved in the formation of the following final products **4a**–**d** and **5d**–**g**, a DFT study was conducted.

**Scheme 3:**
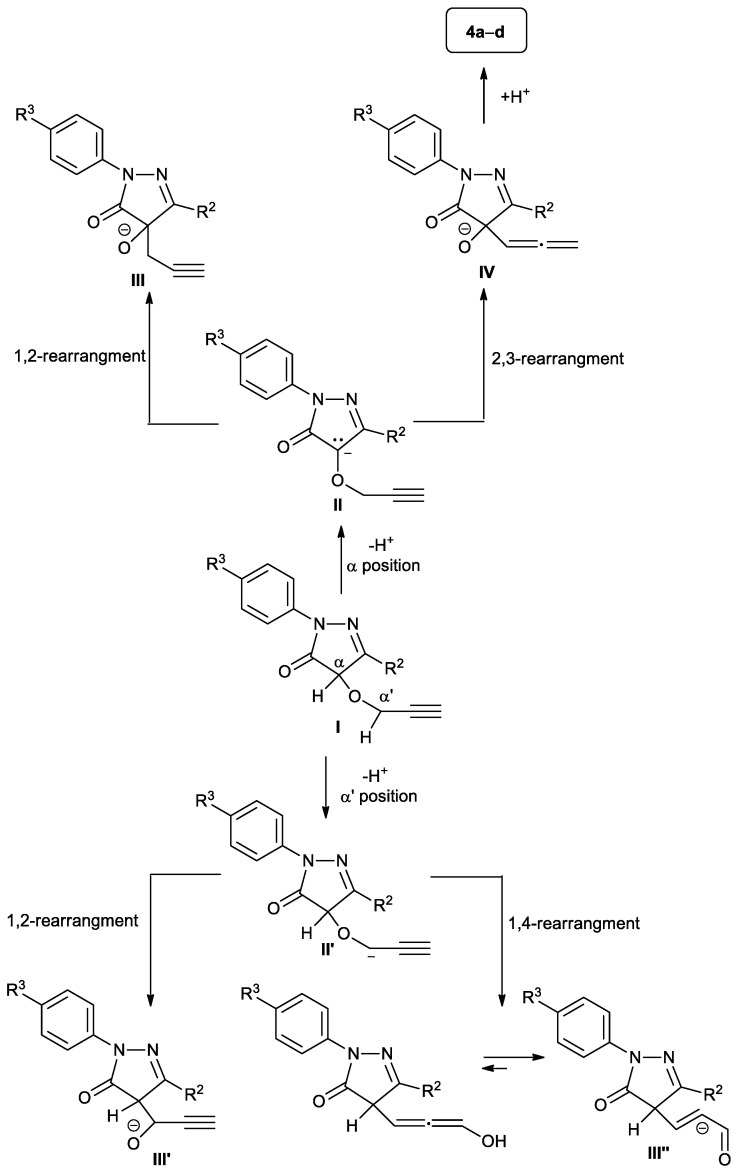
Possible Wittig rearrangements of 4-(prop-2-yn-1-yloxy)-1*H*-pyrazol-5(4*H*)-ones **I**.

The base-promoted deprotonation of the intermediate **I** can occur at the α or α′ positions, leading to the formation of anion species **II** or **II′**, respectively (Scheme 3). Intermediate **II** can theoretically undergo both [1,2]- or [2,3]-Wittig rearrangements, furnishing products **III** or **IV**, respectively, while intermediate **II′** can give the [1,2]- or [1,4]-rearrangement, giving products **III′** or **III″**, respectively (Scheme 3) [40,47,49,63,64,65,66,67,68,69,70,71,72,73,74,75,76,77,78,79,80,81].

It is noteworthy that the process here described is highly regioselective, since the deprotonation occurs only in the α position of the intermediate **I** to produce **II**, as this proton is more acid than the one in the α′ position, being activated both from an amidic carboxylic group as well as from an imino function. As a confirmation of this event, the two products of deprotonation **II** and **II′** have been optimized by DFT methods. As supposed, the former **II** resulted in being more stable than the latter by 33.8 kcal/mol (see Supplementary Materials).

Then, in the formation of the 3-alkyl-4-hydroxy-1-aryl-4-(propa-1,2-dienyl)1*H*-pyrazol-5(4*H*)-ones **4a**–**d**, the Wittig rearrangement observed is of the [2,3]-type, as the spectroscopic data of the products **4** clearly support (Scheme 2 and Scheme 3). To exclude that, in the concomitant formation of the 9-alkyl-7-aryl-1-oxa-7,8-diazaspiro[4.4]nona-3,8-dien-6-ones **5d**–**g**, the [1,2]-Wittig rearrangement was involved by means of cyclization of intermediate **III** (Scheme 3), a DFT study has been carried out.

**Figure 1:**
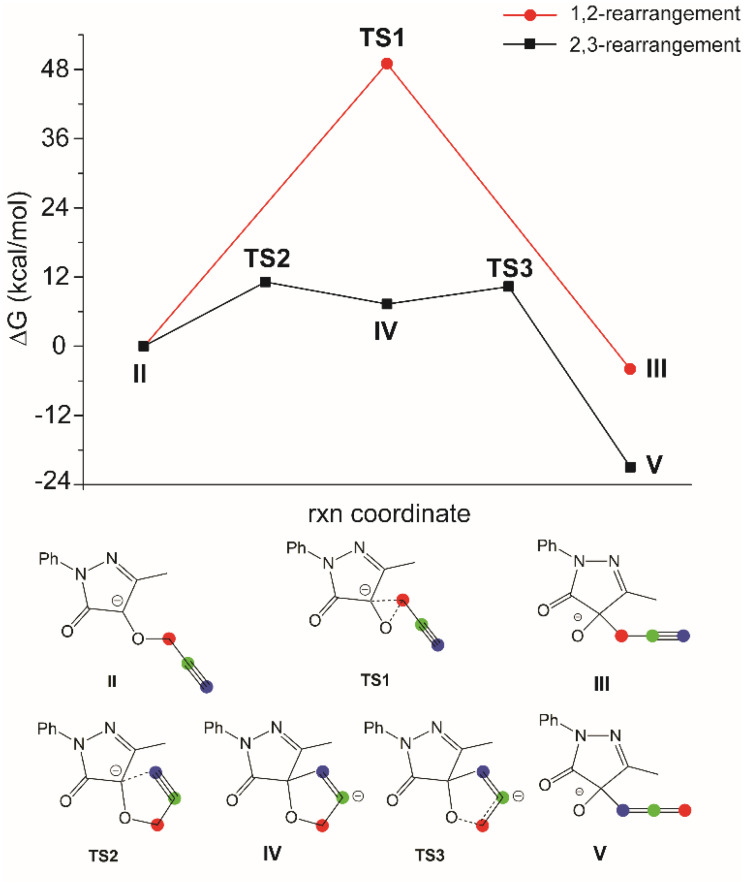
Energy profile for the [1,2]- and [2,3]-rearrangement for the propargylic derivative.

From intermediate **II** (Scheme 3), the two different possibilities, that is [1,2]- and [2,3]-rearrangement, have been explored. In the former, a single transition state (**TS1**) is necessary to obtain the final product, but the activation free energy is quite high (ΔG^ǂ^ = 49 kcal/mol) due to the strain of the incipient three-members ring in **TS1** (Figure 1). On the other hand, for the [2,3]-rearrangement, two different TSs (**TS2** and **TS3**) are necessary. In the former, the carbanion of **II** attacks the terminal propargylic carbon giving the conjugated base of 9-alkyl-7-aryl-1-oxa-7,8-diazaspiro[4.4]nona-3,8-dien-6-one **IV**. Successively, in **TS3**, the bond between the oxygen and the carbon 1 breaks, giving **V**, which after reprotonation, will lead to **4a**. The free energy of **TS2** and **TS3** are quite similar (11.1 and 10.4 kcal/mol, respectively).

The energy profile for the two mechanisms (Figure 1) shows that the [2,3]-rearrangement is largely favored over the other one, both thermodynamically and kinetically, de facto excluding that a [1,2]-Wittig rearrangement may be involved in the concomitant formation of **5**. So, the formation of the 9-alkyl-7-aryl-1-oxa-7,8-diazaspiro[4.4]nona-3,8-dien-6-ones **5d**–**g** happens by means of the protonation of intermediate **IV** (Scheme 2, Figure 1).

However, the high activation energy determined by the DFT study for the formation of final products **4** and **5** can explain why their yields are so low. On the other hand, it is reported in the literature that the yields of Wittig rearrangement products are commonly quite low [63,64,65,66,67,68,69,70,71,72,73,74,75,76,77,78,79,80,81,82,83,84].

It is noteworthy that, commonly to trigger the Wittig rearrangements, the use of strong Brønsted bases, such as BuLi or *t*-BuLi [63,64,65,66,67,68,69,70,71,72,73,74,75,76,77,78,79,80,81,82,83,84], is required, and usually, the reactions happen at very low temperatures. In our system instead, much milder conditions, such as a weak base as K_2_CO_3_ and a temperature of 60 °C, are able to efficiently promote the α-deprotonation and the consequent [2,3]-Wittig rearrangement. This aspect together with the possibility to conduct the synthesis under solvent-free conditions makes it eco-friendly and less harmful to the environment.

It is well known that allyl alcohol is a valuable building block in organic syntheses, due to its versatile reactivity as alkylating agent [82,83,84,85].

**Scheme 4:**
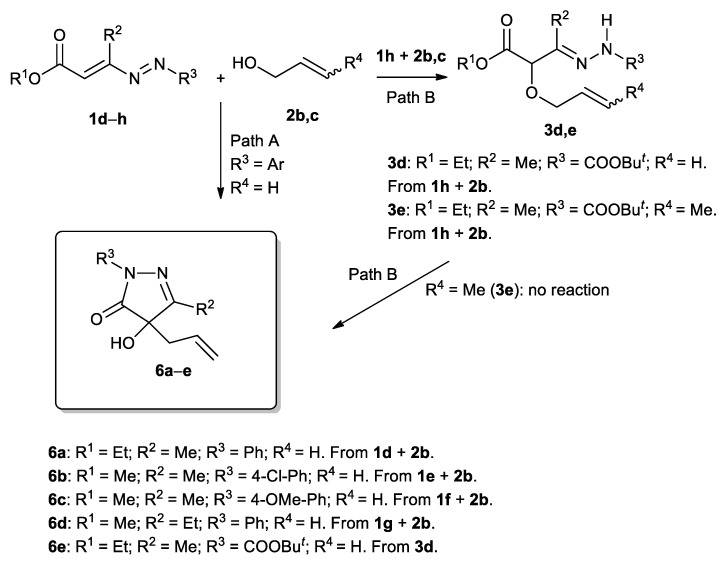
Synthesis of hydrazonic adducts **3d**,**e**, 4-allyl-4-hydroxy-3-alkyl-1-aryl-1*H*-pyrazol-5(4*H*)-ones **6a**–**d** and *tert*-butyl 4-allyl-4-hydroxy-3-methyl-5-oxo-4,5-dihydro-1*H*-pyrazole-1-carboxylate **6e**.

For this fact and also with the intent to verify if the mild conditions employed for [2,3]-Wittig rearrangement in the reaction of the DDs **1** with propargyl alcohol **2a** could be extended to other substrates, we have planned to conduct the reaction between DDs **1d**–**h** and allyl/crotyl alcohol **2b**,**c** (Scheme 4). To our great pleasure, the reactions conducted using **2b**, as solvent and reagent, at 60 °C and in the presence of 4 equiv. of K_2_CO_3_, have actually provided the corresponding 4-allyl-4-hydroxy-3-alkyl-1aryl-1*H*-pyrazol-5(4*H*)-ones **6a**–**e** (25–70%) (Scheme 4, Path A, Table 3).

**Scheme 5:**
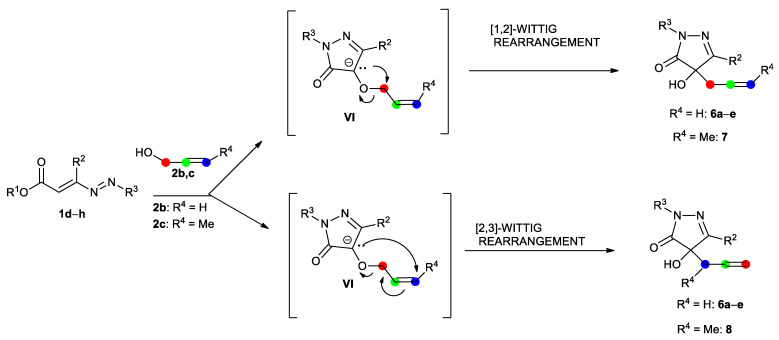
Possible [1,2]- or [2,3]-Wittig rearrangements of 4-(allyloxy)-1H-pyrazol-5(4H)-ones **III**.

Also in this case, the reaction proceeds through the preliminary formation of a hydrazonic adduct intermediate **3**, by means of the nucleophilic attack of the oxygen of allyl alcohol **2** to the terminal carbon atom of the azoene system of the DD **1**, followed by a base-promoted cyclization due to a nucleophilic attack of the hydrazonic nitrogen at the ester group of hydrazone **3**, with the loss of an alcohol molecule, to give the corresponding non-isolable pyrazolone **VI** (Scheme 5). Now, the base-promoted loss of the hydrogen in the 4 position of the pyrazolone ring can theoretically promote both [1,2]- and [2,3]-Wittig rearrangements, however, furnishing in both cases the same final products **6a**–**d** (Scheme 4 and Scheme 5) [17,18].

So, to clarify which of the two mechanisms was involved, we tried to introduce a methyl substituent on the terminal carbon atom of the double bond of the alcohol and therefore we tested the crotyl alcohol **2c** (R^4^ = Me, Scheme 4 and Scheme 5) in the reaction with 1-aryl-DDs **1d**–**g**. To our large disappointment, in all cases, the reaction was unsuccessful, despite using various conditions of solvent, base, and temperature (for the conditions tested, see Table S1 in the Supplementary Materials).

We have then investigated the behavior of *t*-butoxycarbonyl-DD **1h**, chosen as an example, by virtue of its incremented electrophilic character due to the replacement of the aryl on the N1 of the azo-ene system with the BOC moiety [62].

As supposed, both allyl (**2b**) and crotyl (**2c**) alcohol reacted with **1h**, at room temperature, with only one equivalent of K_2_CO_3_, under solvent-free conditions, furnishing the hydrazonic adduct intermediates **3d**,**e** (Scheme 4, Path B and Scheme 5, Table 3). Unfortunately, while compound **3d**, if treated with 1 equiv. of K_2_CO_3_ in ethanol, was converted into the corresponding 4-allyl-4-hydroxy-3-alkyl-1-alcoxycarbonyl-1*H-*pyrazol-5(4*H*)-one **6e**, **3e** did not furnish the corresponding pyrazol-5(4*H*)-one under any of the countless conditions tested (Scheme 4, Path B and Scheme 5, Table 3. For the conditions tested, see Table S2 in the Supplementary Materials).

**Figure 2:**
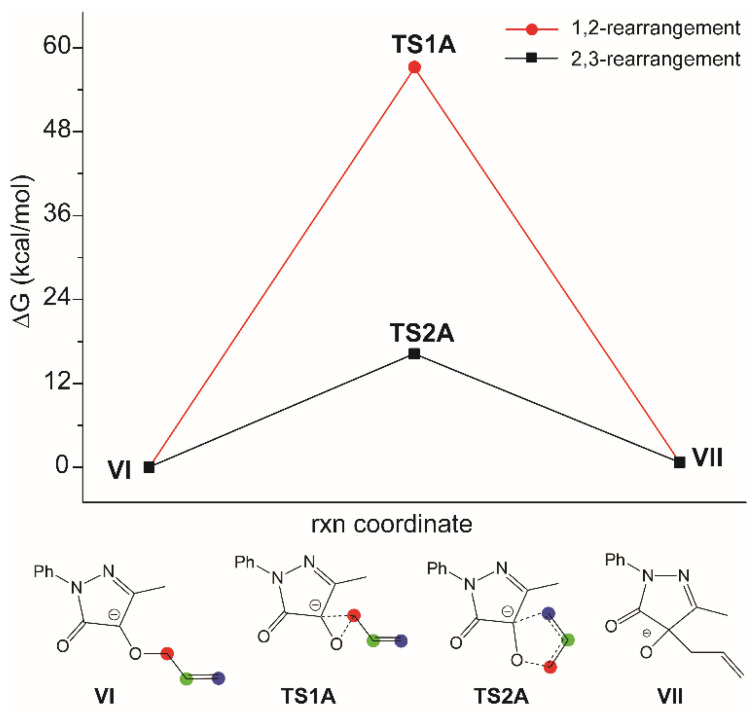
Energy profile for the [1,2]- and [2,3]-rearrangement for the allylic derivative.

Then, a DFT study was carried out for the reaction between the allylic moiety and DDs, for which, given the lack of reactivity of substituted allyl alcohol, it is difficult to obtain experimental information about the mechanism. Also, in this case, the strain in the TS of the [1,2]-rearrangement (**TS1A**) makes this path high in energy (ΔGǂ = 57.1 kcal/mol) and not a viable option. Differently than before, the [2,3]-rearrangement is not a two-step mechanism, as the formation of the Cα-C1 bond and the O-C1 bond cleavage are concerted (**TS2A**). The activation barrier of the [2,3]-rearrangement is 16.2 kcal/mol, which is higher than in the case of the propargylic moiety but still viable, in principle (Figure 2).

Both the mechanisms lead to the same product, **VII**, but the latter is less stable than the starting material, **VI**, by 1.4 kcal/mol. This difference is positive and almost constant with all the dielectric values used in the calculations, ranging from toluene to water.

Using the crotyl alcohol, two different products, **7** or **8**, are possible, depending on the reaction mechanism (Scheme 5). Both the [1,2]- and [2,3]-rearrangement would lead to a thermodynamically unfavored product (ΔG° = 2.8 and 7.3 kcal/mol).

Finally, if using 1-t-butoxycarbonyl-DD **1h** instead of the 1-aryl-ones, the framework is slightly different. In this case, the use of allyl alcohol leads to a product that is thermodynamically favored (**9**, ΔG° = −1.1 kcal/mol) and it is the same with both the [1,2]- and [2,3]-rearrangements. Given the previous results (Figure 2), it is most likely that only the [2,3]-rearrangement is active. The use of crotyl alcohol leads to a slightly different scenario: the [1,2]-rearrangement would lead to a substantially thermoneutral product (10, ΔG° = −0.09 kcal/mol), but, as seen before, this way is kinetically forbidden, whereas the [2,3]-rearrangement, which would be kinetically viable, leads to a product that is thermodynamically forbidden (**11**, ΔG° = 6.7 kcal/mol) (see Supp. Information).

## 3. Experimental Section

### 3.1. General

All chemicals and solvents were purchased from commercial suppliers and used as received. 1,2-Diaza-1,3-dienes were prepared as reported [86,87,88] and used as *EE*/*EZ* isomer mixtures. Melting points were determined in open capillary tubes and are uncorrected. FTIR spectra were obtained as Nujol mulls. All ^1^H NMR and ^13^C NMR spectra were recorded at 400 and 100 MHz, respectively. Proton and carbon spectra were referenced internally to solvent signals, using values of δ = 7.27 ppm for proton and δ = 77.00 ppm for carbon (middle peak) in CDCl_3_. All coupling constants (*J*) are given in Hz. All the NH and OH exchanged with D_2_O. Precoated silica gel plates of 0.25 mm were employed for analytical thin-layer chromatography. All new compounds showed satisfactory elemental analysis. Mass spectra were recorded in the ESI-MS mode. The nomenclature was generated using ACD/IUPAC Name (version 3.50, 5 April 1998), Advanced Chemistry Development Inc., Toronto, ON, Canada.

### 3.2. General Procedure for the Synthesis of 3-Alkyl-4-hydroxy-1-aryl-4-(propa-1,2-dienyl) 1H-Pyrazol-5(4H)-ones ***4a**–**d*** and of 9-Alkyl-7-aryl-1-oxa-7,8-diazaspiro[4.4]nona-3,8-dien-6-ones ***5d–g***, Starting from 1,2-Diaza-1,3-dienes ***1d–g*** and Propargyl Alcohol ***2a***

To a magnetically stirred mixture of 1,2-diaza-1,3-diene **1d–g** (1 mmol) and propargyl alcohol **2a** (3 mL) at 60 °C (oil bath), K_2_CO_3_ (4 mmol) was added and the suspension was left to stand under these conditions for the appropriate time (8.0–11.5 h) until the disappearance of the reagent 1 (TLC monitoring). The crude mixture was then purified by column chromatography on silica gel and successively on thin-layer chromatography to afford 3-alkyl-4-hydroxy-1-aryl-4-(propa-1,2-dienyl)1*H*-pyrazol-5(4*H*)-ones **4a–d** and 9-alkyl-7-aryl-1-oxa-7,8-diazaspiro[4.4]nona-3,8-dien-6-ones **5d–g**. Compounds **4b,d** and **5e,g** were crystallized from EtOAc-light petroleum ether, while **4a,c** and **5d,f** were found to be oils.

*4-Hydroxy-3-methyl-1-phenyl-4-(propa-1,2-dienyl)-1H-pyrazol-5(4H)-one (***4a***).*

**4a** was isolated by column chromatography (acetate/cyclohexane 20:80) and then by thin-layer chromatography (three elutions in acetate/cyclohexane 20:80 mixture) in 11% yield as brown oil; ^1^H-NMR (400 MHz, CDCl_3_, 25 °C): δ 2.21 (s, 3H), 4.09 (brs, 1H), 5.09 (d, *J* = 6.4, 2H), 5.40 (t, *J* = 6.4 Hz, 1H), 7.20 (t, *J* = 7.6, 1H_ar_), 7.40 (t, *J* = 7.2 Hz, 2H_ar_), 7.86 (d, *J* = 8.8 Hz, 2H_ar_) ppm; ^13^C-NMR (100 MHz, CDCl_3_, 25 °C): δ 13.3 (q), 78.0 (s), 80.7 (t), 89.7 (d), 118.8 (d), 125.3 (d), 128.8 (d), 137.6 (s), 161.2 (s), 172.4 (s), 207.6 (s) ppm; IR (Nujol, cm^−1^): ν_max_ 3302, 1953, 1719; MS (ESI): *m*/*z* 229.25 [M + H^+^]; anal. calcd. for C_13_H_12_N_2_O_2_ (228.25): C 68.41, H 5.30, N 12.27; found: C 68.58, H 5.35, N 12.18.

*1-(4-Chlorophenyl)-4-hydroxy-3-methyl-4-(propa-1,2-dienyl)-1H-pyrazol-5(4H)-one (***4b***).*

**4b** was isolated by column chromatography (acetate/cyclohexane 20:80) and then by thin-layer chromatography (three elutions in acetate/cyclohexane 20:80 mixture) in 10% yield as a brown solid; mp: 85–87 °C; ^1^H-NMR (400 MHz, CDCl_3_, 25 °C): δ = 2.20 (s, 3H), 4.28–4.30 (m, 1H), 5.13 (d, *J* = 6.4 Hz, 2H), 5.36 (t, *J* = 6.4 Hz, 1H), 7.36 (d, *J* = 8.8 Hz, 2H_ar_), 7.83 (d, J = 9.2 Hz, 2H_ar_) ppm; ^13^C-NMR (100 MHz, CDCl3, 25 °C): δ 13.3 (q), 77.6 (s), 81.3 (t), 89.6 (d), 119.8 (d), 128.9 (d), 130.4 (s), 136.2 (s), 160.9 (s), 171.8 (s), 207.6 (s) ppm; IR (Nujol, cm^−1^): ν_max_ 3302, 1953, 1719; MS (ESI): *m*/*z* 261.22 [M − H^+^]; anal. calcd. for C_13_H_11_N_2_O_2_Cl (262.69): C 59.44, H 4.22, N 10.66; found: C 59.21, H 4.28, N 10.60.

*4-Hydroxy-1-(4-methoxyphenyl)-3-methyl-4-(propa-1,2-dienyl)-1H-pyrazol-5(4H)-one (***4c***).*

**4c** was isolated by column chromatography (acetate/cyclohexane 20:80) and then by thin-layer chromatography (three elutions in acetate/cyclohexane 20:80 mixture) in 14% yield as brown oil; ^1^H-NMR (400 MHz, CDCl_3_, 25 °C): δ 2.20 (s, 3H), 3.83 (s, 3H), 4.29 (d, *J* = 2.4 Hz, 1H), 5.15 (d, *J* = 6.8 Hz, 2H), 5.35 (t, *J* = 6.8 Hz, 1H), 6.94 (d, *J* = 9.2 Hz, 2H_ar_), 7.74 (d, *J* = 9.2 Hz, 2H_ar_) ppm; ^13^C-NMR (100 MHz, CDCl_3_, 25 °C): δ 13.2 (q), 55.5 (q), 77.2 (s), 81.2 (t), 89.9 (d), 114.1 (d), 120.6 (d), 130.6 (s), 157.2 (s), 160.4 (s), 171.4 (s), 207.6 (s) ppm; IR (Nujol, cm^−1^): ν_max_ 3242, 1956, 1712, 1678; MS (ESI): *m*/*z* 259.12 [M + H^+^]; anal. calcd. for C_14_H_14_N_2_O_3_ (258.27): C 65.11, H 5.46, N 10.85; found: C 65.22, H 5.40, N 10.90.

*3-Ethyl-4-hydroxy-1-phenyl-4-(propa-1,2-dienyl)-1H-pyrazol-5(4H)-one (***4d***).*

**4d** was isolated by column chromatography (acetate/cyclohexane 20:80) and then by thin-layer chromatography (three elutions in acetate/cyclohexane 20:80 mixture) in 18% yield as an orange solid; mp: 101–103; ^1^H-NMR (400 MHz, CDCl_3_, 25 °C): δ 1.32 (t, *J* = 7.2 Hz, 3H), 2.55–2.66 (m, 2H), 4.27–4.29 (m, 1H), 5.11 (d, *J* = 6.8 Hz, 2H), 5.37 (t, *J* = 6.4 Hz, 1H), 7.21 (t, *J* = 7.2 Hz, 2H_ar_), 7.41 (t, *J* = 7.6 Hz, 2H_ar_), 7.89 (d, *J* = 8. Hz, 1H_ar_) ppm; ^13^C-NMR (100 MHz, CDCl_3_, 25 °C): δ = 9.3 (q), 21.0 (t), 78.1 (s), 81.1 (t), 90.1 (d), 118.8 (d), 125.3 (d), 128.8 (d), 137.7 (s), 164.6 (s), 172.4 (s), 207.5 (s) ppm; IR (Nujol, cm^−1^): ν_max_ 3279, 1956, 1691; MS (ESI): *m*/*z* 243.12 [M + H^+^]; anal. calcd. for C_14_H_14_N_2_O_2_ (242.27): C 69.41, H 5.82, N 11.56; found: C 69.32, H 5.89, N 11.65.

*9-Methyl-7-phenyl-1-oxa-7,8-diazaspiro[4.4]nona-3,8-dien-6-one (***5d***).*

**5d** was isolated by column chromatography (acetate/cyclohexane 20:80) and then by thin-layer chromatography (three elutions in acetate/cyclohexane 20:80 mixture) in 29% yield as orange oil; ^1^H-NMR (400 MHz, CDCl_3_, 25 °C): δ 2.09 (s, 3H), 4.94–4.99 and 5.12–5.16 (2m, 2H), 5.62–5.65 (m, 1H), 6.46–6.48 (m, 1H), 7.19 (t, *J* = 7.6 Hz, 1H_ar_), 7.40 (t, *J* = 7.6 Hz, 2H_ar_), 7.89 (dd, *J* = 8.4 Hz, *J* = 1.2, 2H_ar_) ppm; ^13^C-NMR (100 MHz, CDCl_3_, 25 °C): δ = 13.1 (q), 78.4 (t), 93.7 (s), 118.5 (d), 123.8 (d), 125.1 (d), 128.8 (d), 133.5 (d), 138.0 (s), 159.8 (s), 170.8 (s) ppm; IR (Nujol, cm^−1^): ν_max_ 3314, 3081, 1762, 1597; MS (ESI): *m*/*z* 227.20 [M − H^+^]; anal. calcd. for C_13_H_12_N_2_O_2_ (228.25): C 68.41, H 5.30, N 12.27; found: C 68.31, H 5.38, N 12.39.

*7-(4-Chlorophenyl)-9-methyl-1-oxa-7,8-diazaspiro[4.4]nona-3,8-dien-6-one (***5e***).*

**5e** was isolated by column chromatography (acetate/cyclohexane 20:80) and then by thin-layer chromatography (three elutions in acetate/cyclohexane 20:80 mixture) in 17% yield as an orange solid; mp: 108–109 °C; ^1^H-NMR (400 MHz, CDCl_3_, 25 °C): δ 2.08 (s, 3H), 4.94–4.98 and 5.11–5.15 (2m, 2H), 5.62–5.64 (m, 1H), 6.47–6.49 (m, 1H), 7.36 (d, *J* = 9.2 Hz, 2H_ar_), 7.86 (d, *J* = 9.2 Hz, 2H_ar_) ppm; ^13^C-NMR (100 MHz, CDCl_3_, 25 °C): δ 13.1 (q), 78.4 (t), 93.6 (s), 119.6 (d), 123.6 (d), 128.9 (d), 130.1 (s), 133.7 (d), 136.6 (s), 160.1 (s), 170.7 (s) ppm; IR (Nujol, cm^−1^): ν_max_ = 3278, 3093, 1722, 1595; MS (ESI): *m*/*z* 261.22 [M − H^+^]; anal. calcd. for C_13_H_11_N_2_O_2_Cl (262.69): C 59.44, H 4.22, N 10.66; found: C 59.56, H 4.28, N 10.74.

*7-(4-Methoxyphenyl)-9-methyl-1-oxa-7,8-diazaspiro[4.4]nona-3,8-dien-6-one (***5f***).*

**5f** was isolated by column chromatography (acetate/cyclohexane 20:80) and then by thin-layer chromatography (three elutions in acetate/cyclohexane 20:80 mixture) in 21% yield as brown oil; ^1^H-NMR (400 MHz, CDCl_3_, 25 °C): δ 2.08 (s, 3H), 3.82 (s, 3H), 4.94–4.98 and 5.12–5.16 (2m, 2H), 5.62–5.65 (m, 1H), 6.46–6.48 (m, 1H), 6.93 (d, *J* = 9.2 Hz, 2H_ar_), 7.77 (d, *J* = 9.2 Hz, 2H_ar_) ppm; ^13^C-NMR (100 MHz, CDCl_3_, 25 °C): δ 13.1 (q), 55.5 (q), 78.4 (t), 93.6 (s), 114.0 (d), 120.4 (d), 123.8 (d), 131.4 (s), 133.4 (d), 157.0 (s), 159.7 (s), 170.4 (s) ppm; IR (Nujol, cm^−1^): ν_max_ 3279, 3086, 1731, 1717, 1610; MS (ESI): *m*/*z* 257.13 [M − H^+^]; anal. calcd. for C_14_H_14_N_2_O_3_ (258.27): C 65.11, H 5.46, N 10.85; found: C 64.99, H 5.54, N 10.76.

*9-Ethyl-7-phenyl-1-oxa-7,8-diazaspiro[4.4]nona-3,8-dien-6-one (***5g***).*

**5g** was isolated by column chromatography (acetate/cyclohexane 20:80) and then by thin-layer chromatography (three elutions in acetate/cyclohexane 20:80 mixture) in 18% yield as a yellow solid; mp: 121–123 °C; ^1^H-NMR (400 MHz, CDCl_3_, 25 °C): δ 1.27 (t, *J* = 7.2 Hz, 3H), 2.34–2.54 (m, 2H), 4.93–4.97 and 5.11–5.15 (2m, 2H), 5.63–5.66 (m, 1H), 6.44–6.46 (m, 1H), 7.19 (t, *J* = 7.6 Hz, 1H_ar_), 7.41 (t, *J* = 7.6 Hz, 2H_ar_), 7.92 (dd, *J* = 8.8 Hz, *J* = 1.2, 2H_ar_) ppm; ^13^C-NMR (100 MHz, CDCl_3_, 25 °C): δ 9.7 (q), 21.1 (t), 78.2 (t), 93.7 (s), 118.5 (d), 124.1 (d), 125.0 (d), 128.8 (d), 133.1 (d), 138.0 (s), 163.5 (s), 170.9 (s) ppm; IR (Nujol, cm^−1^): ν_max_ 3096, 1714, 1632, 1597; MS (ESI): *m*/*z* 241.25 [M − H^+^]; anal. calcd. for C_14_H_14_N_2_O_2_ (242.27): C 69.41, H 5.82, N 11.56; found: C 69.31, H 5.77, N 11.62.

### 3.3. General Procedure for the Synthesis of Tert-Butyl 2-(3-(Allyloxy)-4-ethoxy-4-oxobutan-2-ylidene) Hydrazinecarboxylate (***3d***) or Tert-Butyl 2-(3-(but-2-en-1-yloxy)-4-ethoxy-4-oxobutan-2-ylidene) Hydrazinecarboxylate (***3e***), Starting from 1,2-Diaza-1,3-diene ***1h*** and Allyl (***2b***) or Crotyl (***2c***) Alcohol

To a magnetically stirred mixture of 1,2-diaza-1,3-diene **1h** (1 mmol) and allyl (**2b**) or crotyl (**2c**) alcohol (10 equiv.) at room temperature, K_2_CO_3_ (1 mmol) was added and the suspension was left to stand under these conditions for 0.1 h until the disappearance of the reagent 1 (TLC monitoring). The crude mixture was quickly filtered and then purified by column chromatography on silica gel to afford compounds **3d,e**, that were crystallized from EtOAc.

*Tert-butyl 2-(3-(allyloxy)-4-ethoxy-4-oxobutan-2-ylidene)hydrazinecarboxylate (***3d***).*

**3d** was isolated by column chromatography (acetate/cyclohexane 40:60) in 37% yield as a white solid; mp: 119–120 °C; ^1^H-NMR (400 MHz, CDCl_3_, 25 °C): δ 1.27 (t, *J* = 7.2 Hz, 3H), 1.52 (s, 9H), 1.83 (s, 3H), 4.02–4.12 (m, 2H), 4.20–4.28 (m, 2H), 4.63 (s, 1H), 5.21 (d, *J* = 10.0 Hz, 1H), 5.28–5.35 (m, 1H), 5.86–5.95 (m, 1H), 7.60 (s, 1H) ppm; ^13^C-NMR (100 MHz, CDCl_3_, 25 °C): δ 10.8 (q), 14.1 (q), 28.2 (q), 61.4 (t), 70.8 (t), 81.6 (s), 81.8 (d), 118.2 (t), 133.5 (d), 146.8 (s), 152.3 (s), 169.1 (s) ppm; IR (Nujol, cm^−1^): ν_max_ 3235, 1760, 1725, 1704; MS (ESI): *m*/*z* 303.22 [M + H^+^]; anal. calcd. for C_14_H_24_N_2_O_5_ (302.17): C 55.98, H 8.05, N 9.33; found: C 56.16, H 8.40, N 9.18.

*Tert-butyl 2-(3-(but-2-en-1-yloxy)-4-ethoxy-4-oxobutan-2-ylidene)hydrazinecarboxylate (***3e***).*

**3e** was isolated by column chromatography (acetate/cyclohexane 40:60) in 22% yield as a white solid; mp: 158–160 °C; ^1^H-NMR (400 MHz, CDCl_3_, 25 °C): δ 1.27 (t, *J* = 7.2 Hz, 3H), 1.52 (s, 9H), 1.71 (dd, *J* = 6.4 Hz, *J* = 0.8 Hz, 3H), 1.82 (s, 3H), 3.96–4.01 (m, 2H), 4.22 (q, *J* = 7.2 Hz, 2H), 4.62 (s, 1H), 5.53–5.61 (m, 1H), 5.70–5.79 (m, 1H), 7.57 (s, 1H) ppm; ^13^C-NMR (100 MHz, CDCl_3_, 25 °C): δ 10.8 (q), 14.2 (q), 17.8 (q), 28.2 (q), 61.4 (t), 70.6 (t), 81.4 (d), 81.5 (s), 126.3 (d), 131.1 (d), 146.9 (s), 152.2 (s), 169.3 (s) ppm; IR (Nujol, cm^−1^): ν_max_ 3372, 3269, 1715, 1627; MS (ESI): *m*/*z* 313.09 [M − H^+^]; anal. calcd. for C_15_H_26_N_2_O_5_ (314.18): C 57.31, H 8.34, N 8.91; found: C 57.16, H 8.41, N 8.98.

### 3.4. General Procedure for the Synthesis of 4-Allyl-4-hydroxy-3-alkyl-1-aryl-1H-pyrazol-5(4H)-ones ***6a–d***, Starting from 1,2-Diaza-1,3-dienes ***1d–g*** and Allyl Alcohol ***2b***

To a magnetically stirred mixture of 1,2-diaza-1,3-dienes **1d–g** (1 mmol) and allyl alcohol **2b** (3 mL) at 60 °C (oil bath), K_2_CO_3_ (4 mmol) was added and the suspension was left to stand under these conditions for the appropriate time (8.0–16.0 h), until the disappearance of the reagent **1** (TLC monitoring). The crude mixture was then purified by column chromatography on silica gel to afford 4-allyl-4-hydroxy-3-alkyl-1-aryl-1*H*-pyrazol-5(4*H*)-ones **6a–d** as oils, in the case of **6a,c,d** or solid that was crystallized from EtOAc-light petroleum ether in the case of **6b**.

### 3.5. General Procedure for the Synthesis of 4-Allyl-4-hydroxy-3-methyl-1-alcoxycarbonyl-1H-pyrazol-5(4H)-one ***6e***, Starting from ***3d***

To a magnetically stirred solution of *tert*-butyl 2-(3-(allyloxy)-4-ethoxy-4-oxobutan -2-ylidene)hydrazinecarboxylate **3d** (1 mmol) in ethanol (3 mL), K_2_CO_3_ (1 mmol) was added and the suspension was left to stand under these conditions for 2.0 h, until the disappearance of the reagent 3 (TLC monitoring). The crude mixture was then filtered and purified by column chromatography on silica gel to afford 6e as oil.

*4-Allyl-4-hydroxy-3-methyl-1-phenyl-1H-pyrazol-5(4H)-one (***6a***).*

**6a** was isolated by column chromatography (acetate/cyclohexane 20:80) in 25% yield as brown oil; ^1^H-NMR (400 MHz, CDCl_3_, 25 °C): δ 2.18 (s, 3H), 2.59–2.73 (m, 2H), 3.90 (brs, 1H), 5.15–5.24 (m, 2H), 5.55–5.65 (m, 1H), 7.20 (t, *J* = 7.6 Hz, 1H_ar_), 7.39 (t, *J* = 8.8 Hz, 2H_ar_), 7.83 (d, _J_ = 7.6 Hz, 2H_ar_) ppm; ^13^C-NMR (100 MHz, CDCl_3_, 25 °C): δ 13.2 (q), 40.6 (t), 79.6 (s), 118.9 (d), 121.4 (t), 125.4 (d), 128.5 (d), 128.8 (d), 137.4 (s), 161.5 (s), 173.4 (s) ppm; IR (Nujol, cm^−1^): ν_max_ 3263, 1683, 1596; MS (ESI): *m*/*z* 231.30 [M + H^+^]; anal. calcd. for C_13_H_14_N_2_O_2_ (230.26): C 67.81, H 6.13, N 12.17; found: C 67.70, H 6.19, N 12.22.

*4-Allyl-1-(4-chlorophenyl)-4-hydroxy-3-methyl-1H-pyrazol-5(4H)-one (***6b***).*

**6b** was isolated by column chromatography (acetate/cyclohexane 20:80) in 33% yield as a yellow solid crystallized from EtOAc-light petroleum ether; mp: 131–133 °C; ^1^H-NMR (400 MHz, CDCl_3_, 25 °C): δ 2.18 (s, 3H), 2.58–2.71 (m, 2H), 4.19 (brs, 1H), 5.15–5.24 (m, 2H), 5.52–5.62 (m, 1H), 7.33 (d, *J* = 8.8 Hz, 2H_ar_), 7.79 (d, *J* = 8.8 Hz, 2H_ar_) ppm; ^13^C-NMR (100 MHz, CDCl_3_, 25 °C): δ 13.2 (q), 40.5 (t), 79.6 (s), 119.9 (d), 121.5 (t), 128.3 (d), 128.9 (d), 130.5 (s), 135.9 (s), 161.9 (s), 173.5 (s) ppm; IR (Nujol, cm^−1^): ν_max_ 3302, 1685, 1625; MS (ESI): *m*/*z* 265.09 [M + H^+^]; anal. calcd. for C_13_H_13_N_2_O_2_Cl (264.71): C 58.99, H 4.95, N 10.58; found: C 59.11, H 4.89, N 10.66.

*4-Allyl-4-hydroxy-1-(4-methoxyphenyl)-3-methyl-1H-pyrazol-5(4H)-one (***6c***).*

**6c** was isolated by column chromatography (acetate/cyclohexane 20:80) in 33% yield as brown oil; ^1^H-NMR (400 MHz, CDCl_3_, 25 °C): δ 2.17 (s, 3H), 2.60–2.64 (m, 2H), 3.82 (s, 3H), 4.10 (brs, 1H), 5.20–5.27 (m, 2H), 5.60–5.69 (m, 1H), 6.93 (d, *J* = 9.2 Hz, 2H_ar_), 7.72 (d, *J* = 9.2 Hz, 2H_ar_) ppm; ^13^C-NMR (100 MHz, CDCl_3_, 25 °C): δ 13.3 (q), 40.7 (t), 55.5 (q), 79.0 (s), 114.0 (d), 120.7 (d), 121.5 (t), 128.5 (d), 130.8 (s), 157.2 (s), 160.8 (s), 172.5 (s) ppm; IR (Nujol, cm^−1^): ν_max_ 3357, 1693, 1608; MS (ESI): *m*/*z* 259.23 [M − H^+^]; anal. calcd. for C_14_H_16_N_2_O_3_ (260.29): C 64.60, H 6.20, N 10.76; found: C 64.51, H 6.24, N 10.67.

*4-Allyl-3-ethyl-4-hydroxy-1-phenyl-1H-pyrazol-5(4H)-one (***6d***).*

**6d** was isolated by column chromatography (acetate/cyclohexane 20:80) in 28% yield as red oil; ^1^H-NMR (400 MHz, CDCl_3_, 25 °C): δ 1.32 (t, *J* = 7.2 Hz, 3H), 2.44–2.70 (m, 4H), 3.65 (brs, 1H), 5.16–5.25 (m, 2H), 5.56–5.66 (m, 1H), 7.20 (t, *J* = 7.2 Hz, 1H_ar_), 7.40 (t, *J* = 8.4 Hz, 2H_ar_), 7.87 (dd, *J* = 8.8 Hz, *J* = 1.2 Hz, 2H_ar_) ppm; ^13^C NMR (100 MHz, CDCl_3_, 25 °C): δ 9.1 (q), 21.0 (t), 40.9 (t), 79.7 (s), 118.8 (d), 121.4 (t), 125.2 (d), 128.6 (d), 128.8 (d), 137.6 (s), 164.9 (s), 173.5 (s) ppm; IR (Nujol, cm^−1^): ν_max_ 3313, 1689, 1625, 1597; MS (ESI): *m*/*z* 245.14 [M + H^+^]; anal. calcd. for C_14_H_16_N_2_O_2_ (244.29): C 67.83, H 6.60, N 11.47; found: C 67.71, H 6.65, N 11.54.

*Tert-butyl 4-allyl-4-hydroxy-3-methyl-5-oxo-4,5-dihydro-1H-pyrazole-1-carboxylate (***6e***).*

**6e** was isolated by column chromatography (acetate/cyclohexane 20:80) in 70% yield as pale yellow oil; ^1^H-NMR (400 MHz, CDCl_3_, 25 °C): δ 1.59 (s, 9H), 2.13 (s, 3H), 2.55 (d, *J* = 7.2 Hz, 2H), 3.10 (brs, 1H), 5.20–5.30 (m, 2H), 5.61–5.71 (m, 1H) ppm; ^13^C-NMR (100 MHz, CDCl_3_, 25 °C): δ 13.4 (q), 28.0 (q), 40.3 (t), 78.1 (s), 84.9 (s), 122.2 (t), 127.9 (d), 147.5 (s), 161.5 (s), 173.2 (s) ppm; IR (Nujol, cm^−1^): ν_max_ 3268, 1678, 1619; MS (ESI): *m*/*z* 253.22 [M − H^+^]; anal. calcd. for C_12_H_18_N_2_O_4_ (254.28): C 56.68, H 7.13, N 11.02; found: C 56.84, H 7.02, N 10.86.

## 4. DFT Calculations

All the geometries have been optimized with ORCA 4.1.0 [89,90], using the BP86 functional in conjunction with a def2-TZVP basis set for all the atoms. Dispersion forces were taken into account using the D3 correction with Becke−Johnson damping [91]. The effect of the solvent has been simulated through the Continuum-like Polarizable Continuum Model (C-PCM, dichloromethane if not otherwise specified). All the geometries have been confirmed to be stationary points, with zero (intermediates) or one (transition states) imaginary frequency.

## 5. Conclusions

In conclusion, we have established a protocol to synthesize new and appealing 4-hydroxy-4-(propa-1,2-dienyl) or 4-hydroxy-4-allyl-1*H*-pyrazol-5(4*H*)-ones and spiro dihydrofuran-pyrazolones, starting from 1,2-diaza-1,3-dienes and propargyl or allyl alcohols under very mild conditions. The transformation shows attractive features since it excludes the employment of strong bases and low temperatures.

The obtained products have potentially interesting properties. In fact, often, the biological activities of molecules that incorporate more scaffolds are not simply attributable to the sum of the characteristics shown by the individual functionalities, but synergistic effects can increase its effectiveness, or induce the manifestation of new characteristics.

Finally, a DFT study carried out on these reactions allowed us to obtain definitive elucidations on the mechanism which involves a [2,3]-Wittig rearrangement.

## Figures and Tables

**Table 1:** Screening of different conditions in the reaction between 1-phenyl-DD **1d** and propargyl alcohol **2a** to obtain 4-hydroxy-3-methyl-1-phenyl-4-(propa-1,2-dienyl)-1*H*-pyrazol-5(4*H*)-one **4a** and 9-methyl-7-phenyl-1-oxa-7,8-diazaspiro[4.4]nona-3,8-dien-6-one **5d** ^a^.

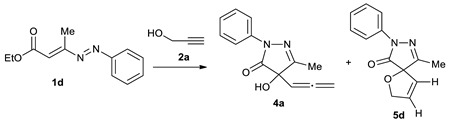					
Entry	Solvent	Base	Equivalents	T	4a; 5d Yield (%) ^b^
1	CH_2_Cl_2_	DBU	0.1	rt	no reaction
2	CH2Cl_2_	DBU	1	rt	complicated mixture ^c^
3	MeCN	DBU	0.1	rt	no reaction
4	MeCN	DBU	0.1	60 °C	complicated mixture ^c^
5	MeCN	DBU	1	rt	complicated mixture ^c^
6	THF	DBU	0.1	rt	no reaction
7	THF	DBU	1	rt	complicated mixture ^c^
8	**2a** as sr ^d^	DBU	1	rt	complicated mixture ^c^
9	MeCN	DIPEA	2	rt	no reaction
10	MeCN	DIPEA	4	rt	no reaction
11	THF	DIPEA	4	rt	no reaction
12	CH_2_Cl_2_	DIPEA	4	rt	no reaction
13	**2a** as sr ^d^	DIPEA	4	rt	no reaction
14	**2a** as sr ^d^	DIPEA	4	60 °C	no reaction
15	MeCN	NaH	0.1	rt	complicated mixture ^c^
16	THF	NaH	0.1	rt	complicated mixture ^c^
17	THF	NaH	0.1	−20 °C	complicated mixture ^c^
18	THF	NaH	0.1	−78 °C	complicated mixture ^c^
19	CH_2_Cl_2_	NaH	0.1	rt	complicated mixture ^c^
20	**2a** as sr ^d^	NaH	0.1	rt	complicated mixture ^c^
21	MeCN	MeONa	0.1	rt	complicated mixture ^c^
22	THF	MeONa	0.1	rt	complicated mixture ^c^
23	THF	MeONa	0.1	−20 °C	complicated mixture ^c^
24	CH_2_Cl_2_	MeONa	0.1	rt	complicated mixture ^c^
25	**2a** as sr ^d^	MeONa	0.1	rt	complicated mixture ^c^
26	THF	*t*-BuONa	0.1	rt	complicated mixture ^c^
27	THF	*t*-BuONa	0.1	−20 °C	complicated mixture ^c^
28	MeCN	K_2_CO_3_	4	rt	complicated mixture ^c^
29	THF	K_2_CO_3_	4	rt	complicated mixture ^c^
30	CH_2_Cl_2_	K_2_CO_3_	4	rt	complicated mixture ^c^
31	**2a** as sr ^d^	K_2_CO_3_	4	rt	complicated mixture ^c^
**32**	**2a** as sr ^d^	**K_2_CO_3_**	**4**	**60 °C**	**4a: 11%; 5d: 29%**
33	**2a** as sr ^d^	Cs_2_CO_3_	4	60 °C	**4a**: 9%; **5d**: 19%
34	**2a** as sr ^d^	Na_2_CO_3_	4	60 °C	**4a**: 6%; **5d**: 11%

^a^ The reactions were performed at 0.5 mmol scale of 1-phenyl-DD **1d** in 3 mL of solvent, using 1.2 equiv. of alcohol **2a** (except when **2a** was employed as solvent/reagent). ^b^ Yields of isolated **4a** and **5d**, based on 1-phenyl-DD **1d**. ^c^ TLC analysis revealed traces of products **4a** and **5d**. ^d^ Propargyl alcohol was used as solvent/reagent (3 mL of **2a** were employed).

**Table 2:** Yields and reaction times for the synthesis of 3-alkyl-4-hydroxy-1-aryl-4-(propa-1,2-dienyl)1*H*-pyrazol-5(4*H*)-ones **4a**–**d** ^a^ and of 9-alkyl-7-aryl-1-oxa-7,8-diazaspiro[4.4]nona-3,8-dien-6-ones **5d**–**g**.

Entry	1	R^1^	R^2^	R^3^	4	Yield (%) ^b^	5	Yield (%) ^c^	Time (h) ^d^
1	**1d**	Et	Me	H	**4a**	11	**5d**	29	11.5
2	**1e**	Me	Me	Cl	**4b**	10	**5e**	17	10.0
3	**1f**	Me	Me	OMe	**4c**	14	**5f**	21	9.0
4	**1g**	Me	Et	H	**4d**	18	**5g**	18	8.0

^a^ Reaction conditions: DDs **1d**–**g** (1 mmol), propargyl alcohol **2a** (3 mL), K_2_CO_3_ (4 mmol), at 60 °C (oil bath). ^b^ Yield of pure isolated 3-alkyl-4-hydroxy-1-aryl-4-(propa-1,2-dienyl)1*H*-pyrazol-5(4*H*)-ones **4a**–**d** referred to **1**. ^c^ Yield of pure isolated 9-alkyl-7-aryl-1-oxa-7,8- diazaspiro[4.4]nona-3,8-dien-6-ones **5d**–**g** referred to **1**. ^d^ Time of disappearance of **1**.

**Table 3:** Yields and reaction times for the synthesis of adducts **3d**,**e**, and 1*H*-pyrazol-5(4*H*)-ones **6a**–**e**.

Entry	1	R^1^	R^2^	R^3^	2	R^4^	3 ^a^	Yield (%) ^b^	Time (h)	6	Yield (%)	Time (h)
1	**1d**	Et	Me	Ph	**2b**	H				**6a** ^c^	25 ^e^	8.0
2	**1e**	Me	Me	4-Cl-Ph	**2b**	H				**6b** ^c^	33 ^e^	10.0
3	**1f**	Me	Me	4-OMe-Ph	**2b**	H				**6c** ^c^	33 ^e^	6.0
4	**1g**	Me	Et	Ph	**2b**	H				**6d** ^c^	28 ^e^	16.0
5	**1h**	Et	Me	COOBu*^t^*	**2b**	H	**3d**	37	0.1	**6e** ^d^	70 ^f^	4.0
6	**1h**	Et	Me	COOBu*^t^*	**2c**	Me	**3e**	22	0.1			

^a^ Reaction conditions: DD **1h** (1 mmol), allyl (**2b**) or crotyl **(2c)** alcohol (3 mL), K_2_CO_3_ (1 mmol), rt. ^b^ Yields of **3d**,**e** referred to **1**. ^c^ Reaction conditions: DDs **1d**–**g** (1 mmol), allyl alcohol **2b** (3 mL), K_2_CO_3_ (4 mmol), at 60 °C (oil bath). ^d^ Reaction conditions: adduct **3d** (1 mmol) in ethanol (3 mL), K_2_CO_3_ (1 mmol), rt. ^e^ Yields of 4-allyl-4-hydroxy-3-alkyl-1-aryl-1*H*-pyrazol-5(4*H*)-ones **6a**–**d** referred to **1**. ^f^ Yield of *tert*-butyl 4-allyl-4-hydroxy-3-methyl-5-oxo-4,5-dihydro-1*H*-pyrazole-1-carboxylate **6e** referred to **3d**.

## Data Availability

The data can be consulted by contacting the correspong authors.

## Supplementary Materials

The following are available online: experimental procedures and spectral data of all compounds, copies of ^1^H-NMR and ^13^C-NMR spectra of compounds **3 d**,**e**, **4a**–**d**, **5d**–**g**, **6a**–**d**, Tables S1 and S2 refer to the screening of conditions in the reaction between DDs **1d**–**g** and crotyl alcohol **2b** and to tentatively convert hydrazone **3e** into the corresponding pyrazolone, respectively. DFT optimized geometries and energies.

## Sample Availability

Samples of the compounds are not available from the authors.
