# Supplementary material for: Experimental and Theoretical DFT Investigations in the [2,3]-Wittig-Type Rearrangement of Propargyl/Allyl-Oxy-Pyrazolones
Source: Molecules. 2021 Oct 29;26(21):6557. doi: 10.3390/molecules26216557 (PMC8587800; doi:10.3390/molecules26216557)
Supplement: Supplementary file 1 [file molecules-26-06557-s001.zip › molecules-1414294-supplementary.pdf]

Experimental and Theorical DFT Investigations in the [2,3]-Wittig-type Rearrangement of Propargyl/Allyl-oxy-Pyrazolones

Lucia De Crescentini,*[a] Gianfranco Favi,[a] Giacomo Mari,[a] Gianluca Ciancaleoni,[b] Marcello Costamagna,[b] Stefania Santeusanio,[a] and Fabio Mantellini*[a]

[a]*Department of Biomolecular Sciences, Section of Organic Chemistry and Organic Natural Compounds, University of Urbino "Carlo Bo", Via I Maggetti 24, 61029 Urbino (PU), Italy*

[a]*Department of Chemistry and Industrial Chemistry, University of Pisa, Via G.Moruzzi 13, 56124 Pisa (PI), Italy*

e-mail: fabio.mantellini@uniurb.it, lucia.decrescentini@uniurb.it

SUPPORTING INFORMATION

Table of Contents

1. General remarks. 2

2. Experimental procedures and spectral data 3–8

General procedure for the synthesis of 3-alkyl-4-hydroxy-1-aryl-4-(propa-1,2-dienyl)1*H*-pyrazol-5(4*H*)-ones **4a–d** and of 9-alkyl-7-aryl-1-oxa-7,8-diazaspiro[4.4]nona-3,8-dien-6-ones **5d–g**, starting from 1,2-diaza-1,3-dienes **1d–g** and propargyl alcohol **2a**. 3

Characterization data of products **4a–d** and **5d–g**. 3

General procedure for the synthesis of *tert*-butyl 2-(3-(allyloxy)-4-ethoxy-4-oxobutan-2-ylidene)hydrazinecarboxylate (**3d**) or *tert*-butyl 2-(3-(but-2-en-1-yloxy)-4-ethoxy-4-oxobutan-2-ylidene)hydrazinecarboxylate (**3e**), starting from 1,2-diaza-1,3-diene **1h** and allyl (**2b**) or crotyl (**2c**) alcohol. 6

Characterization data of products **3d,e**. 6

General procedure for the synthesis of 4-allyl-4-hydroxy-3-alkyl-1-aryl-1*H*-pyrazol-5(4*H*)-ones **6a–d**. 7

General procedure for the synthesis of 4-allyl-4-hydroxy-3-methyl-1-alcoxycarbonyl-1*H*-pyrazol-5(4*H*)-one **6e**, starting from **3d**. 7

Characterization data of products **6a–e**. 7

3. Table S1. 9

4. Table S2. 10

5. ^{1}H and ^{13}C NMR spectra of products **4a–d, 5d–g, 3d,e** and **6a–d**. 12-33

6. DFT optimized geometries and energies 34–53

7. References and notes. 54

1. General Remarks

All chemicals and solvents were purchased from commercial suppliers and used as received. 1,2-Diaza-1,3-dienes were prepared as reported[1] and used as *EE*/*EZ* isomer mixtures. Melting points were determined in open capillary tubes and are uncorrected. FTIR spectra were obtained as Nujol mulls. All ^{1}H NMR and ^{13}C NMR spectra were recorded at 400 and 100 MHz, respectively. Proton and carbon spectra were referenced internally to solvent signals, using values of δ = 7.27 ppm for proton and δ = 77.00 ppm for carbon (middle peak) in $CDCl_3$. All coupling constants (*J*) are given in Hz. All the NH and OH exchanged with D_2O. Precoated silica gel plates 0.25 mm were employed for analytical thin layer chromatography. All new compounds showed satisfactory elemental analysis. Mass spectra were recorded in the ESI-MS mode. The nomenclature was generated using ACD/IUPAC Name (version 3.50, 5 Apr. 1998), Advanced Chemistry Development Inc., Toronto, ON (Canada).

2. Experimental procedures and spectral data

General procedure for the synthesis of 3-alkyl-4-hydroxy-1-aryl-4-(propa-1,2-dienyl)1*H*-pyrazol-5(4*H*)-ones 4a–d and of 9-alkyl-7-aryl-1-oxa-7,8-diazaspiro[4.4]nona-3,8-dien-6-ones 5d–g, starting from 1,2-diaza-1,3-dienes 1d–g and propargyl alcohol 2a.

To a magnetically stirred mixture of 1,2-diaza-1,3-diene **1d–g** (1 mmol) and propargyl alcohol **2a** (3 mL) at 60 °C (oil bath), K_2CO_3 (4 mmol) was added and the suspension was stand under these conditions for the appropriate time (8.0–11.5 hours), until the disappearance of the reagent **1** (TLC monitoring). The crude mixture was then purified by column chromatography on silica gel and then thin layer chromatography to afford 3-alkyl-4-hydroxy-1-aryl-4-(propa-1,2-dienyl)1*H*-pyrazol-5(4*H*)-ones **4a–d** and 9-alkyl-7-aryl-1-oxa-7,8-diazaspiro[4.4]nona-3,8-dien-6-ones **5d–g**, that were oils (**4a,c** and **5d,f**) or solids (**4b,d** and **5e,g**) that were crystallized from EtOAc-light petroleum ether.

Characterization data of products 4a–d and 5d–g.

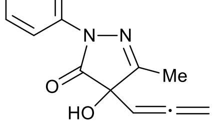

4a

4-Hydroxy-3-methyl-1-phenyl-4-(propa-1,2-dienyl)-1*H*-pyrazol-5(4*H*)-one (4a): **4a** was isolated by column chromatography (acetate/cyclohexane 20:80) and then by thin layer chromatography (three elutions in acetate/cyclohexane 20:80 mixture) in 11% yield. Brown oil; ^{1}H NMR (400 MHz, $CDCl_3$, 25 °C): δ = 2.21 (s, 3H), 4.09 (brs, 1H), 5.09 (d, *J* = 6.4, 2H), 5.40 (t, *J* = 6.4 Hz, 1H), 7.20 (t, *J* = 7.6, $1H_{ar}$), 7.40 (t, *J* = 7.2 Hz, $2H_{ar}$), 7.86 (d, *J* = 8.8 Hz, $2H_{ar}$); ^{13}C NMR (100 MHz, $CDCl_3$, 25 °C): δ = 13.3 (q), 78.0 (s), 80.7 (t), 89.7 (d), 118.8 (d), 125.3 (d), 128.8 (d), 137.6 (s), 161.2 (s), 172.4 (s), 207.6

(s);IR (nujol): ν_{max} = 3302, 1953, 1719 cm^{-1}; MS *m/z* (ESI): 229.25 (M + H^+); anal. calcd. for $C_{13}H_{12}N_2O_2$ (228.25): C 68.41, H 5.30, N 12.27; found: C 68.58, H 5.35, N 12.18.

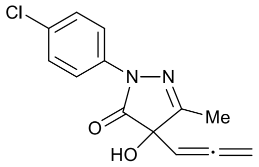

4b

1-(4-Chlorophenyl)-4-hydroxy-3-methyl-4-(propa-1,2-dienyl)-1*H*-pyrazol-5(4*H*)-one (4b): 4b was isolated by column chromatography (acetate/cyclohexane 20:80) and then by thin layer chromatography (three elutions in acetate/cyclohexane 20:80 mixture) in 10% yield. Brown solid; mp: 85–87 °C; ^{1}H NMR (400 MHz, $CDCl_3$, 25 °C): δ = 2.20 (s, 3H), 4.28–4.30 (m, 1H), 5.13 (d, *J* = 6.4 Hz, 2H), 5.36 (t, *J* = 6.4 Hz, 1H), 7.36 (d, *J* = 8.8 Hz, $2H_{ar}$), 7.83 (d, *J* = 9.2 Hz, $2H_{ar}$); ^{13}C NMR (100 MHz, $CDCl_3$, 25 °C): δ = 13.3 (q), 77.6 (s), 81.3 (t), 89.6 (d), 119.8 (d), 128.9 (d), 130.4 (s), 136.2 (s), 160.9 (s), 171.8 (s), 207.6 (s); IR (nujol): ν_{max} = 3302, 1953, 1719 cm^{-1}; MS m/z (ESI): 261.22 (M - H^+); anal. calcd. for $C_{13}H_{11}N_2O_2Cl$ (262.69): C 59.44, H 4.22, N 10.66; found: C 59.21, H 4.28, N 10.60.

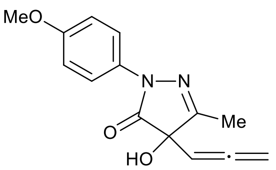

4c

4-Hydroxy-1-(4-methoxyphenyl)-3-methyl-4-(propa-1,2-dienyl)-1*H*-pyrazol-5(4*H*)-one (4c): 4c was isolated by column chromatography (acetate/cyclohexane 20:80) and then by thin layer chromatography (three elutions in acetate/cyclohexane 20:80 mixture) in 14% yield. Brown oil; ^{1}H NMR (400 MHz, $CDCl_3$, 25 °C): δ = 2.20 (s, 3H), 3.83 (s, 3H), 4.29 (d, *J* = 2.4 Hz, 1H), 5.15 (d, *J* = 6.8 Hz, 2H), 5.35 (t, *J* = 6.8 Hz, 1H), 6.94 (d, *J* = 9.2 Hz, $2H_{ar}$), 7.74 (d, *J* = 9.2 Hz, $2H_{ar}$); ^{13}C NMR (100 MHz, $CDCl_3$, 25 °C): δ = 13.2 (q), 55.5 (q), 77.2 (s), 81.2 (t), 89.9 (d), 114.1 (d), 120.6 (d), 130.6 (s), 157.2 (s), 160.4 (s), 171.4 (s), 207.6 (s); IR (nujol): ν_{max} = 3242, 1956, 1712, 1678 cm^{-1}; MS m/z (ESI): 259.12 (M + H^+); anal. calcd. for $C_{14}H_{14}N_2O_3$ (258.27): C 65.11, H 5.46, N 10.85; found: C 65.22, H 5.40, N 10.90.

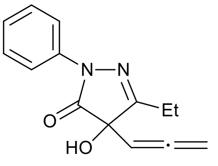

4d

3-Ethyl-4-hydroxy-1-phenyl-4-(propa-1,2-dienyl)-1*H*-pyrazol-5(4*H*)-one (4d): 4d was isolated by column chromatography (acetate/cyclohexane 20:80) and then by thin layer chromatography (three elutions in acetate/cyclohexane 20:80 mixture) in 18% yield. Orange solid; mp: 101–103; ^{1}H NMR (400 MHz, $CDCl_3$, 25 °C): δ = 1.32 (t, *J* = 7.2 Hz, 3H), 2.55–2.66 (m, 2H), 4.27–4.29 (m, 1H), 5.11 (d, *J* = 6.8 Hz, 2H), 5.37 (t, *J* = 6.4 Hz, 1H), 7.21 (t, *J* = 7.2 Hz, $2H_{ar}$), 7.41 (t, *J* = 7.6 Hz, $2H_{ar}$), 7.89 (d, *J* = 8. Hz, $1H_{ar}$); ^{13}C NMR (100 MHz, $CDCl_3$, 25 °C): δ = 9.3 (q), 21.0 (t), 78.1 (s), 81.1 (t), 90.1 (d), 118.8 (d), 125.3 (d), 128.8 (d), 137.7 (s), 164.6 (s), 172.4 (s), 207.5 (s); IR (nujol): ν_{max} = 3279, 1956, 1691 cm^{-1}; MS m/z (ESI): 243.12 (M + H^+); anal. calcd. for $C_{14}H_{14}N_2O_2$ (242.27): C 69.41, H 5.82, N 11.56; found: C 69.32, H 5.89, N 11.65.

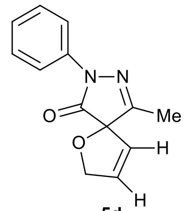
5d

9-Methyl-7-phenyl-1-oxa-7,8-diazaspiro[4.4]nona-3,8-dien-6-one (5d): 5d was isolated by column chromatography (acetate/cyclohexane 20:80) and then by thin layer chromatography (three elutions in acetate/cyclohexane 20:80 mixture) in 29% yield. Orange oil; ^{1}H NMR (400 MHz, $CDCl_3$, 25 °C): δ = 2.09 (s, 3H), 4.94–4.99 and 5.12–5.16 (2m, 2H), 5.62–5.65 (m, 1H), 6.46–6.48 (m, 1H), 7.19 (t, *J* = 7.6 Hz, 1H_{ar}), 7.40 (t, *J* = 7.6 Hz, 2H_{ar}), 7.89 (dd, *J* = 8.4 Hz, *J* =1.2, 2H_{ar}); ^{13}C NMR (100 MHz, $CDCl_3$, 25 °C): δ = 13.1 (q), 78.4 (t), 93.7 (s), 118.5 (d), 123.8 (d), 125.1 (d), 128.8 (d), 133.5 (d), 138.0 (s), 159.8 (s), 170.8 (s); IR (nujol): ν_{max} = 3314, 3081, 1762, 1597 cm^{-1} MS m/z (ESI): 227.20 (M - H$^+$); anal. calcd. for $C_{13}H_{12}N_2O_2$ (228.25): C 68.41, H 5.30, N 12.27; found: C 68.31, H 5.38, N 12.39.

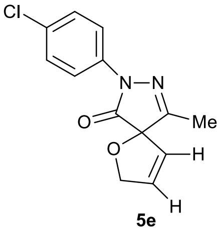
5e

7-(4-Chlorophenyl)-9-methyl-1-oxa-7,8-diazaspiro[4.4]nona-3,8-dien-6-one (5e): 5e was isolated by isolated by column chromatography (acetate/cyclohexane 20:80) and then by thin layer chromatography (three elutions in acetate/cyclohexane 20:80 mixture) in 17% yield. Orange solid; mp: 108–109 °C; ^{1}H NMR (400 MHz, $CDCl_3$, 25 °C): δ = 2.08 (s, 3H), 4.94–4.98 and 5.11–5.15 (2m, 2H), 5.62–5.64 (m, 1H), 6.47–6.49 (m, 1H), 7.36 (d, *J* = 9.2 Hz, 2H_{ar}), 7.86 (d, *J* = 9.2 Hz, 2H_{ar}); ^{13}C NMR (100 MHz, $CDCl_3$, 25 °C): δ = 13.1 (q), 78.4 (t), 93.6 (s), 119.6 (d), 123.6 (d), 128.9 (d), 130.1 (s), 133.7 (d), 136.6 (s), 160.1 (s), 170.7 (s); IR (nujol): ν_{max} = 3278, 3093, 1722, 1595 cm^{-1} MS m/z (ESI): 261.22 (M - H$^+$); anal. calcd. for $C_{13}H_{11}N_2O_2Cl$ (262.69): C 59.44, H 4.22, N 10.66; found: C 59.56, H 4.28, N 10.74.

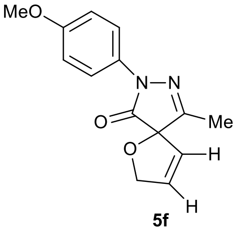
5f

7-(4-Methoxyphenyl)-9-methyl-1-oxa-7,8-diazaspiro[4.4]nona-3,8-dien-6-one (5f): 5f was isolated by isolated by column chromatography (acetate/cyclohexane 20:80) and then by thin layer chromatography (three elutions in acetate/cyclohexane 20:80 mixture) in 21% yield. Brown oil; ^{1}H NMR (400 MHz, $CDCl_3$, 25 °C): δ = 2.08 (s, 3H), 3.82 (s, 3H), 4.94–4.98 and 5.12–5.15 (2m, 2H), 5.62–5.65 (m, 1H), 6.46–6.48 (m, 1H), 6.93 (d, *J* = 9.2 Hz, 2H_{ar}), 7.77 (d, *J* = 9.2 Hz, 2H_{ar}); ^{13}C NMR (100 MHz, $CDCl_3$, 25 °C): δ = 13.1 (q), 55.5 (q), 78.4 (t), 93.6 (s), 114.0 (d), 120.4 (d), 123.8 (d), 131.4 (s), 133.4 (d), 157.0 (s), 159.7 (s), 170.4 (s); IR (nujol): ν_{max} = 3279, 3086, 1731, 1717, 1610 cm^{-1} MS m/z (ESI): 257.13 (M - H$^+$); anal. calcd. for $C_{14}H_{14}N_2O_3$ (258.27): C 65.11, H 5.46, N 10.85; found: C 64.99, H 5.54, N 10.76.

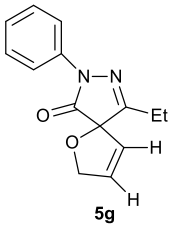
5g

9-Ethyl-7-phenyl-1-oxa-7,8-diazaspiro[4.4]nona-3,8-dien-6-one (5g): 5g was isolated by isolated by column chromatography (acetate/cyclohexane 20:80) and then by thin layer chromatography (three elutions in acetate/cyclohexane 20:80 mixture) in 18% yield. Yellow solid; mp: 121-123 °C; ^{1}H NMR (400 MHz, $CDCl_3$, 25 °C): δ = 1.27 (t, *J* = 7.2 Hz, 3H), 2.34–2.54 (m, 2H), 4.93–4.97 and 5.11–5.15

(2m, 2H), 5.63–5.66 (m, 1H), 6.44–6.46 (m, 1H), 7.19 (t, *J* = 7.6 Hz, $1H_{ar}$), 7.41 (t, *J* = 7.6 Hz, $2H_{ar}$), 7.92 (dd, *J* = 8.8 Hz, *J* = 1.2, $2H_{ar}$); ^{13}C NMR (100 MHz, $CDCl_3$, 25 °C): δ = 9.7 (q), 21.1 (t), 78.2 (t), 93.7 (s), 118.5 (d), 124.1 (d), 125.0 (d), 128.8 (d), 133.1 (d), 138.0 (s), 163.5 (s), 170.9 (s); IR (nujol): ν_{max} = 3096, 1714, 1632, 1597 cm^{-1} MS m/z (ESI): 241.25 (M - H^+); anal. calcd. for $C_{14}H_{14}N_2O_2$ (242.27): C 69.41, H 5.82, N 11.56; found: C 69.31, H 5.77, N 11.62.

General procedure for the synthesis of *tert*-butyl 2-(3-(allyloxy)-4-ethoxy-4-oxobutan-2-ylidene)hydrazinecarboxylate (3d) or *tert*-butyl 2-(3-(but-2-en-1-yloxy)-4-ethoxy-4-oxobutan-2-ylidene)hydrazinecarboxylate (3e), starting from 1,2-diaza-1,3-diene 1h and allyl (2b) or crotyl (2c) alcohol.

To a magnetically stirred mixture of 1,2-diaza-1,3-diene (1 mmol) and allyl (**2b**) or crotyl (**2c**) alcohol (10 equiv.) at room temperature, K_2CO_3 (1 mmol) was added and the suspension was stand under these conditions for 0.1 h, until the disappearance of the reagent **1** (TLC monitoring). The crude mixture was quickly filtered and then purified by column chromatography on silica gel to afford compounds **3d,e**, that were crystallized from EtOAc.

Characterization data of products 3d,e.

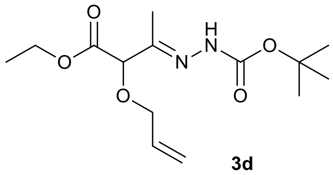

3d

***Tert*-butyl 2-(3-(allyloxy)-4-ethoxy-4-oxobutan-2-ylidene)hydrazinecarboxylate (3d): 3d** was isolated by column chromatography (acetate/cyclohexane 40:60) in 37% yield. White solid; mp: 119-120 °C; ^{1}H NMR (400 MHz, $CDCl_3$, 25 °C): δ = 1.27 (t, *J* = 7.2 Hz, 3H), 1.52 (s, 9H), 1.83 (s, 3H), 4.02–4.12 (m, 2H), 4.20–4.28 (m, 2H), 4.63 (s, 1H), 5.21 (d, *J* = 10.0 Hz, 1H), 5.28–5.35 (m, 1H), 5.86–5.95 (m, 1H), 7.60 (s, 1H); ^{13}C NMR (100 MHz, $CDCl_3$, 25 °C): δ = 10.8 (q), 14.1 (q), 28.2 (q), 61.4 (t), 70.8 (t), 81.6 (s), 81.8 (d), 118.2 (t), 133.5 (d), 146.8 (s), 152.3 (s), 169.1 (s); IR (nujol): ν_{max} = 3235, 1760, 1725, 1704 cm^{-1}; MS m/z (ESI): 303.22 (M + H^+); anal. calcd. for $C_{14}H_{24}N_2O_5$ (302.17): C 55.98, H 8.05, N 9.33; found: C 56.16, H 8.40, N 9.18.

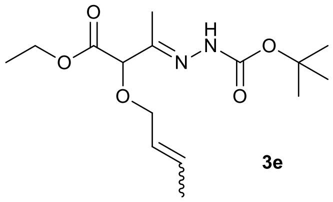

3e

***Tert*-butyl 2-(3-(but-2-en-1-yloxy)-4-ethoxy-4-oxobutan-2-ylidene)hydrazinecarboxylate (3e): 3e** was isolated by column chromatography (acetate/cyclohexane 40:60) in 22% yield. White solid; mp: 158-160 °C; ^{1}H NMR (400 MHz, $CDCl_3$, 25 °C): δ = 1.27 (t, *J* = 7.2 Hz, 3H), 1.52 (s, 9H), 1.71 (dd, *J* = 6.4 Hz, *J* = 0.8 Hz, 3H), 1.82 (s, 3H), 3.96–4.01 (m, 2H), 4.22 (q, *J* = 7.2 Hz, 2H), 4.62 (s, 1H), 5.53–5.61 (m, 1H), 5.70–5.79 (m, 1H), 7.57 (s, 1H); ^{13}C NMR (100 MHz, $CDCl_3$, 25 °C): δ = 10.8 (q), 14.2 (q), 17.8 (q), 28.2 (q), 61.4 (t), 70.6 (t), 81.4 (d), 81.5 (s), 126.3 (d), 131.1 (d), 146.9 (s), 152.2 (s), 169.3 (s); IR (nujol): ν_{max} = 3372, 3269,

1715, 1627 cm^{-1}; MS m/z (ESI): 313.09 (M - H$^+$); anal. calcd. for $C_{15}H_{26}N_2O_5$ (314.18): C 57.31, H 8.34, N 8.91; found: C 57.16, H 8.41, N 8.98.

General procedure for the synthesis of 4-allyl-4-hydroxy-3-alkyl-1-aryl-1*H*-pyrazol-5(4*H*)-ones 6a–d, starting from 1,2-diaza-1,3-dienes 1g–j and allyl alcohol 2b.

To a magnetically stirred mixture of 1,2-diaza-1,3-dienes **1g–j** (1 mmol) and allyl alcohol **2b** (3 mL) at 60 °C (oil bath), K_2CO_3 (4 mmol) was added and the suspension was stand under these conditions for the appropriate time (8.0–16.0 hours), until the disappearance of the reagent **1** (TLC monitoring). The crude mixture was then purified by column chromatography on silica gel to afford 4-allyl-4-hydroxy-3-alkyl-1-aryl-1*H*-pyrazol-5(4*H*)-ones **6a–d** as oils, in the case of **6a,c,d** or solid that was crystallized from EtOAc-light petroleum ether in the case of **6b**.

General procedure for the synthesis of 4-allyl-4-hydroxy-3-methyl-1-alcoxycarbonyl-1*H*-pyrazol-5(4*H*)-one 6e, starting from 3d.

To a magnetically stirred solution of *tert*-butyl 2-(3-(allyloxy)-4-ethoxy-4-oxobutan-2-ylidene)hydrazinecarboxylate **3d** (1 mmol) in ethanol (3 mL), K_2CO_3 (1 mmol) was added and the suspension was stand under these conditions for 2.0 hours, until the disappearance of the reagent **3** (TLC monitoring). The crude mixture was then filtered and purified by column chromatography on silica gel to afford **6e** as oil.

Characterization data of products 6a–e.

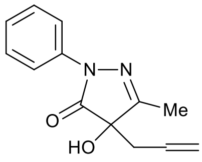

6a

4-Allyl-4-hydroxy-3-methyl-1-phenyl-1*H*-pyrazol-5(4*H*)-one (6a): 6a was isolated by column chromatography (acetate/cyclohexane 20:80) in 25% yield. Brown oil; ^{1}H NMR (400 MHz, $CDCl_3$, 25 °C): δ = 2.18 (s, 3H), 2.59–2.73 (m, 2H), 3.90 (brs, 1H), 5.15–5.24 (m, 2H), 5.55–5.65 (m, 1H), 7.20 (t, *J* = 7.6 Hz, $1H_{ar}$), 7.39 (t, *J* = 8.8 Hz, $2H_{ar}$), 7.83 (d, *J* = 7.6 Hz, $2H_{ar}$); ^{13}C NMR (100 MHz, $CDCl_3$, 25 °C): δ = 13.2 (q), 40.6 (t), 79.6 (s), 118.9 (d), 121.4 (t), 125.4 (d), 128.5 (d), 128.8 (d), 137.4 (s), 161.5 (s), 173.4 (s); IR (nujol): ν_{max} = 3263, 1683, 1596 cm^{-1}; MS m/z (ESI): 231.30 (M + H$^+$); anal. calcd. for $C_{13}H_{14}N_2O_2$ (230.26): C 67.81, H 6.13, N 12.17; found: C 67.70, H 6.19, N 12.22.

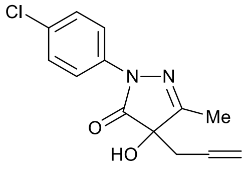

6b

4-Allyl-1-(4-chlorophenyl)-4-hydroxy-3-methyl-1*H*-pyrazol-5(4*H*)-one (6b): 6b was isolated by column chromatography (acetate/cyclohexane 20:80) in 33% yield and crystallized from EtOAc-light petroleum ether. Yellow solid; mp: 131–133 °C; ^{1}H NMR (400 MHz, $CDCl_3$, 25 °C): δ = 2.18 (s, 3H), 2.58–2.71 (m, 2H), 4.19 (brs, 1H), 5.15–5.24 (m, 2H), 5.52–5.62 (m, 1H), 7.33 (d, *J* = 8.8 Hz, $2H_{ar}$), 7.79 (d, *J* = 8.8 Hz, $2H_{ar}$); ^{13}C NMR (100 MHz, $CDCl_3$, 25 °C): δ = 13.2 (q), 40.5 (t), 79.6 (s), 119.9 (d), 121.5 (t), 128.3 (d), 128.9 (d), 130.5 (s), 135.9 (s), 161.9 (s), 173.5 (s); IR (nujol): ν_{max} = 3302, 1685, 1625

cm^{-1}; MS m/z (ESI): 265.09 (M + H$^+$); anal. calcd. for $C_{13}H_{13}N_2O_2Cl$ (264.71): C 58.99, H 4.95, N 10.58; found: C 59.11, H 4.89, N 10.66.

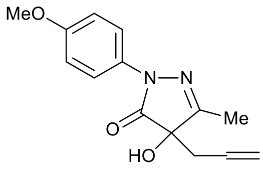

6c

4-Allyl-4-hydroxy-1-(4-methoxyphenyl)-3-methyl-1*H*-pyrazol-5(4*H*)-one (6c): 6c was isolated by column chromatography (acetate/cyclohexane 20:80) in 33% yield. Brown oil; ^{1}H NMR (400 MHz, $CDCl_3$, 25 °C): δ = 2.17 (s, 3H), 2.60–2.64 (m, 2H), 3.82 (s, 3H), 4.10 (brs, 1H), 5.20–5.27 (m, 2H), 5.60–5.69 (m, 1H), 6.93 (d, J = 9.2 Hz, $2H_{ar}$), 7.72 (d, J = 9.2 Hz, $2H_{ar}$); ^{13}C NMR (100 MHz, $CDCl_3$, 25 °C): δ = 13.3 (q), 40.7 (t), 55.5 (q), 79.0 (s), 114.0 (d), 120.7 (d), 121.5 (t), 128.5 (d), 130.8 (s), 157.2 (s), 160.8 (s), 172.5 (s); IR (nujol): ν_{max} = 3357, 1693, 1608 cm^{-1}; MS m/z (ESI): 259.23 (M - H$^+$); anal. calcd. for $C_{14}H_{16}N_2O_3$ (260.29): C 64.60, H 6.20, N 10.76; found: C 64.51, H 6.24, N 10.67.

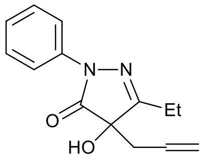

6d

4-Allyl-3-ethyl-4-hydroxy-1-phenyl-1*H*-pyrazol-5(4*H*)-one (6d): 6d was isolated by column chromatography (acetate/cyclohexane 20:80) in 28% yield. Red oil; ^{1}H NMR (400 MHz, $CDCl_3$, 25 °C): δ = 1.32 (t, J = 7.2 Hz, 3H), 2.44–2.70 (m, 4H), 3.65 (brs, 1H), 5.16–5.25 (m, 2H), 5.56–5.66 (m, 1H), 7.20 (t, J = 7.2 Hz, $1H_{ar}$), 7.40 (t, J = 8.4 Hz, $2H_{ar}$), 7.87 (dd, J = 8.8 Hz, J = 1.2, $2H_{ar}$); ^{13}C NMR (100 MHz, $CDCl_3$, 25 °C): δ = 9.1 (q), 21.0 (t), 40.9 (t), 79.7 (s), 118.8 (d), 121.4 (t), 125.2 (d), 128.6 (d), 128.8 (d), 137.6 (s), 164.9 (s), 173.5 (s); IR (nujol): ν_{max} = 3313, 1689, 1625, 1597 cm^{-1}; MS *m/z* (%):MS m/z (ESI): 245.14 (M + H$^+$); anal. calcd. for $C_{14}H_{16}N_2O_2$ (244.29): C 67.83, H 6.60, N 11.47; found: C 67.71, H 6.65, N 11.54.

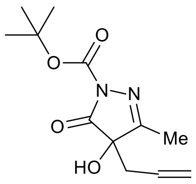

6e

***Tert*-butyl 4-allyl-4-hydroxy-3-methyl-5-oxo-4,5-dihydro-1*H*-pyrazole-1-carboxylate (6e): 6e** was isolated by column chromatography (acetate/cyclohexane 20:80) in 70% yield. Pale yellow oil; ^{1}H NMR (400 MHz, $CDCl_3$, 25 °C): δ = 1.59 (s, 9H), 2.13 (s, 3H), 2.55 (d, J = 7.2 Hz, 2H), 3.10 (brs, 1H), 5.20–5.30 (m, 2H), 5.61–5.71 (m, 1H); ^{13}C NMR (100 MHz, $CDCl_3$, 25 °C): δ = 13.4 (q), 28.0 (q), 40.3 (t), 78.1 (s), 84.9 (s), 122.2 (t), 127.9 (d), 147.5 (s), 161.5 (s), 173.2 (s); IR (nujol): ν_{max} = 3268, 1678, 1619 cm-1; MS m/z (ESI): 253.22 (M - H$^+$); anal. calcd. for $C_{12}H_{18}N_2O_4$ (254.28): C 56.68, H 7.13, N 11.02; found: C 56.84, H 7.02, N 10.86.

3. Table S1.

Table S1 Screening of different conditions tested in the reaction between DDs **1d–g** and crotyl alcohol **2c**.[a]

entry	Solvent	Catalyst	Equivalents	T	Trend of the reaction
1	CH_2Cl_2	DBU	0.1	rt	no reaction
2	CH_2Cl_2	DBU	0.5	rt	no reaction
3	CH_2Cl_2	DBU	0.5	60 °C	complicated mixture
4	CH_2Cl_2	DBU	1	rt	complicated mixture
5	MeCN	DBU	0.1	rt	no reaction
6	Me CN	DBU	0.1	60 °C	complicated mixture
7	MeCN	DBU	1	rt	complicated mixture
8	THF	DBU	0.1	rt	no reaction
9	THF	DBU	0.1	60 °C	complicated mixture
10	THF	DBU	1	rt	complicated mixture
11	MeOH	DBU	0.1	rt	no reaction
12	MeOH	DBU	0.5	rt	no reaction
13	MeOH	DBU	0.5	60 °C	complicated mixture
14	MeOH	DBU	1	rt	complicated mixture
15	**2c** as s/r[b]	DBU	1	rt	complicated mixture[c]
16	MeCN	DIPEA	2	rt	no reaction
17	MeCN	DIPEA	4	rt	no reaction
18	THF	DIPEA	4	rt	no reaction
19	CH_2Cl_2	DIPEA	4	rt	no reaction
20	**2c** as s/r[b]	DIPEA	4	rt	no reaction
21	**2c** as s/r[b]	DIPEA	4	60 °C	no reaction
22	MeCN	NaH	0.1	rt	complicated mixture
23	THF	NaH	0.1	rt	complicated mixture
24	CH_2Cl_2	NaH	0.1	rt	complicated mixture
25	**2c** as s/r[b]	NaH	0.1	rt	complicated mixture
26	MeCN	MeONa	0.1	rt	complicated mixture
27	THF	MeONa	0.1	rt	complicated mixture
28	CH_2Cl_2	MeONa	0.1	rt	complicated mixture
29	**2c** as s/r[b]	MeONa	0.1	rt	complicated mixture
30	MeCN	K_2CO_3	4	rt	no reaction
31	THF	K_2CO_3	4	rt	no reaction
32	CH_2Cl_2	K_2CO_3	4	rt	no reaction
33	**2c** as s/r[b]	K_2CO_3	4	rt	no reaction
34	**2c** as s/r[b]	K_2CO_3	4	60 °C	complicated mixture

[a]The reactions were performed at 0.5 mmol scale of DDs **1** in 3 mL of solvent,. 1.2. equiv. of **2c** was used. [b]**2c** was employed as solvent/reagent (3 mL).

4. Table S2.

Table S2 Screening of different conditions tested to tentatively convert **3e** into the corresponding pyrazol-5(4*H*)-one.[a]

entry	Solvent	Catalyst	Equivalents	T	Trend of the reaction
1	CH_2Cl_2	DBU	1	rt	no reaction
2	CH_2Cl_2	DBU	1	60 °C	no reaction
3	$CHCl_3$	DBU	1	rt	no reaction
4	$CHCl_3$	DBU	1	60 °C	no reaction
5	$CHCl_3$	DBU	2	60 °C	no reaction
6	MeCN	DBU	1	rt	no reaction
7	Me CN	DBU	1	60 °C	no reaction
8	MeCN	DBU	3	rt	complicated mixture
9	THF	DBU	0.1	rt	no reaction
10	THF	DBU	0.1	60 °C	no reaction
11	THF	DBU	1	rt	no reaction
12	THF	DBU	1	60 °C	no reaction
13	THF	DBU	4	rt	complicated mixture
14	MeOH	DBU	0.1	rt	no reaction
15	MeOH	DBU	0.5	rt	no reaction
16	MeOH	DBU	1	rt	no reaction
17	MeOH	DBU	1	60 °C	complicated mixture
18	$CHCl_3$	$Zn(OTf)_3$	0.2	rt	no reaction
19	$CHCl_3$	$Zn(OTf)_3$	1	rt	no reaction
20	$CHCl_3$	$Zn(OTf)_3$	1	rt	no reaction
21	MeCN	NaH	0.1	rt	no reaction
22	MeCN	NaH	0.1	60 °C	complicated mixture
23	MeCN	NaH	1	rt	complicated mixture
24	THF	NaH	0.1	rt	no reaction
25	THF	NaH	0.1	60 °C	complicated mixture
26	THF	NaH	1	rt	complicated mixture
27	CH_2Cl_2	NaH	0.1	rt	no reaction
28	CH_2Cl_2	NaH	0.1	60 °C	complicated mixture
29	CH_2Cl_2	NaH	1	rt	complicated mixture
30	MeOH	NaH	0.1	rt	complicated mixture
31	MeCN	MeONa	0.1	rt	no reaction
32	MeCN	MeONa	0.1	60 °C	complicated mixture
33	MeCN	MeONa	1	rt	complicated mixture
34	THF	MeONa	0.1	rt	no reaction
35	THF	MeONa	0.1	60 °C	complicated mixture
36	THF	MeONa	1	60 °C	complicated mixture
37	CH_2Cl_2	MeONa	1	rt	no reaction

38	CH_2Cl_2	MeONa	1	60 °C	complicated mixture
39	MeCN	K_2CO_3	4	rt	no reaction
40	THF	K_2CO_3	4	rt	no reaction
41	CH_2Cl_2	K_2CO_3	4	rt	no reaction
42	MeOH	K_2CO_3	4	rt	no reaction
43	EtOH	K_2CO_3	1	rt	no reaction
44	EtOH	K_2CO_3	4	rt	no reaction
45	EtOH	K_2CO_3	4	reflux	no reaction

[a]The reactions were performed at 0.5 mmol scale of **3e** in 3 mL of solvent.

5. ^{1}H and ^{13}C NMR spectra of products.

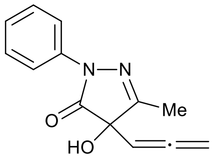

4a

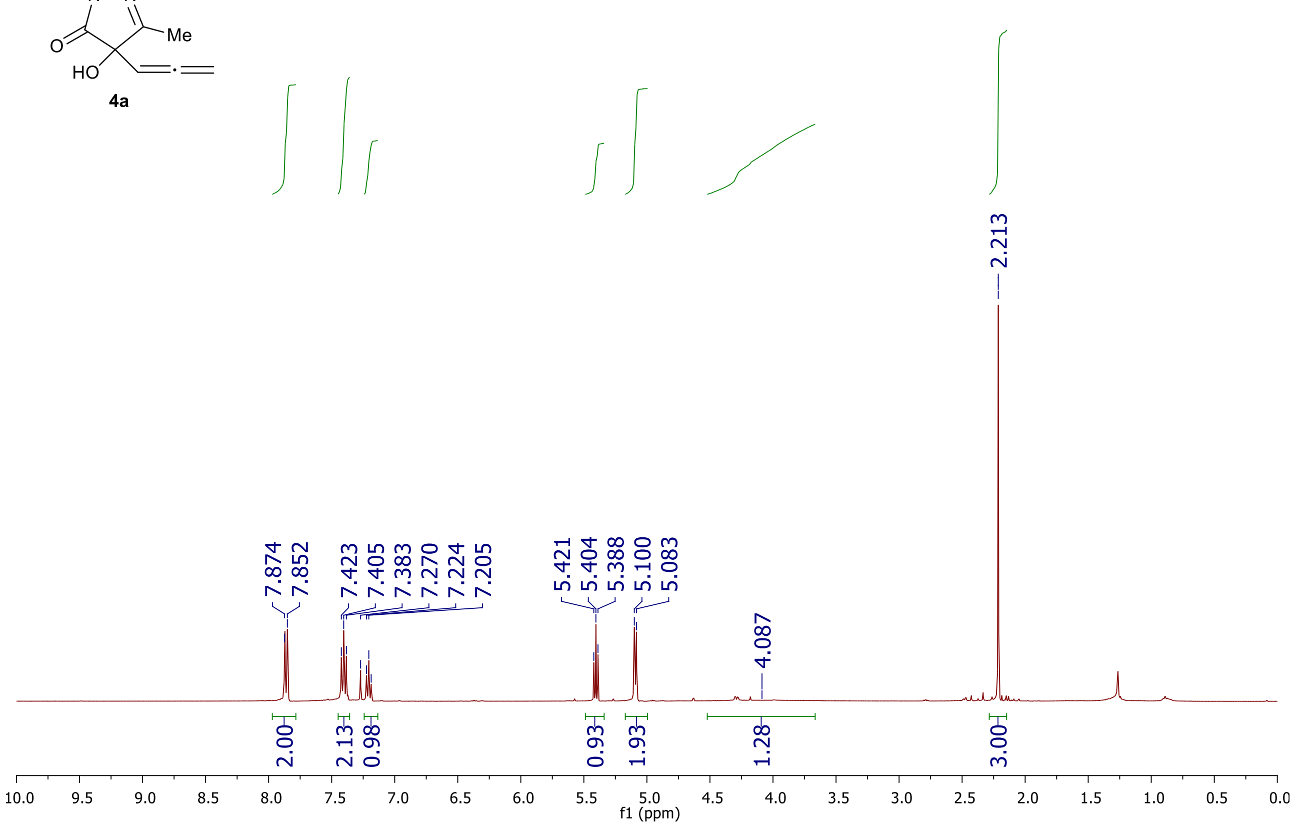

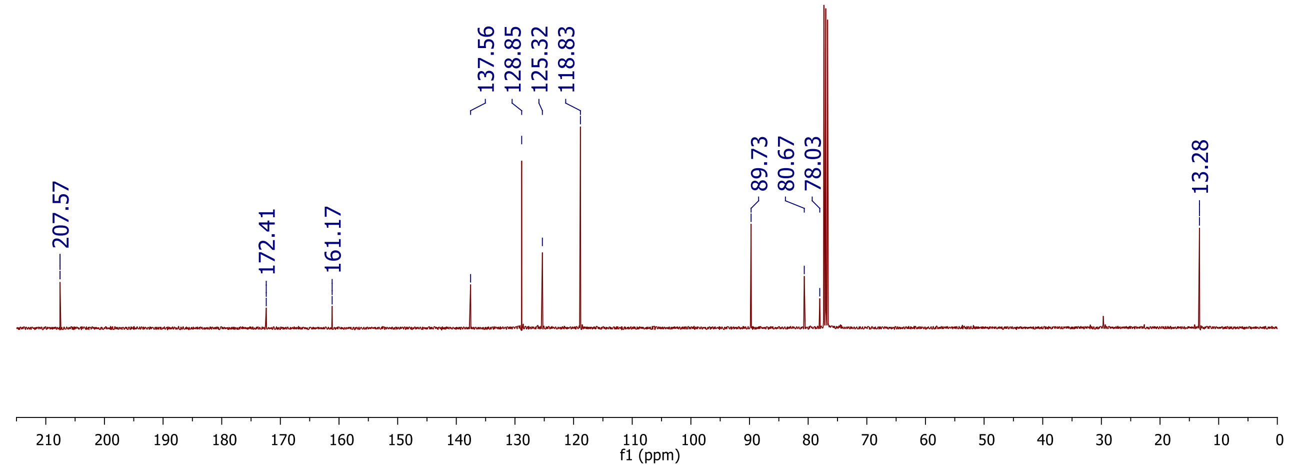

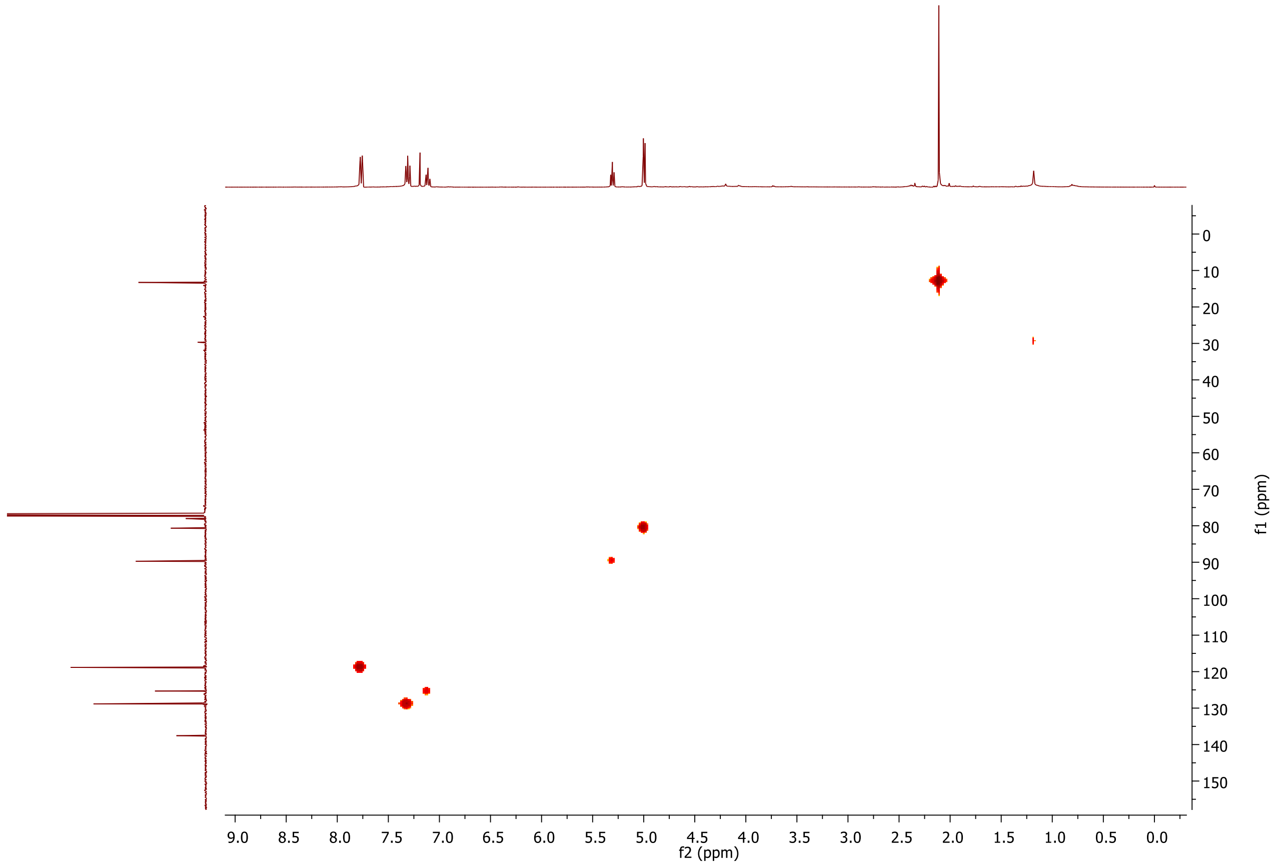

9.0
8.5
8.0
7.5
7.0
6.5
6.0
5.5
5.0
4.5
4.0
3.5
3.0
2.5
2.0
1.5
1.0
0.5
0.0
f2 (ppm)
0
10
20
30
40
50
60
70
80
90
100
110
120
130
140
150
f1 (ppm)

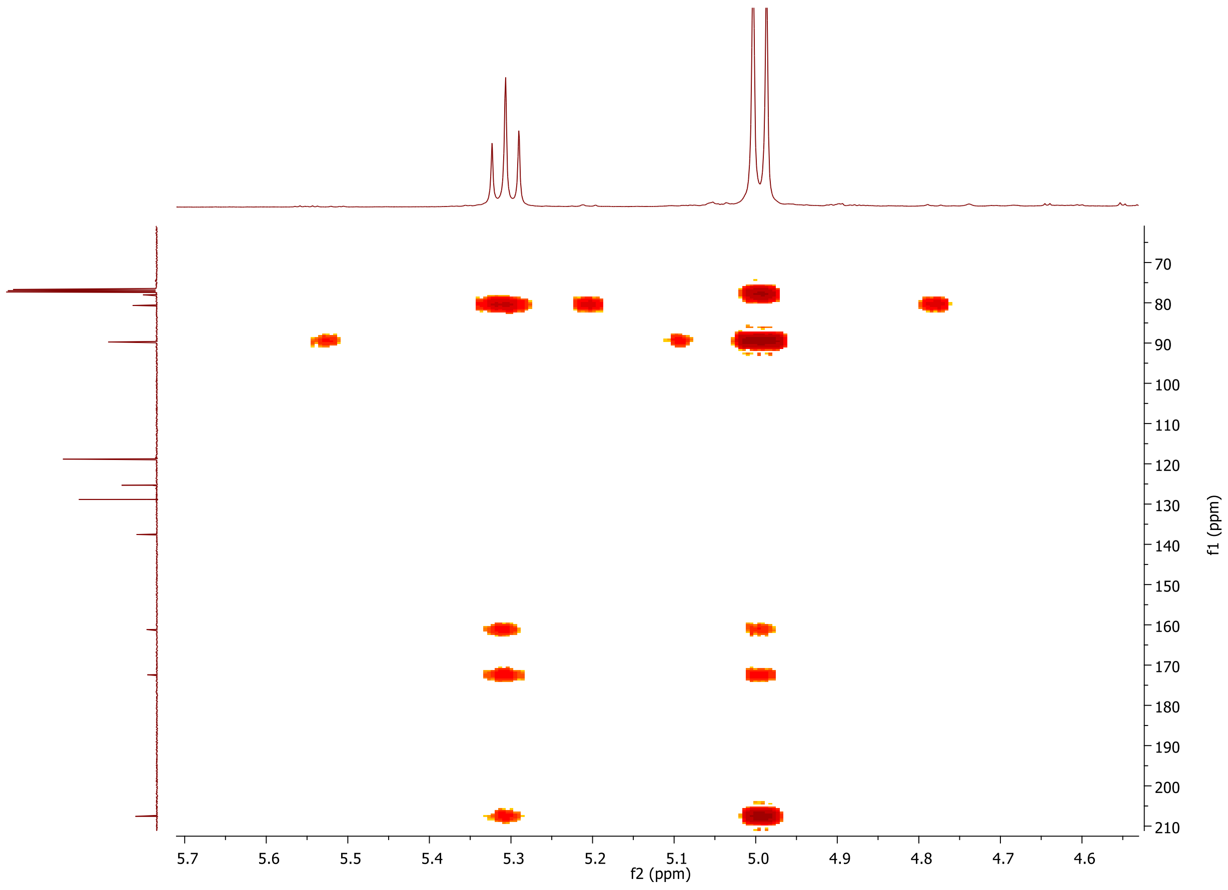

5.7
5.6
5.5
5.4
5.3
5.2
5.1
5.0
4.9
4.8
4.7
4.6
f2 (ppm)
70
80
90
100
110
120
130
140
150
160
170
180
190
200
210
f1 (ppm)

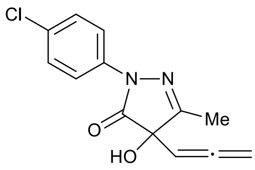
Cl
N
N
Me
O
HO

4b

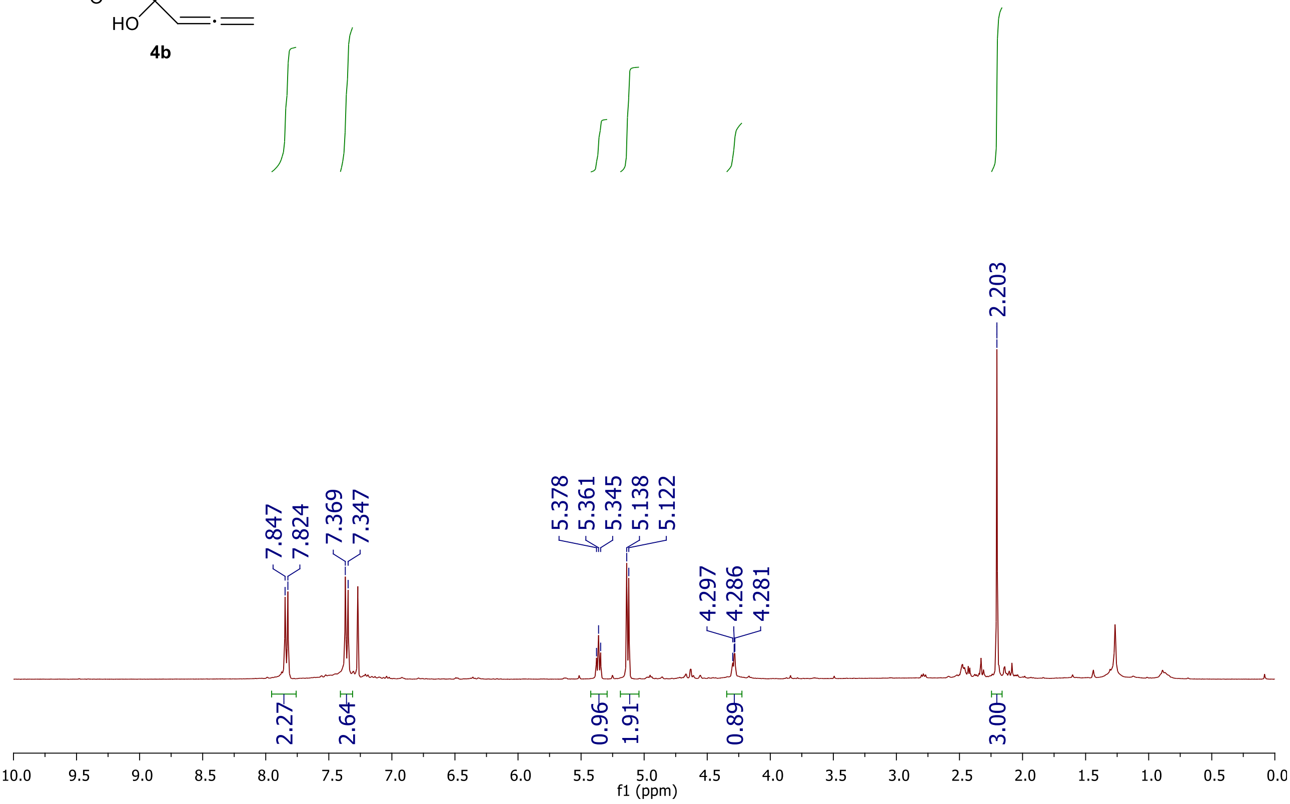
7.847
7.824
7.369
7.347
5.378
5.361
5.345
5.138
5.122
4.297
4.286
4.281
2.203
2.27
2.64
0.96
1.91
0.89
3.00
10.0 9.5 9.0 8.5 8.0 7.5 7.0 6.5 6.0 5.5 5.0 4.5 4.0 3.5 3.0 2.5 2.0 1.5 1.0 0.5 0.0
f1 (ppm)

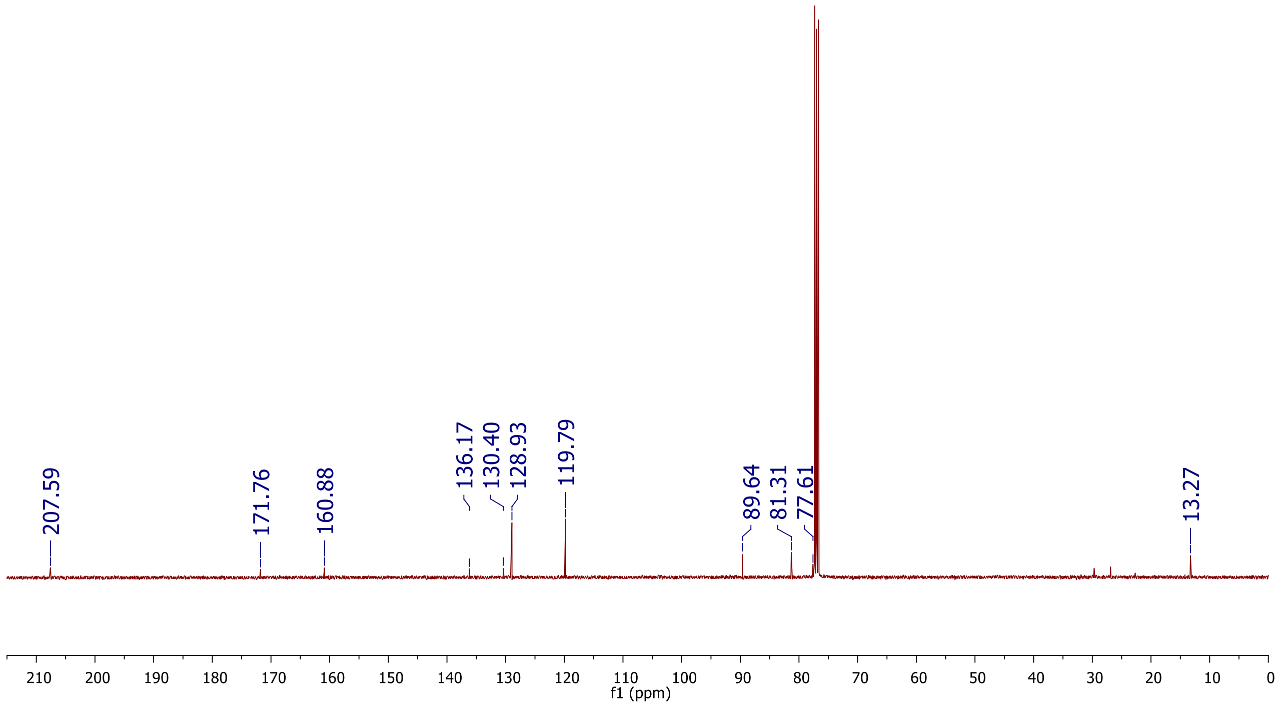
207.59
171.76
160.88
136.17
130.40
128.93
119.79
89.64
81.31
77.61
13.27
210 200 190 180 170 160 150 140 130 120 110 100 90 80 70 60 50 40 30 20 10 0
f1 (ppm)

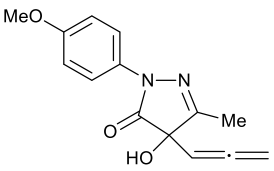
MeO
N
N
Me
O
HO

4c

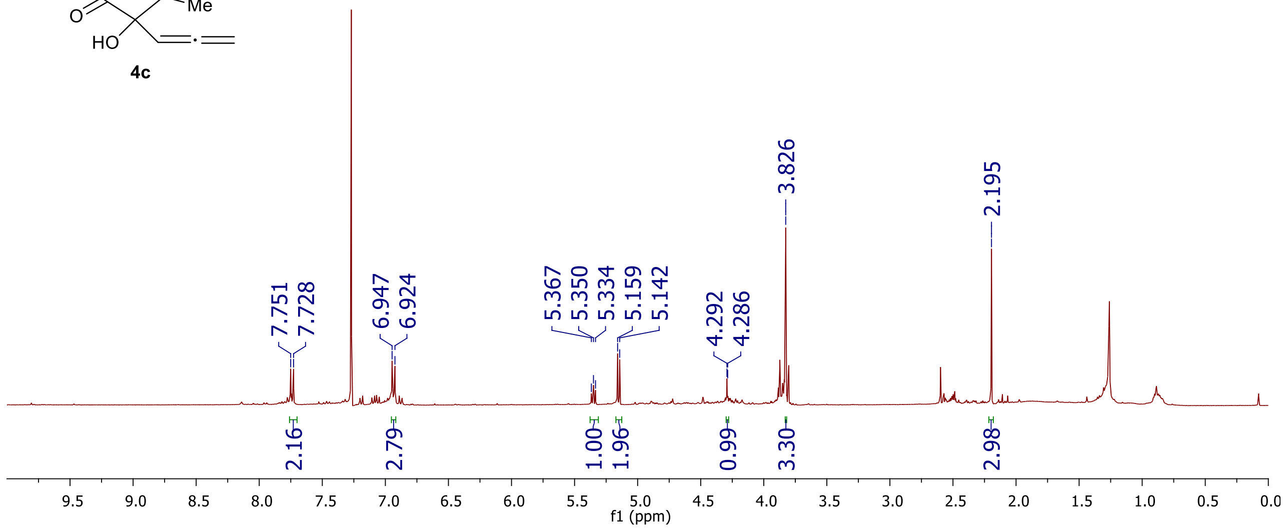
7.751
7.728
6.947
6.924
5.367
5.350
5.334
5.159
5.142
4.292
4.286
3.826
2.195
2.16
2.79
1.00
1.96
0.99
3.30
2.98
9.5 9.0 8.5 8.0 7.5 7.0 6.5 6.0 5.5 5.0 4.5 4.0 3.5 3.0 2.5 2.0 1.5 1.0 0.5 0.0
f1 (ppm)

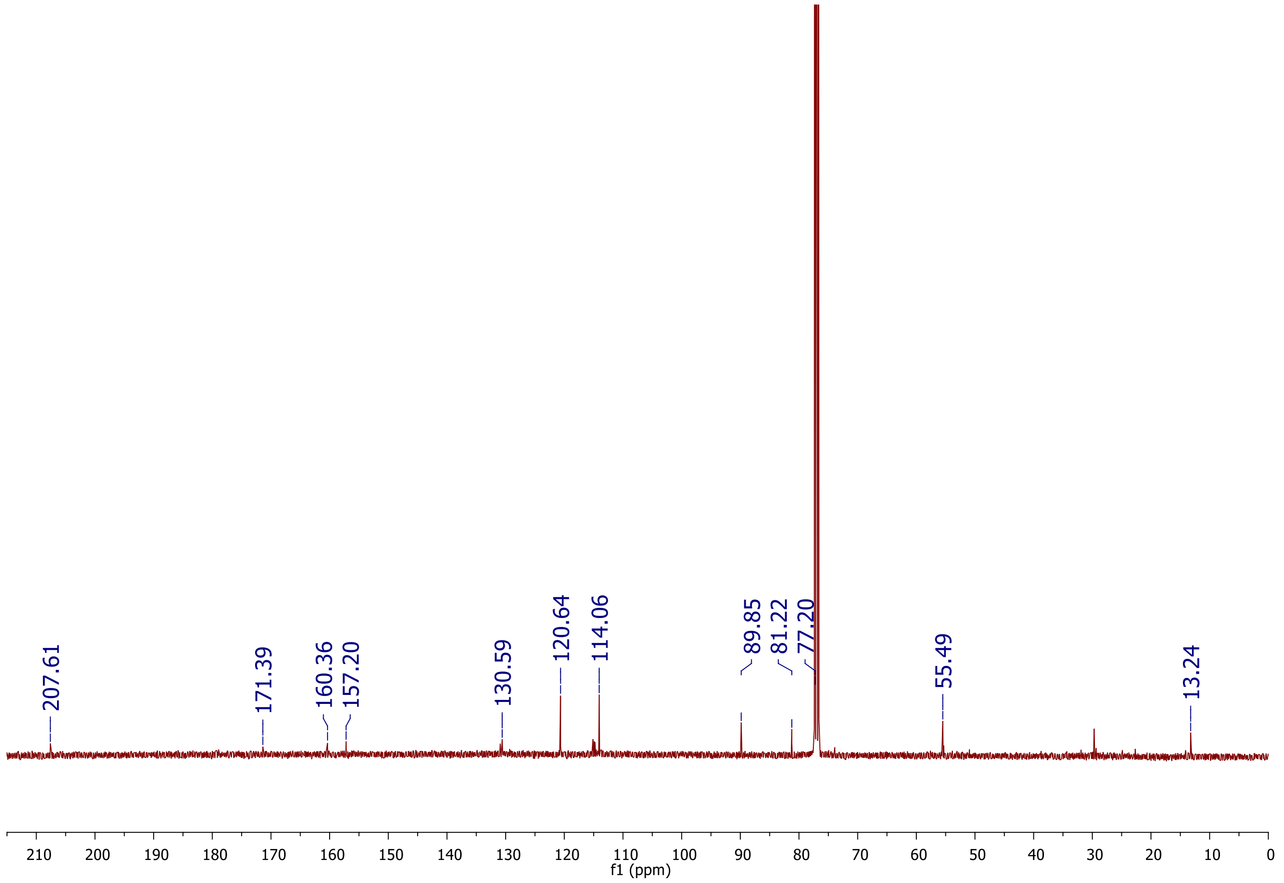
207.61
171.39
160.36
157.20
130.59
120.64
114.06
89.85
81.22
77.20
55.49
13.24
210 200 190 180 170 160 150 140 130 120 110 100 90 80 70 60 50 40 30 20 10 0
f1 (ppm)

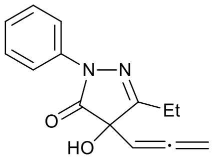
N
N
Et
O
HO

4d

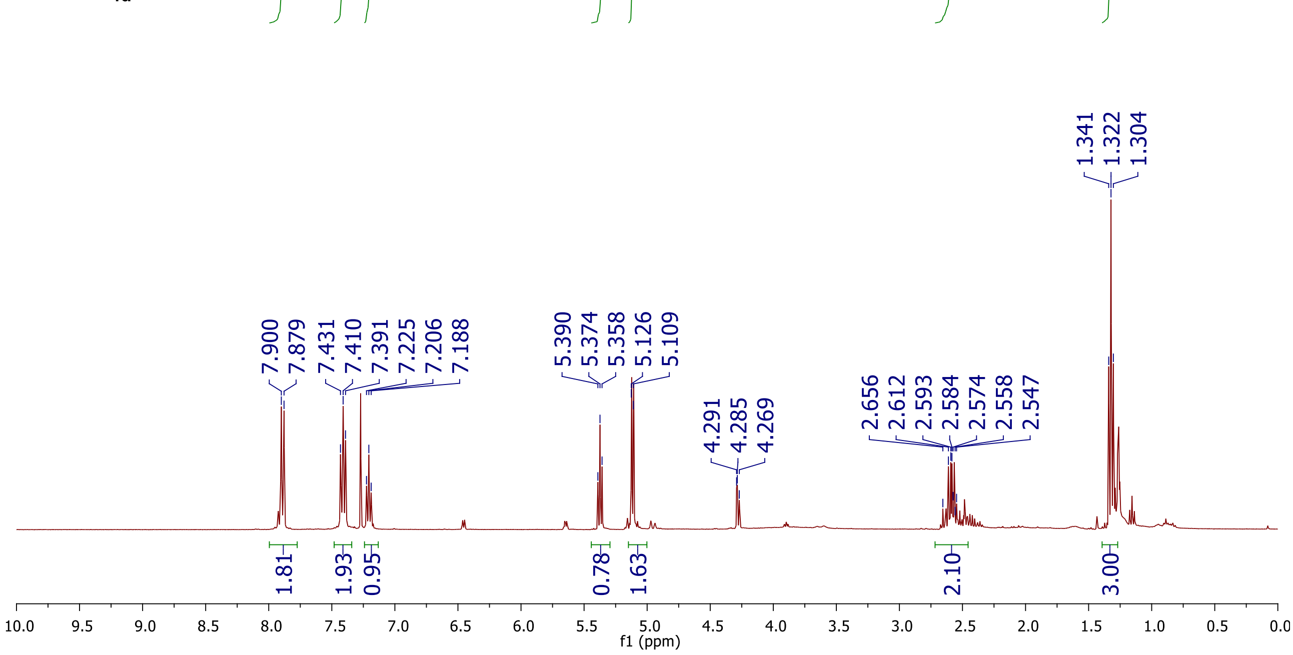
7.900
7.879
7.431
7.410
7.391
7.225
7.206
7.188
5.390
5.374
5.358
5.126
5.109
4.291
4.285
4.269
2.656
2.612
2.593
2.584
2.574
2.558
2.547
1.341
1.322
1.304
1.81
1.93
0.95
0.78
1.63
2.10
3.00
f1 (ppm)

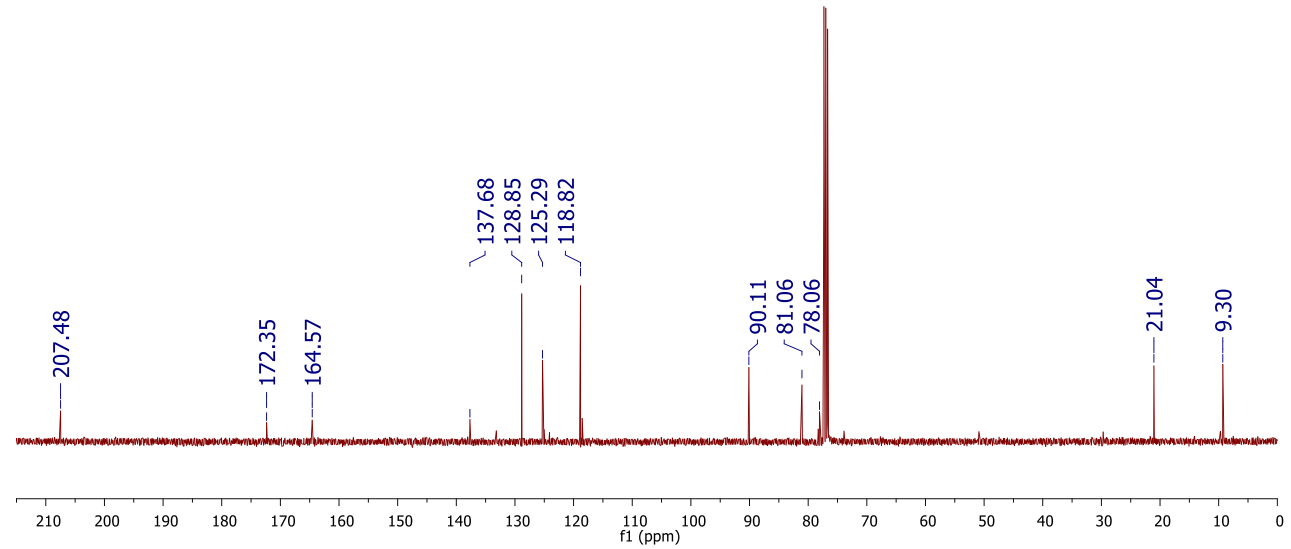
207.48
172.35
164.57
137.68
128.85
125.29
118.82
90.11
81.06
78.06
21.04
9.30
f1 (ppm)

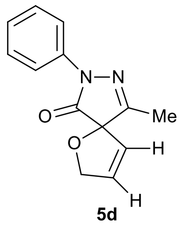
N
N
Me
O
O
H
H

5d

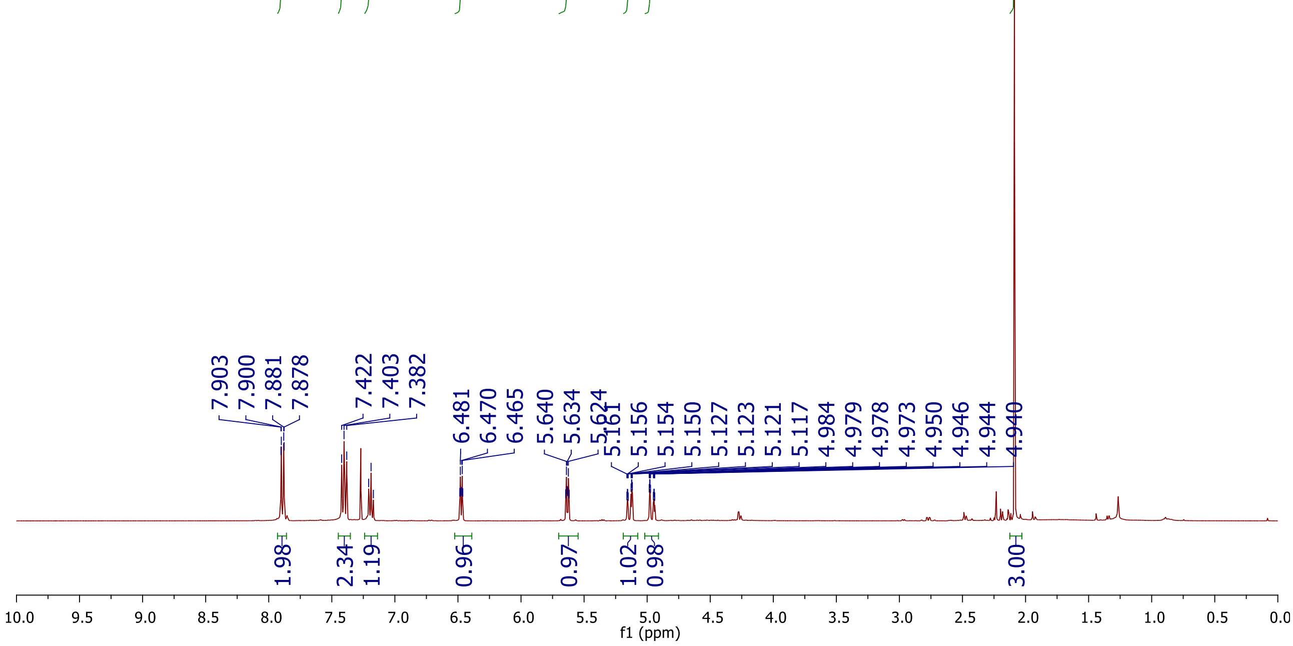
7.903
7.900
7.881
7.878
7.422
7.403
7.382
6.481
6.470
6.465
5.640
5.634
5.624
5.161
5.156
5.154
5.150
5.127
5.123
5.121
5.117
4.984
4.979
4.978
4.973
4.950
4.946
4.944
4.940
2.088
1.98
2.34
1.19
0.96
0.97
1.02
0.98
3.00
10.0 9.5 9.0 8.5 8.0 7.5 7.0 6.5 6.0 5.5 5.0 4.5 4.0 3.5 3.0 2.5 2.0 1.5 1.0 0.5 0.0
f1 (ppm)

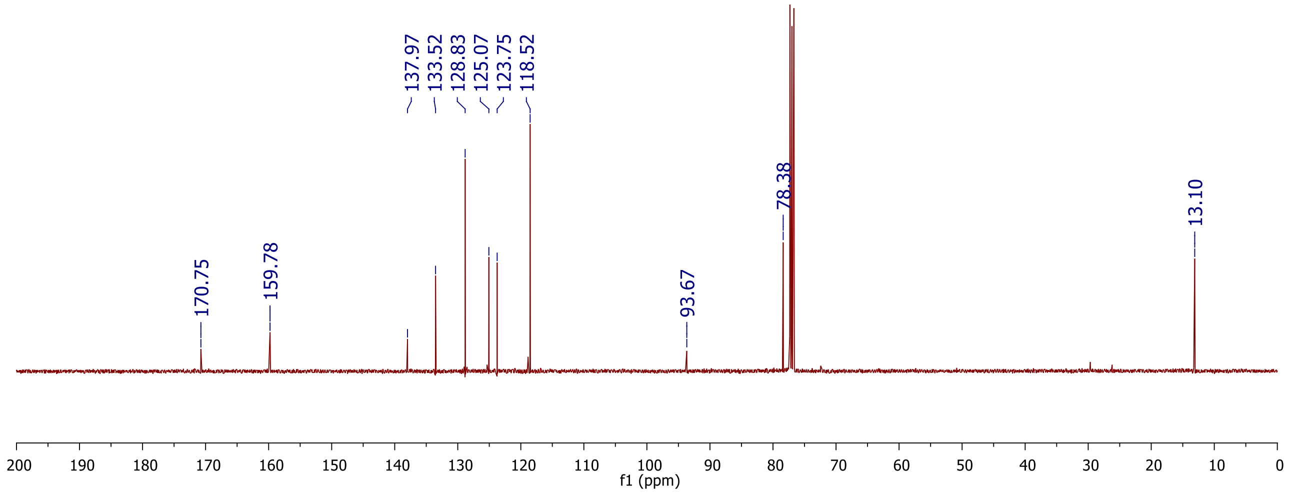
137.97
133.52
128.83
125.07
123.75
118.52
170.75
159.78
93.67
78.38
13.10
200 190 180 170 160 150 140 130 120 110 100 90 80 70 60 50 40 30 20 10 0
f1 (ppm)

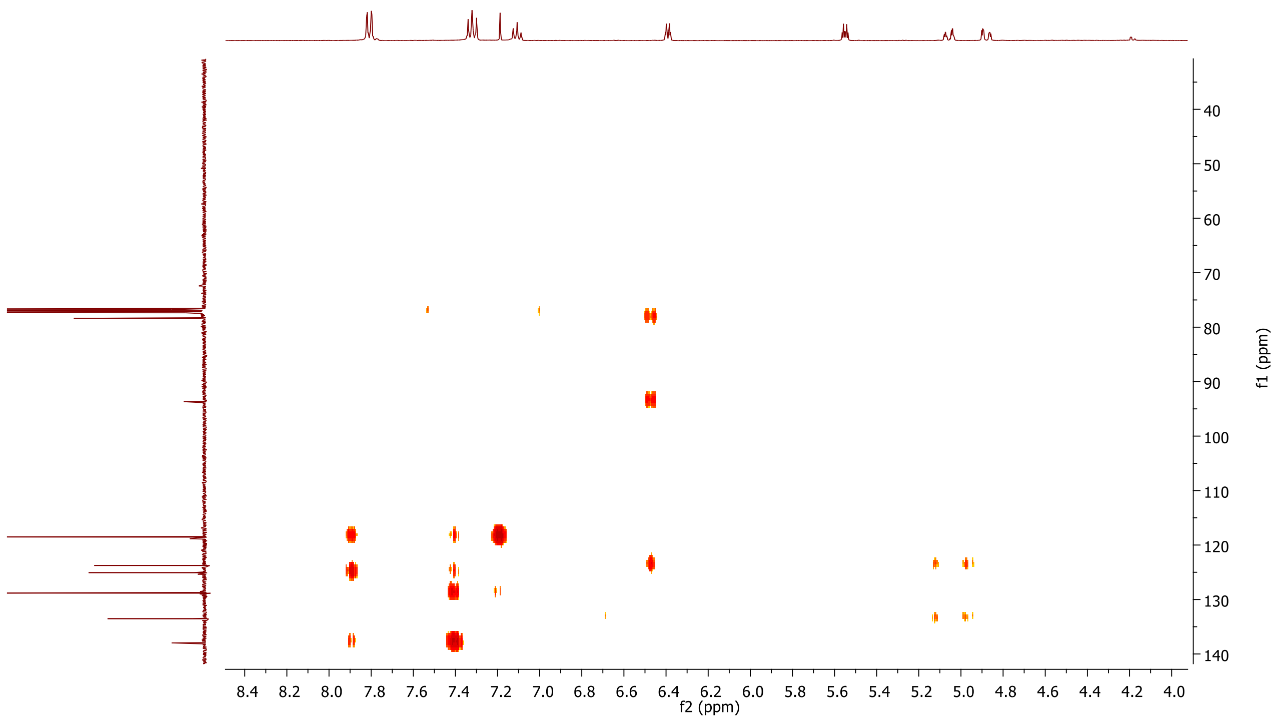
40
50
60
70
80
90
100
110
120
130
140
f1 (ppm)
8.4 8.2 8.0 7.8 7.6 7.4 7.2 7.0 6.8 6.6 6.4 6.2 6.0 5.8 5.6 5.4 5.2 5.0 4.8 4.6 4.4 4.2 4.0
f2 (ppm)

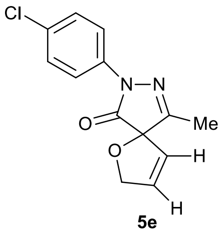
Cl
N–N
O
Me
O
H
H

5e

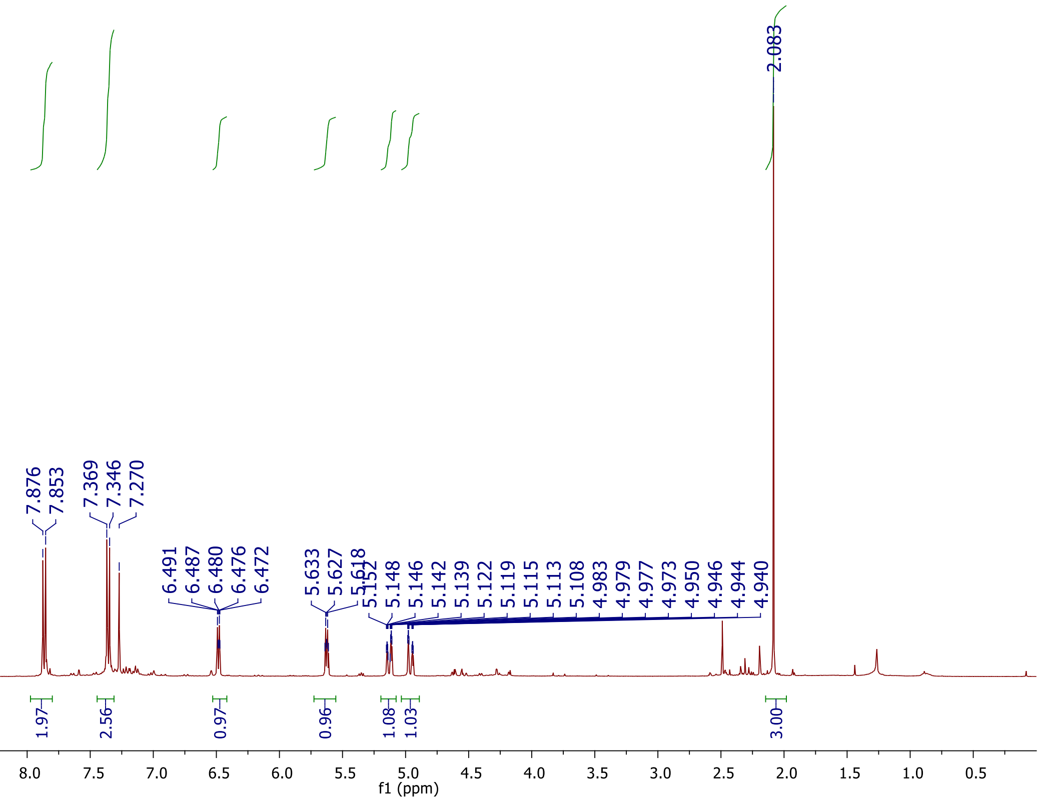
7.876
7.853
7.369
7.346
7.270
6.491
6.487
6.480
6.476
6.472
5.633
5.627
5.618
5.152
5.148
5.146
5.142
5.139
5.122
5.119
5.115
5.113
5.108
4.983
4.979
4.977
4.973
4.950
4.946
4.944
4.940
2.083
1.97
2.56
0.97
0.96
1.08
1.03
3.00
f1 (ppm)

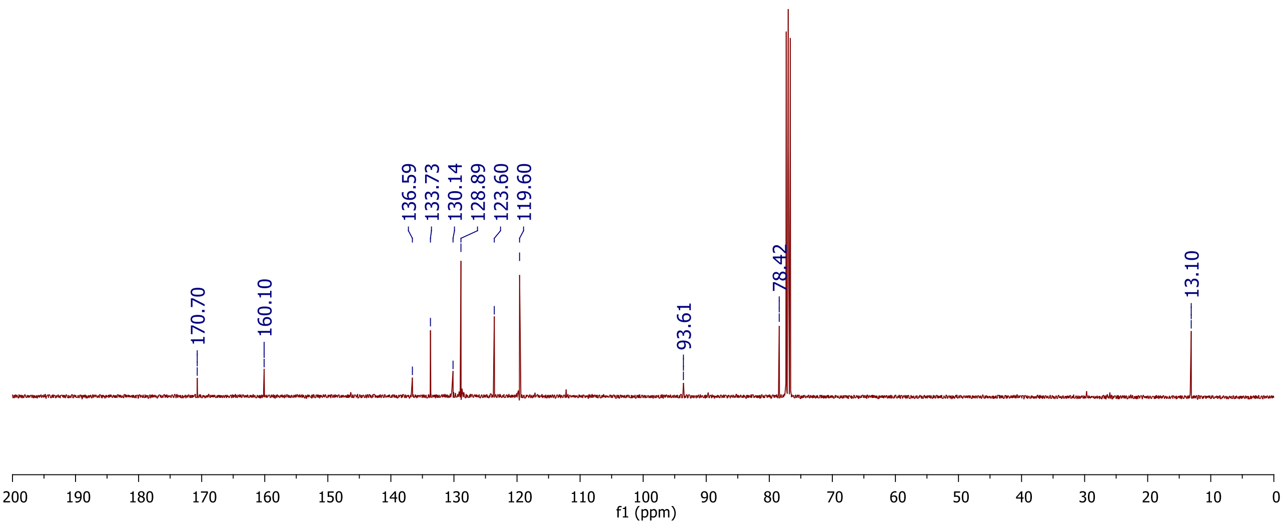
170.70
160.10
136.59
133.73
130.14
128.89
123.60
119.60
93.61
78.42
13.10
f1 (ppm)

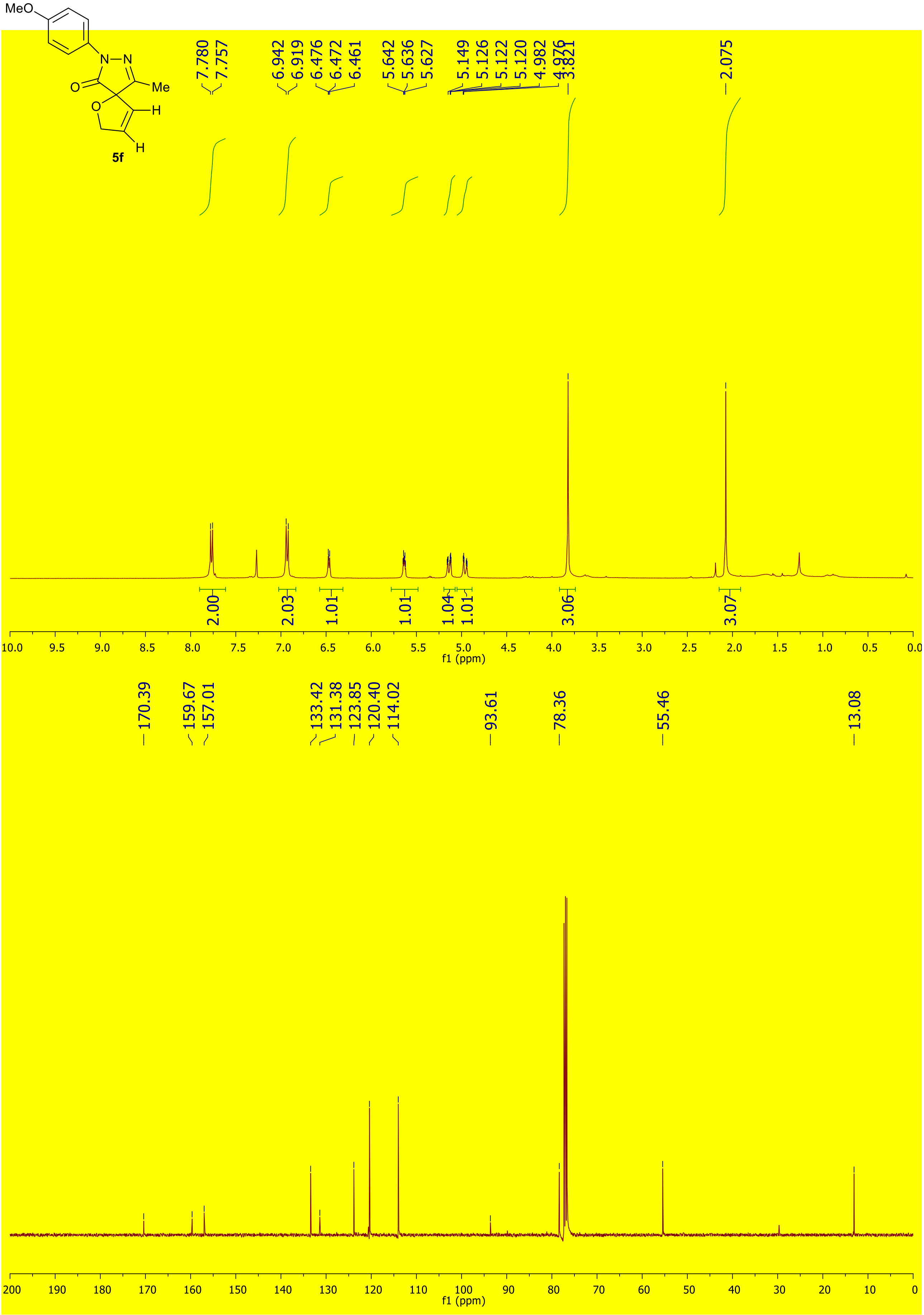
MeO
N–N
O
Me
O
H
H
5f
7.780
7.757
6.942
6.919
6.476
6.472
6.461
5.642
5.636
5.627
5.149
5.126
5.122
5.120
4.982
4.976
3.821
2.075
2.00
2.03
1.01
1.01
1.04
1.01
3.06
3.07
f1 (ppm)
170.39
159.67
157.01
133.42
131.38
123.85
120.40
114.02
93.61
78.36
55.46
13.08
f1 (ppm)

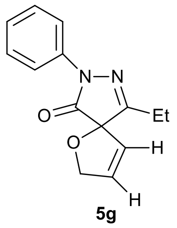

N
N
O
Et
O
H
H

5g

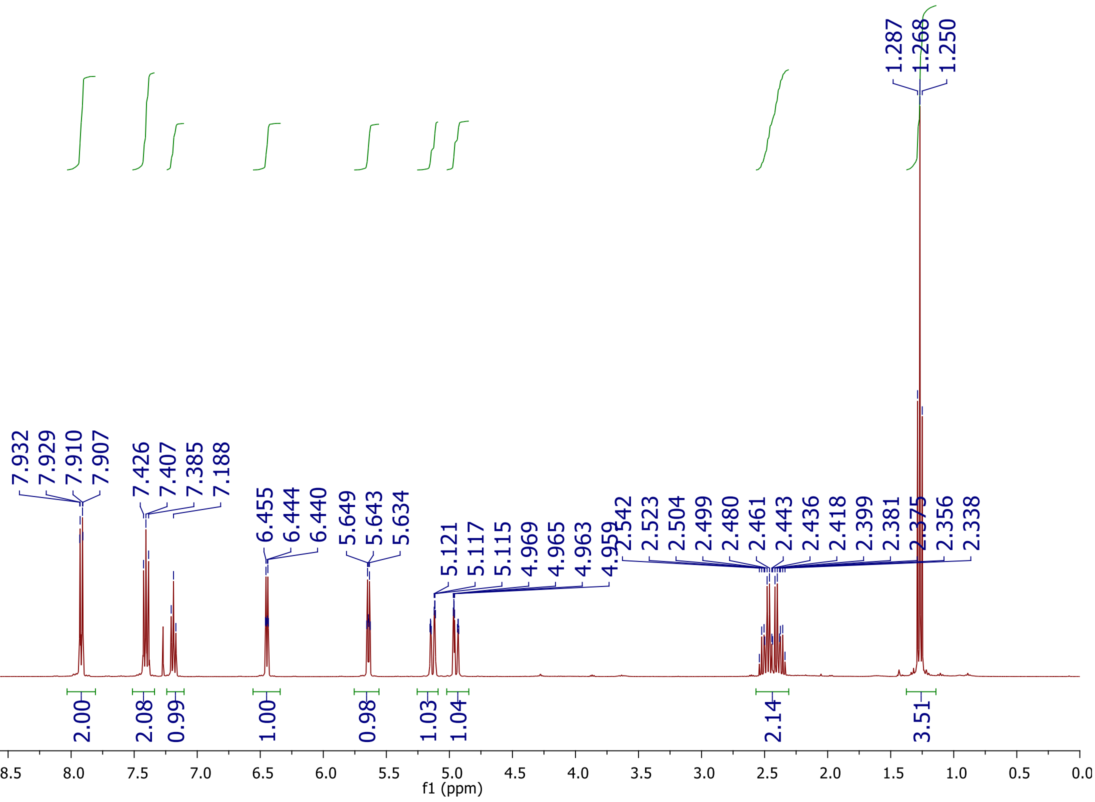

7.932
7.929
7.910
7.907
7.426
7.407
7.385
7.188
6.455
6.444
6.440
5.649
5.643
5.634
5.121
5.117
5.115
4.969
4.965
4.963
4.959
2.542
2.523
2.504
2.499
2.480
2.461
2.443
2.436
2.418
2.399
2.381
2.375
2.356
2.338
1.287
1.268
1.250
2.00
2.08
0.99
1.00
0.98
1.03
1.04
2.14
3.51
10.0 9.5 9.0 8.5 8.0 7.5 7.0 6.5 6.0 5.5 5.0 4.5 4.0 3.5 3.0 2.5 2.0 1.5 1.0 0.5 0.0
f1 (ppm)

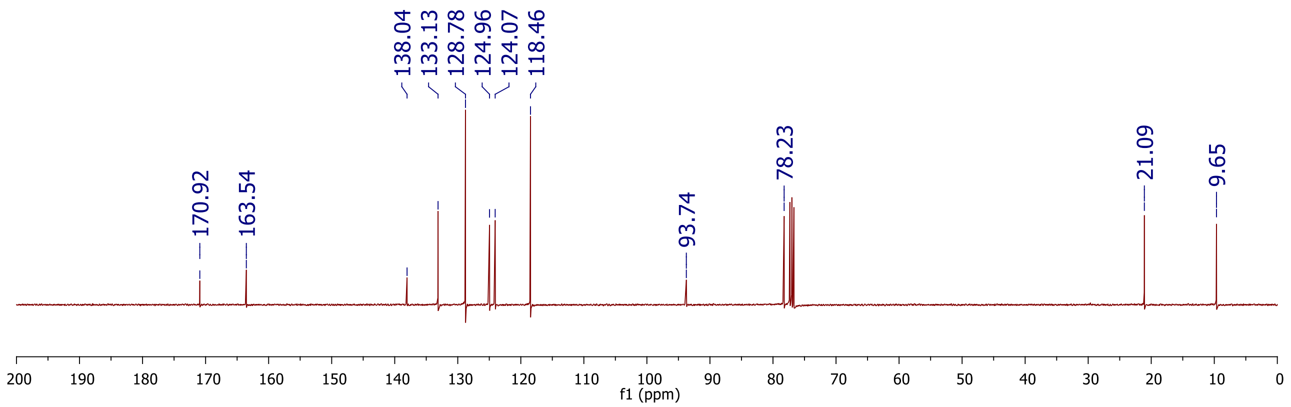

170.92
163.54
138.04
133.13
128.78
124.96
124.07
118.46
93.74
78.23
21.09
9.65
200 190 180 170 160 150 140 130 120 110 100 90 80 70 60 50 40 30 20 10 0
f1 (ppm)

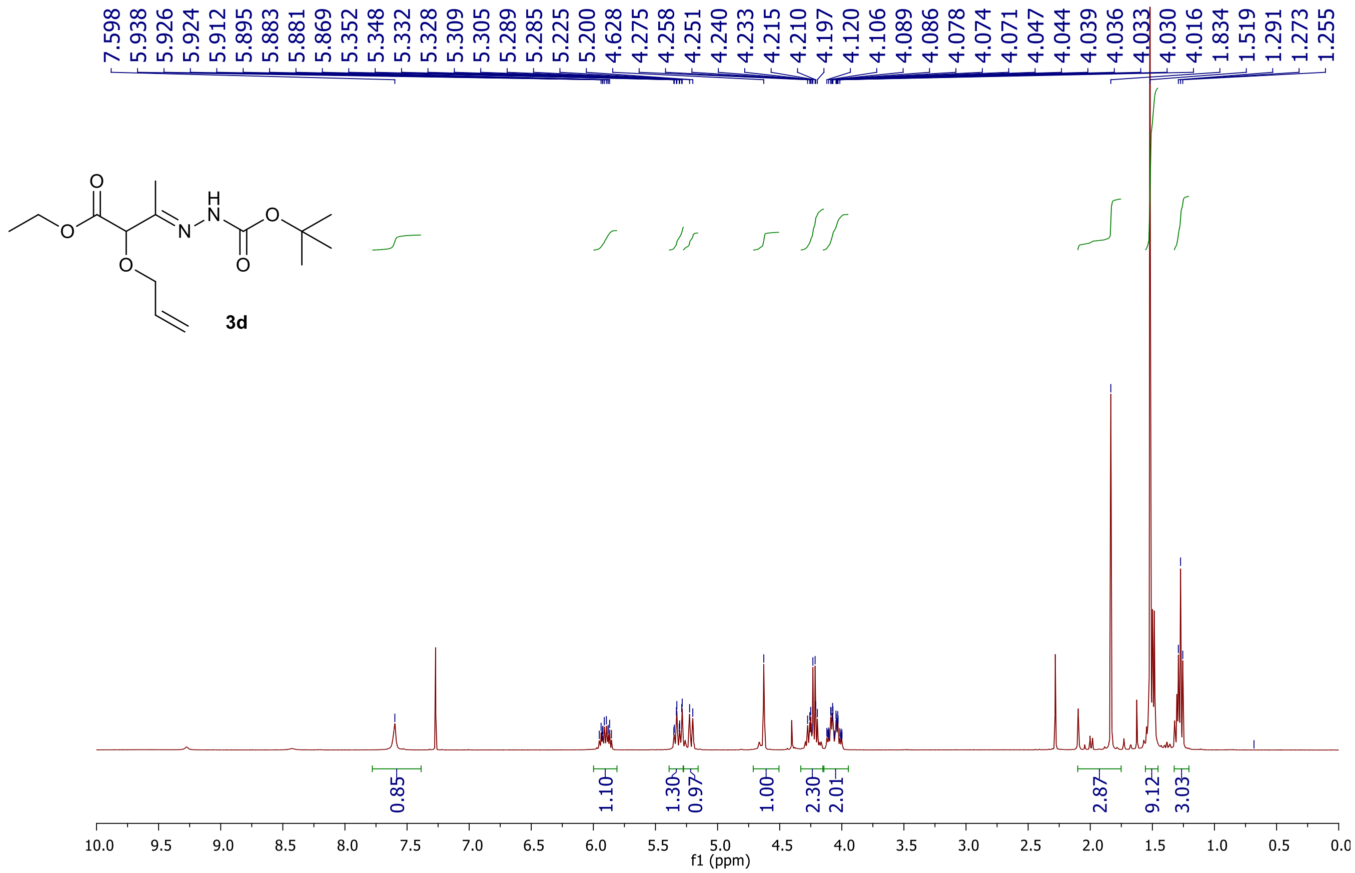

3d

7.598 5.938 5.926 5.924 5.912 5.895 5.883 5.881 5.869 5.352 5.348 5.332 5.328 5.309 5.305 5.289 5.285 5.225 5.200 4.628 4.275 4.258 4.251 4.240 4.233 4.215 4.210 4.197 4.120 4.106 4.089 4.086 4.078 4.074 4.071 4.047 4.044 4.039 4.036 4.033 4.030 4.016 1.834 1.519 1.291 1.273 1.255
0.85 1.10 1.30 0.97 1.00 2.30 2.01 2.87 9.12 3.03
10.0 9.5 9.0 8.5 8.0 7.5 7.0 6.5 6.0 5.5 5.0 4.5 4.0 3.5 3.0 2.5 2.0 1.5 1.0 0.5 0.0
f1 (ppm)

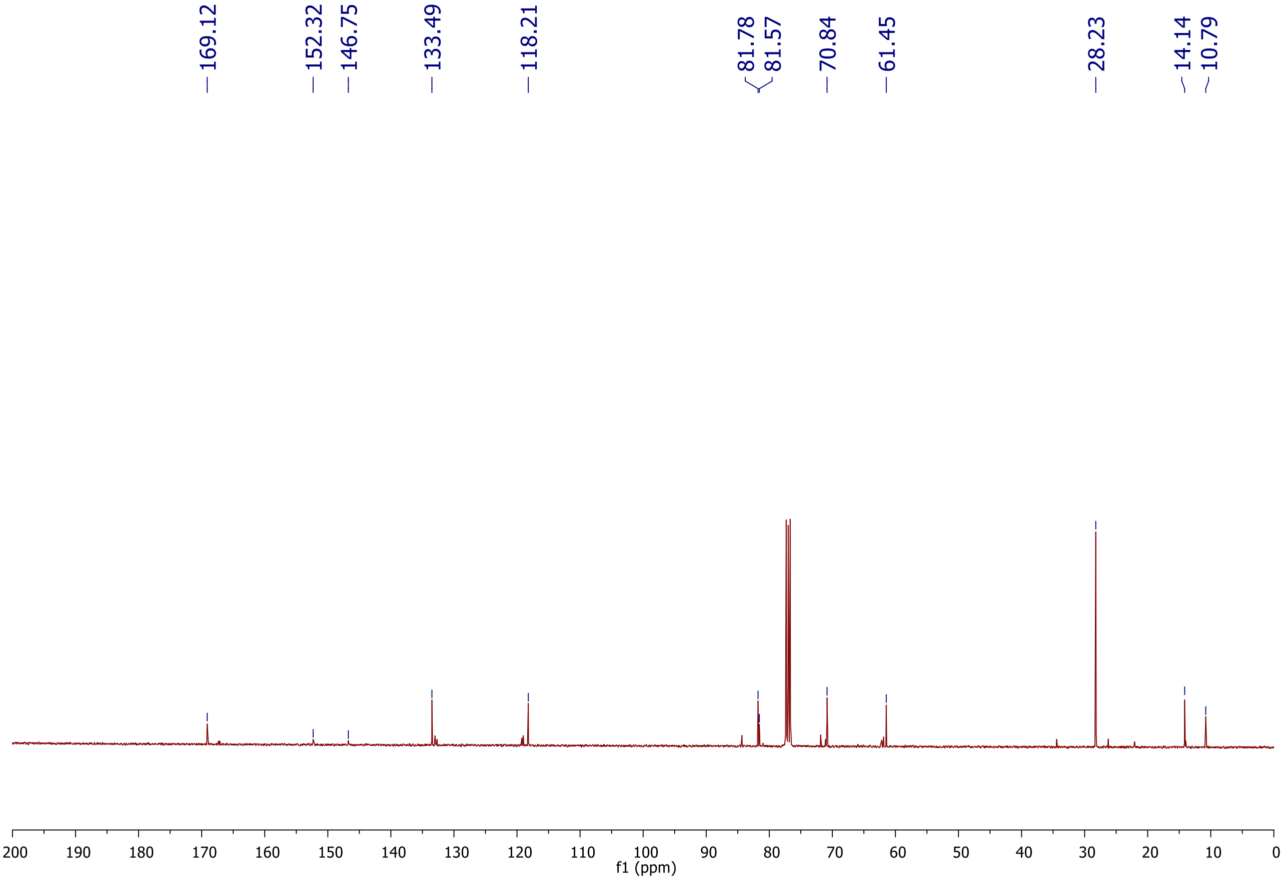

169.12 152.32 146.75 133.49 118.21 81.78 81.57 70.84 61.45 28.23 14.14 10.79
200 190 180 170 160 150 140 130 120 110 100 90 80 70 60 50 40 30 20 10 0
f1 (ppm)

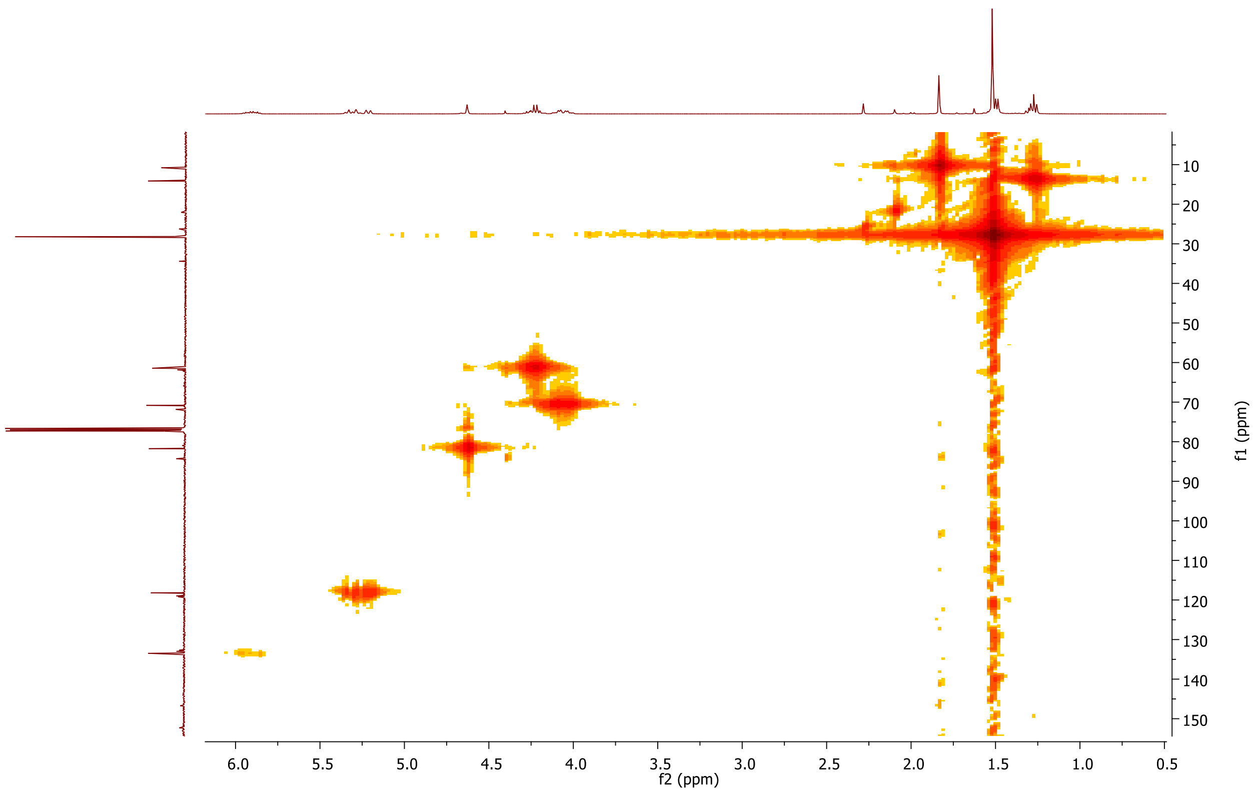

f1 (ppm)
f2 (ppm)

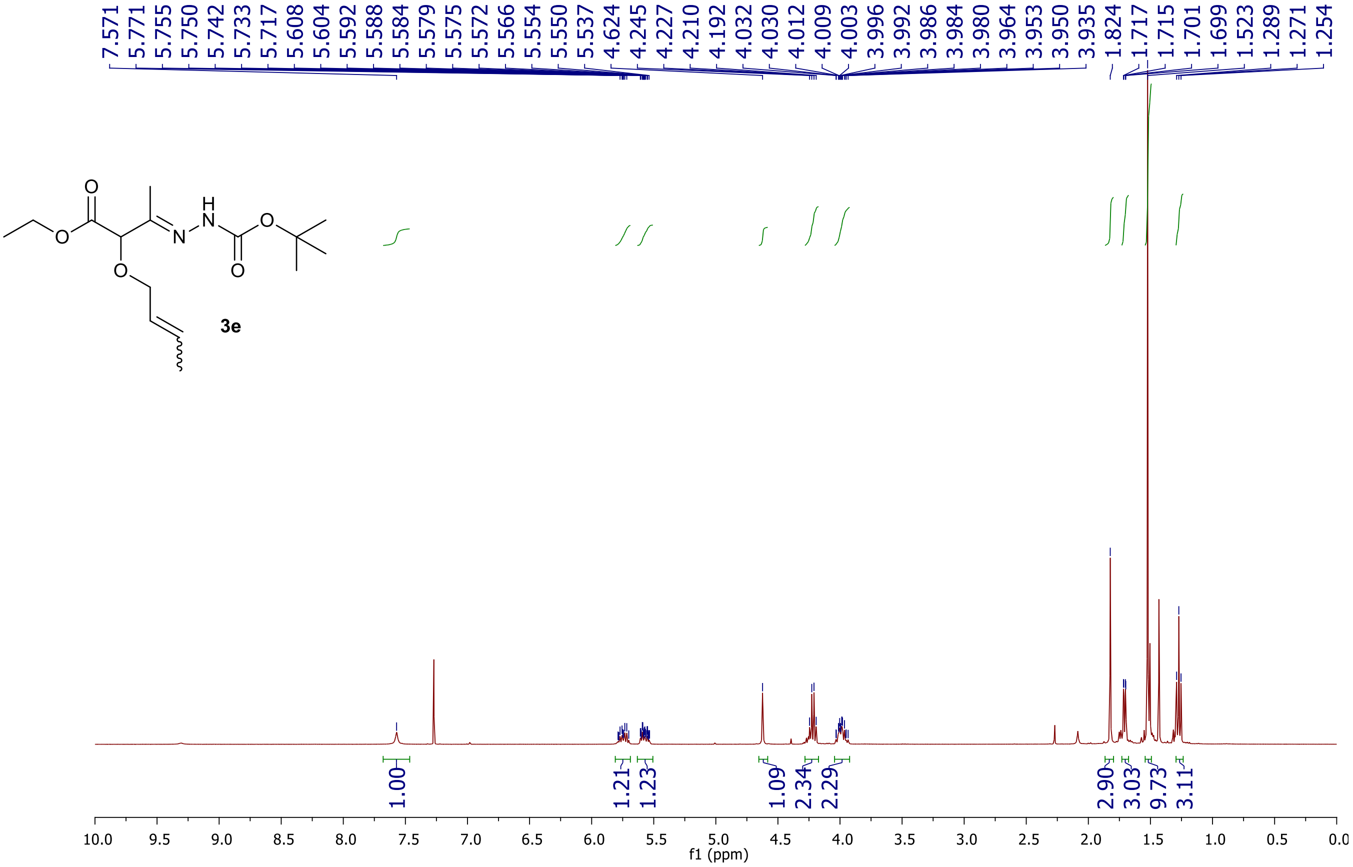
3e
7.571 5.771 5.755 5.750 5.742 5.733 5.717 5.608 5.604 5.592 5.588 5.584 5.579 5.575 5.572 5.566 5.554 5.550 5.537 4.624 4.245 4.227 4.210 4.192 4.032 4.030 4.012 4.009 4.003 3.996 3.992 3.986 3.984 3.980 3.964 3.953 3.950 3.935 1.824 1.717 1.715 1.701 1.699 1.523 1.289 1.271 1.254
1.00 1.21 1.23 1.09 2.34 2.29 2.90 3.03 9.73 3.11
f1 (ppm)

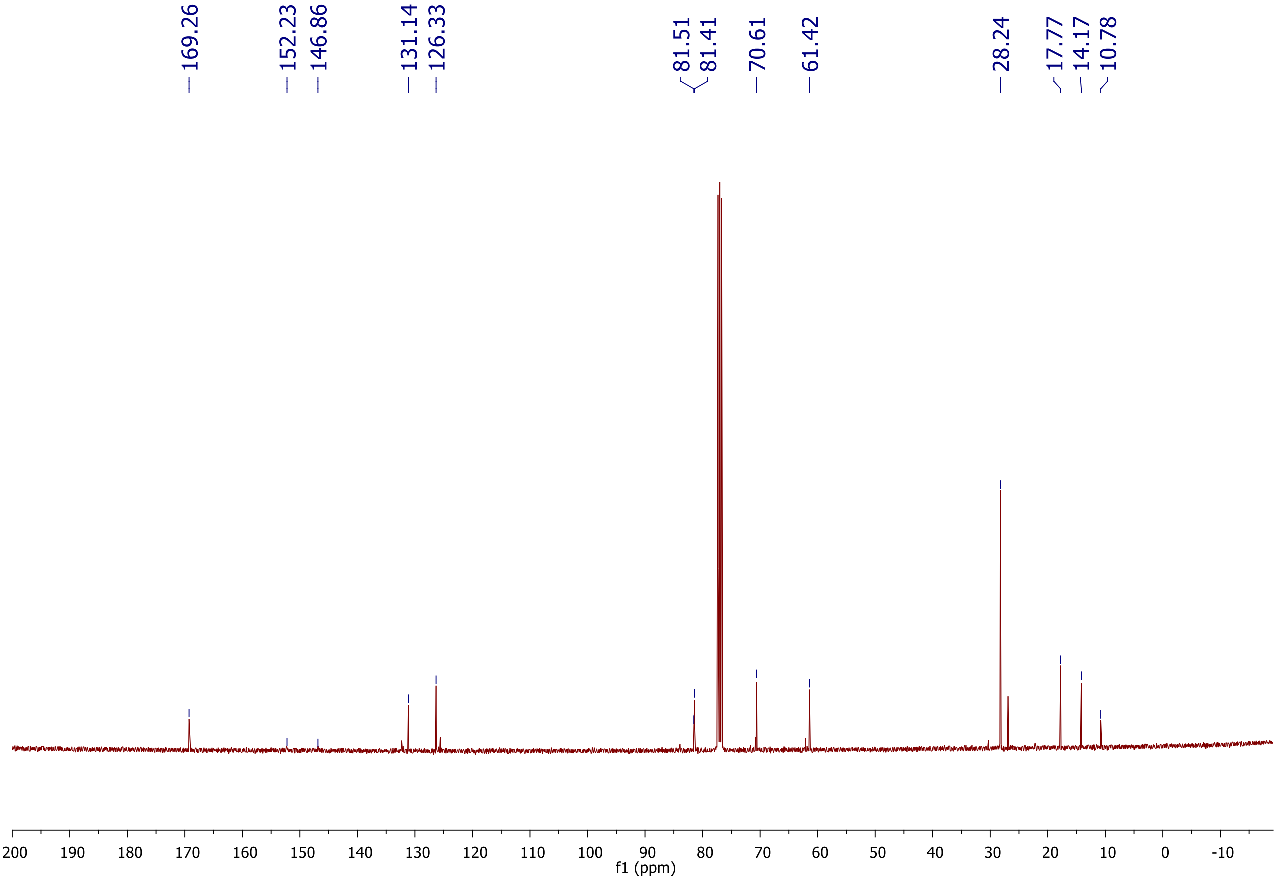
169.26 152.23 146.86 131.14 126.33 81.51 81.41 70.61 61.42 28.24 17.77 14.17 10.78
f1 (ppm)

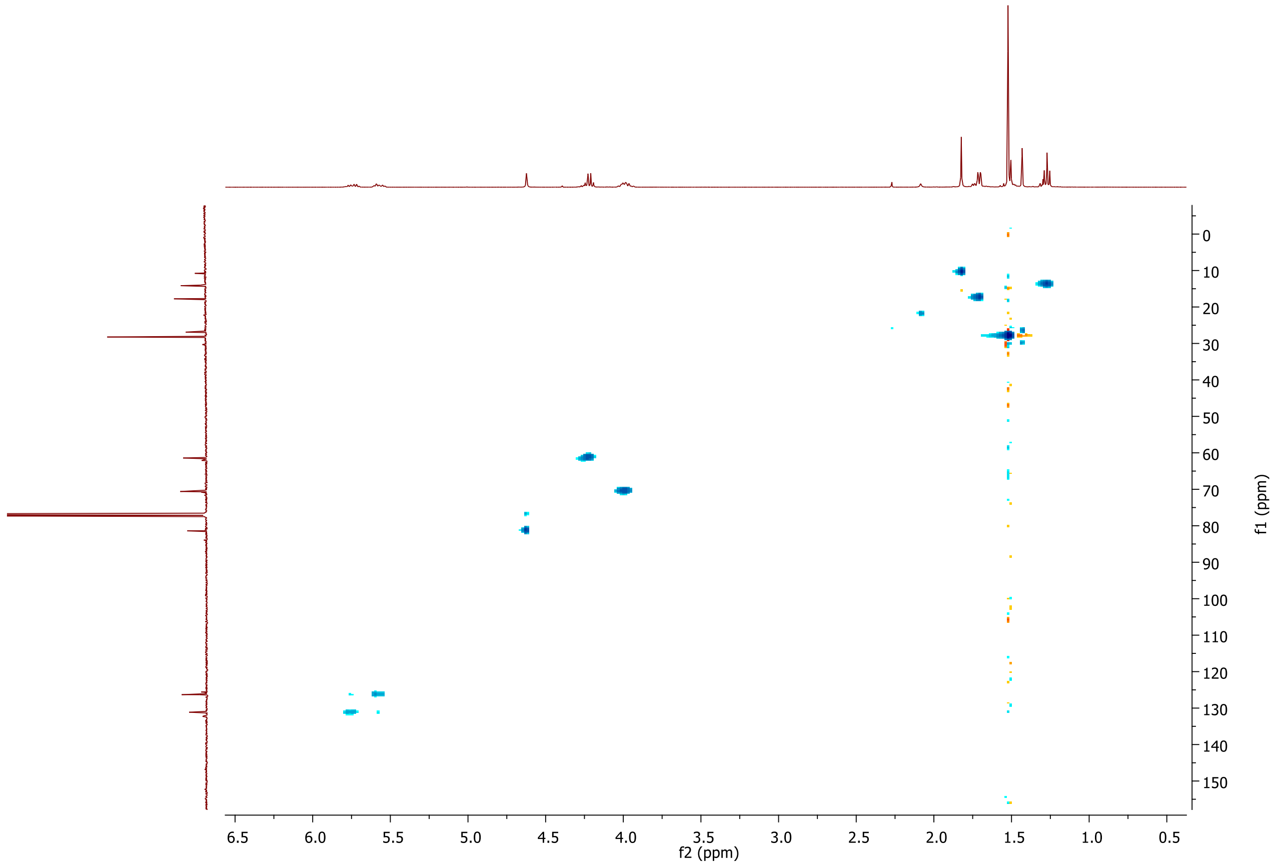

f2 (ppm)
f1 (ppm)

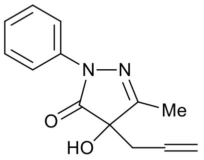
N
N
Me
O
HO

6a

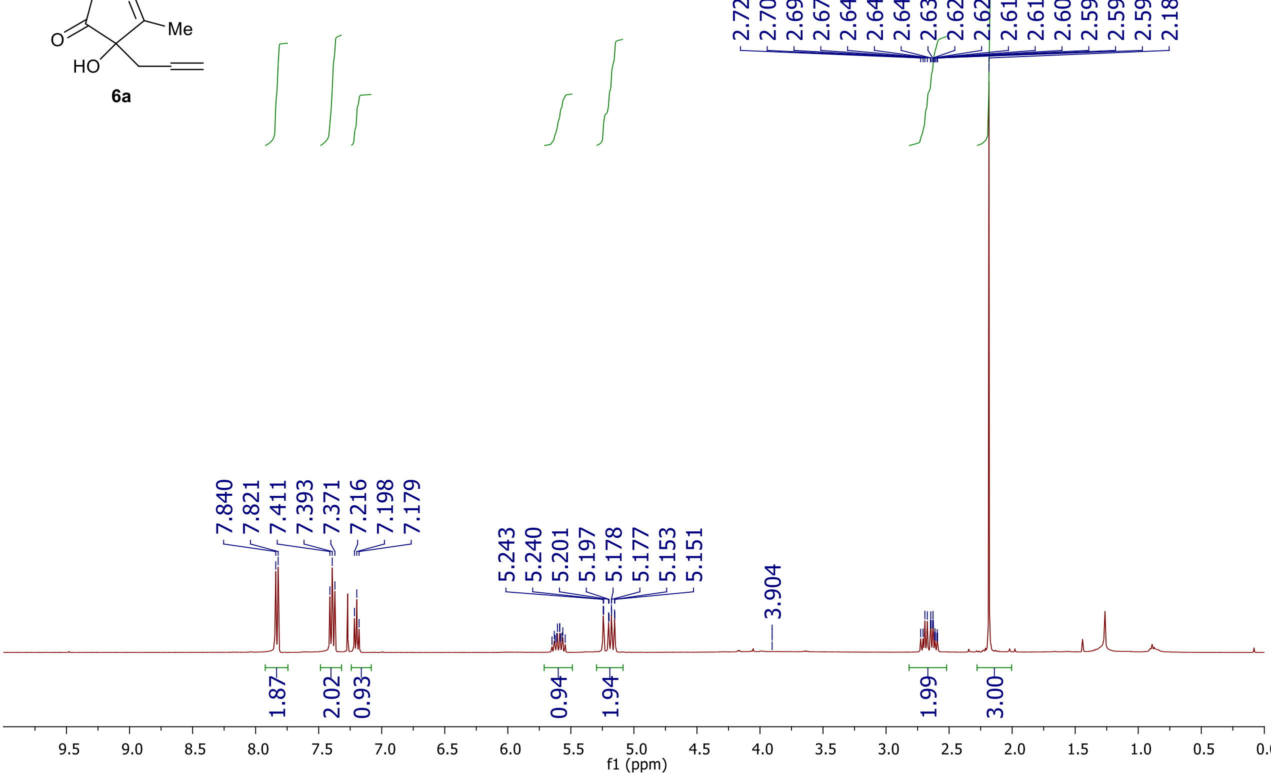
2.726
2.706
2.692
2.673
2.648
2.645
2.643
2.630
2.628
2.625
2.615
2.612
2.609
2.597
2.594
2.592
2.184
7.840
7.821
7.411
7.393
7.371
7.216
7.198
7.179
5.243
5.240
5.201
5.197
5.178
5.177
5.153
5.151
3.904
1.87
2.02
0.93
0.94
1.94
1.99
3.00
9.5 9.0 8.5 8.0 7.5 7.0 6.5 6.0 5.5 5.0 4.5 4.0 3.5 3.0 2.5 2.0 1.5 1.0 0.5 0.0
f1 (ppm)

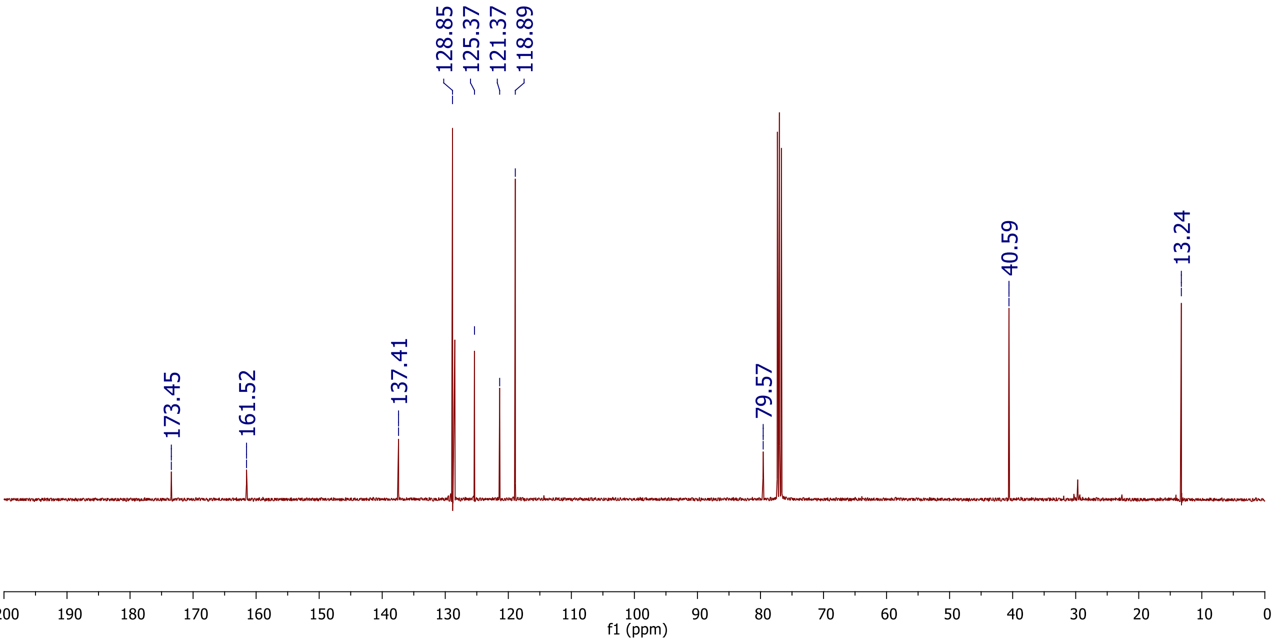
128.85
125.37
121.37
118.89
173.45
161.52
137.41
79.57
40.59
13.24
200 190 180 170 160 150 140 130 120 110 100 90 80 70 60 50 40 30 20 10 0
f1 (ppm)

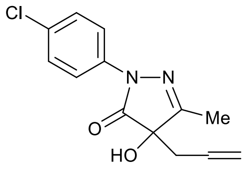
Cl
N
N
Me
O
HO

6b

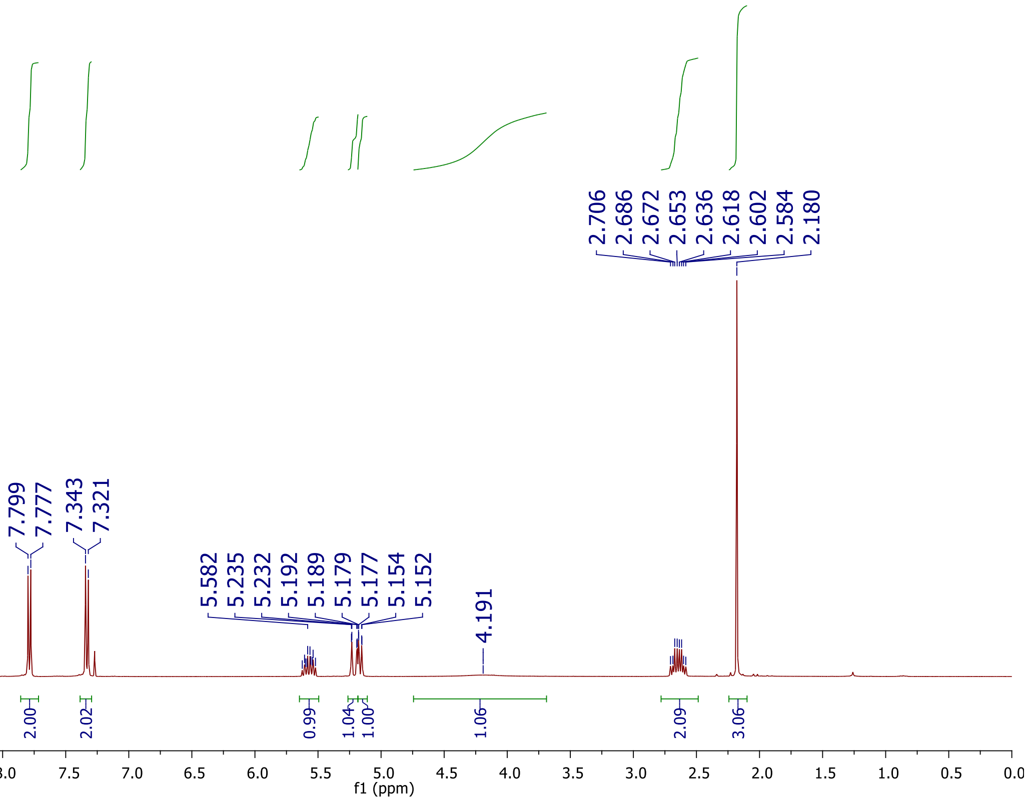
7.799
7.777
7.343
7.321
5.582
5.235
5.232
5.192
5.189
5.179
5.177
5.154
5.152
4.191
2.706
2.686
2.672
2.653
2.636
2.618
2.602
2.584
2.180
2.00
2.02
0.99
1.04
1.00
1.06
2.09
3.06
f1 (ppm)

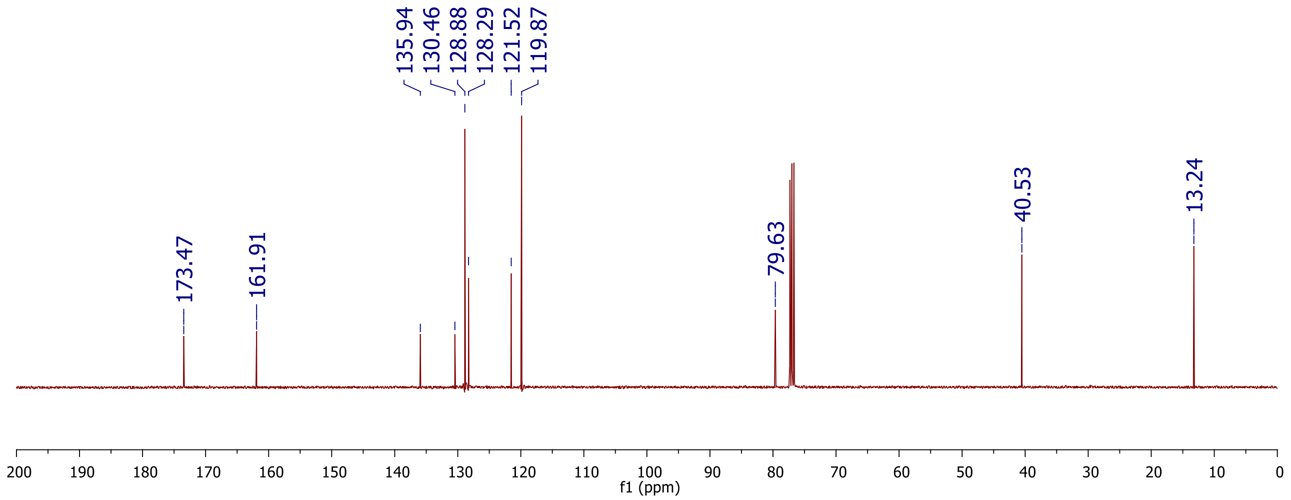
173.47
161.91
135.94
130.46
128.88
128.29
121.52
119.87
79.63
40.53
13.24
f1 (ppm)

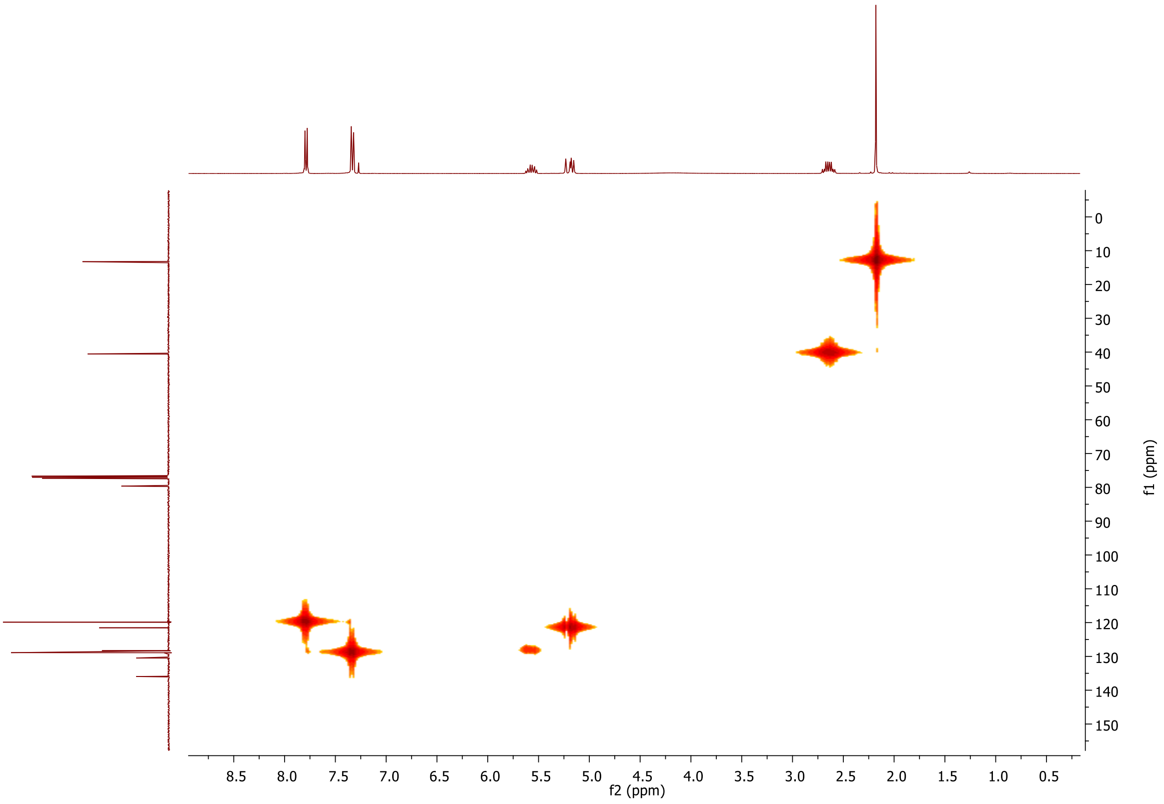
f2 (ppm)
f1 (ppm)

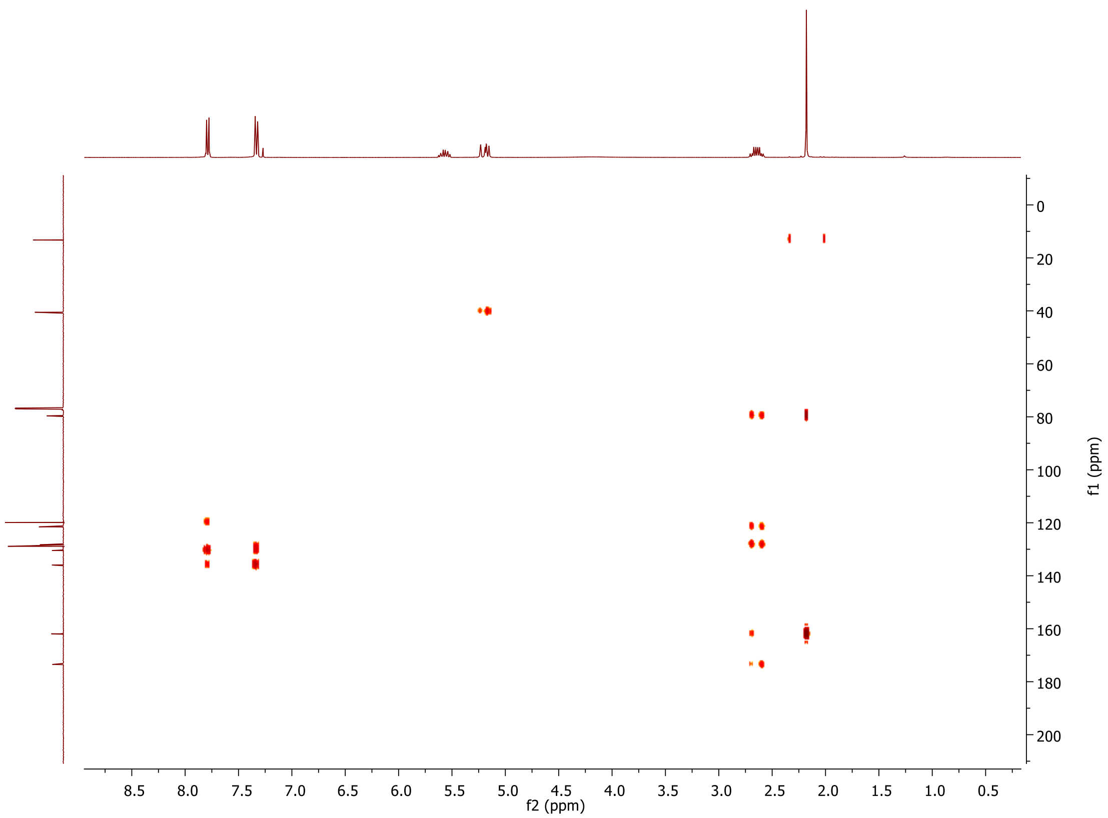
f2 (ppm)
f1 (ppm)

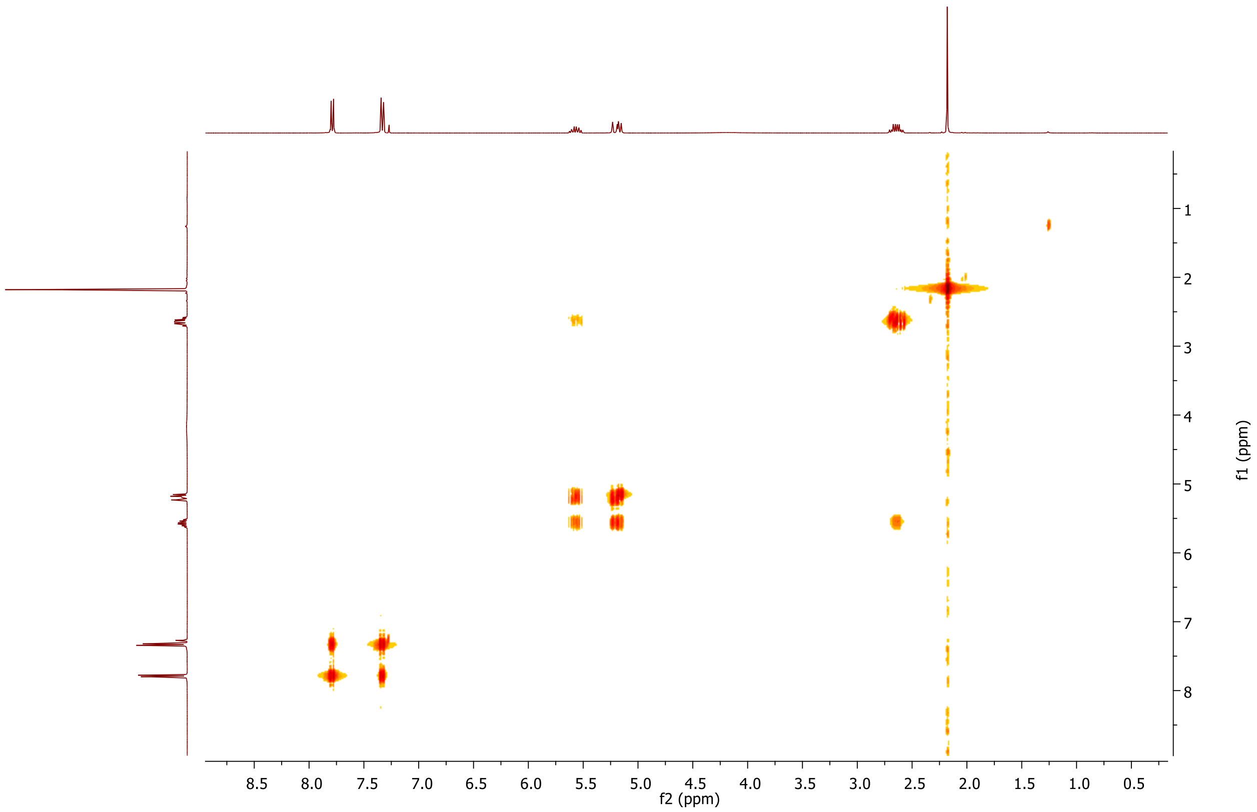
f2 (ppm)
f1 (ppm)

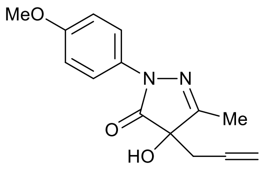
MeO
N
N
Me
O
HO

6c

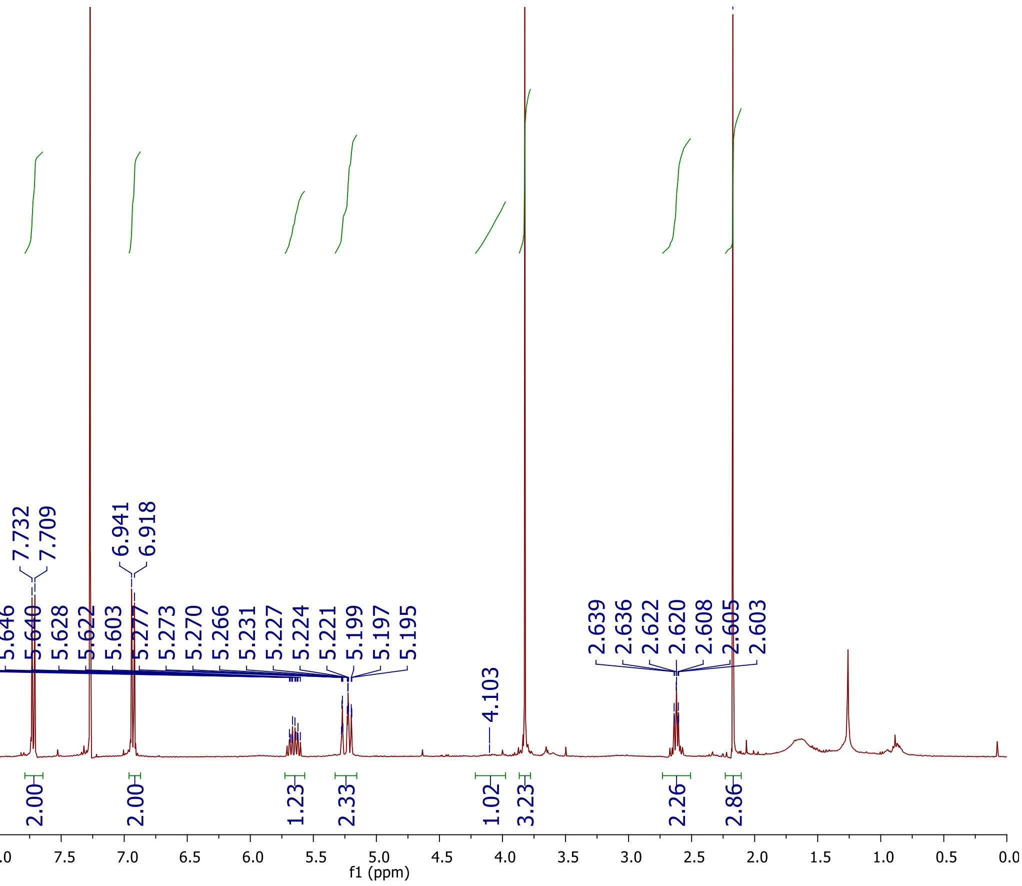
7.732
7.709
6.941
6.918
5.690
5.683
5.671
5.665
5.646
5.640
5.628
5.622
5.603
5.277
5.273
5.270
5.266
5.231
5.227
5.224
5.221
5.199
5.197
5.195
4.103
2.639
2.636
2.622
2.620
2.608
2.605
2.603
2.00
2.00
1.23
2.33
1.02
3.23
2.26
2.86
10.0 9.5 9.0 8.5 8.0 7.5 7.0 6.5 6.0 5.5 5.0 4.5 4.0 3.5 3.0 2.5 2.0 1.5 1.0 0.5 0.0
f1 (ppm)

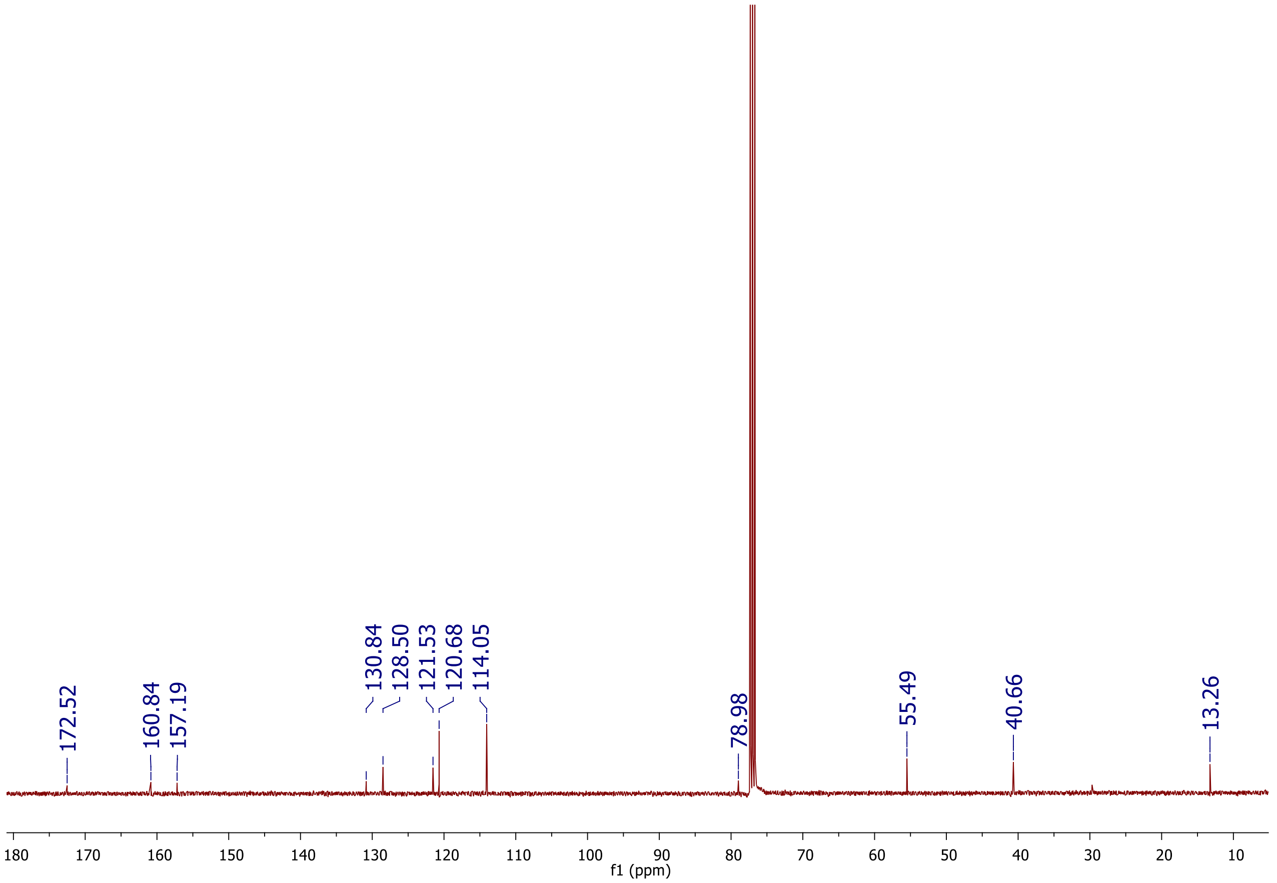
172.52
160.84
157.19
130.84
128.50
121.53
120.68
114.05
78.98
55.49
40.66
13.26
180 170 160 150 140 130 120 110 100 90 80 70 60 50 40 30 20 10
f1 (ppm)

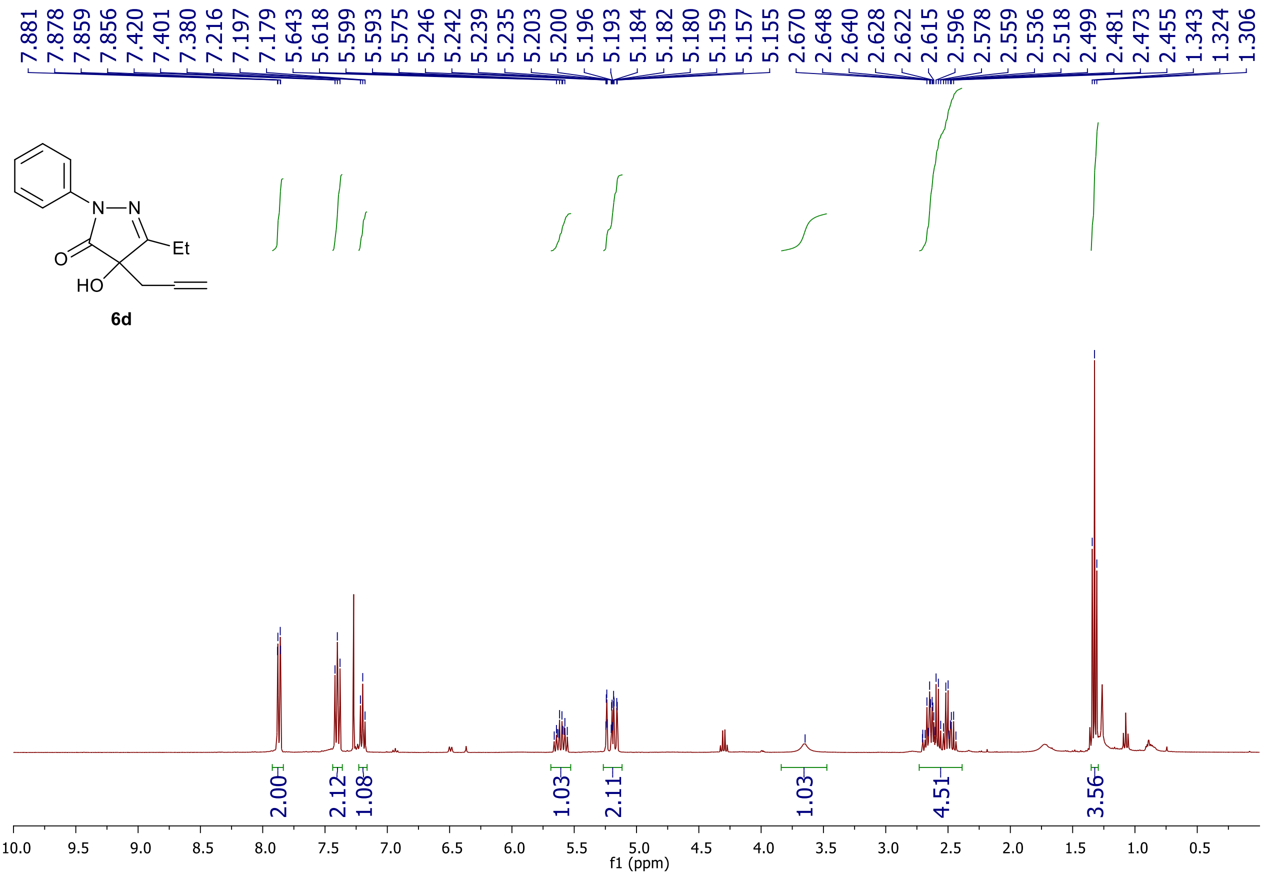

7.881
7.878
7.859
7.856
7.420
7.401
7.380
7.216
7.197
7.179
5.643
5.618
5.599
5.593
5.575
5.246
5.242
5.239
5.235
5.203
5.200
5.196
5.193
5.184
5.182
5.180
5.159
5.157
5.155
2.670
2.648
2.640
2.628
2.622
2.615
2.596
2.578
2.559
2.536
2.518
2.499
2.481
2.473
2.455
1.343
1.324
1.306
N
N
Et
O
HO
6d
2.00
2.12
1.08
1.03
2.11
1.03
4.51
3.56
10.0
9.5
9.0
8.5
8.0
7.5
7.0
6.5
6.0
5.5
5.0
4.5
4.0
3.5
3.0
2.5
2.0
1.5
1.0
0.5
f1 (ppm)

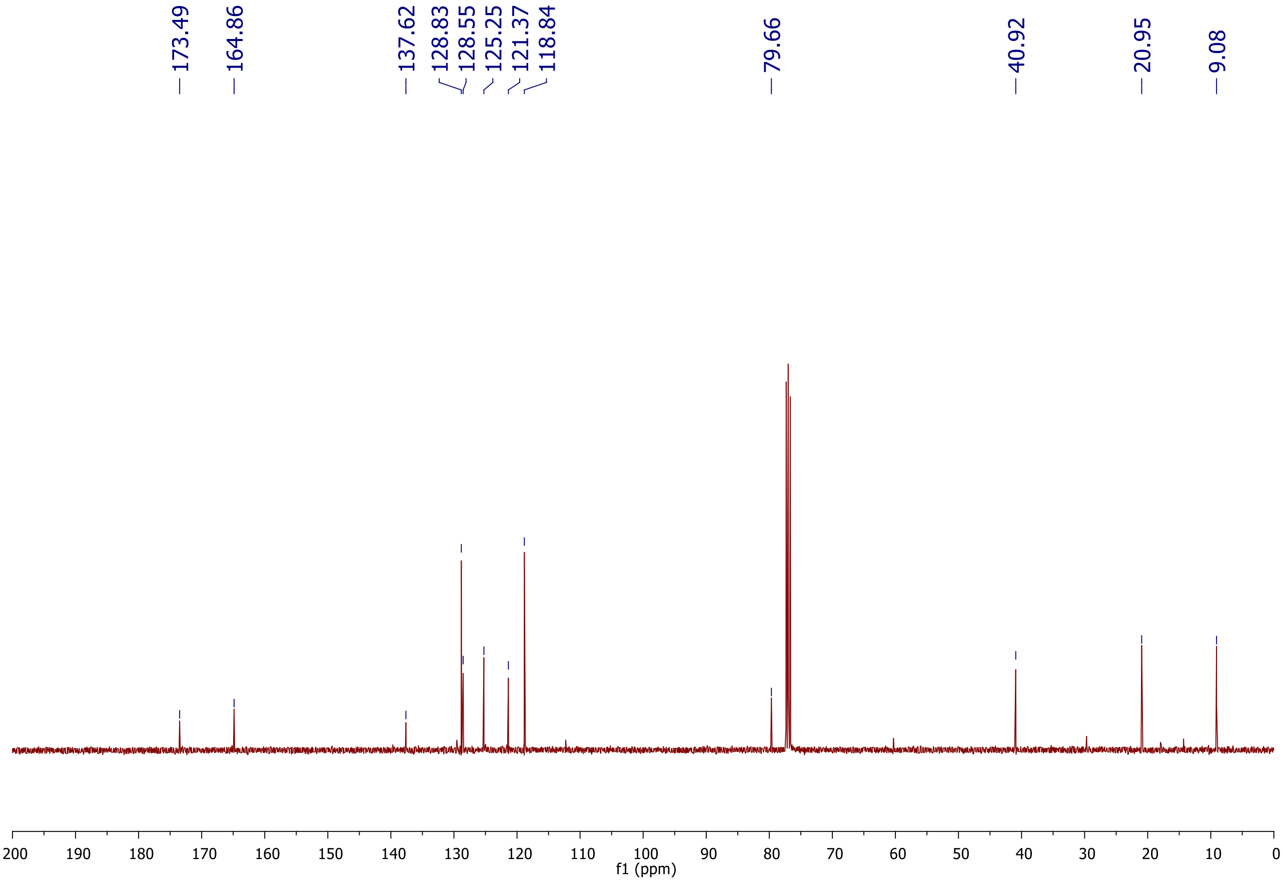

173.49
164.86
137.62
128.83
128.55
125.25
121.37
118.84
79.66
40.92
20.95
9.08
200
190
180
170
160
150
140
130
120
110
100
90
80
70
60
50
40
30
20
10
0
f1 (ppm)

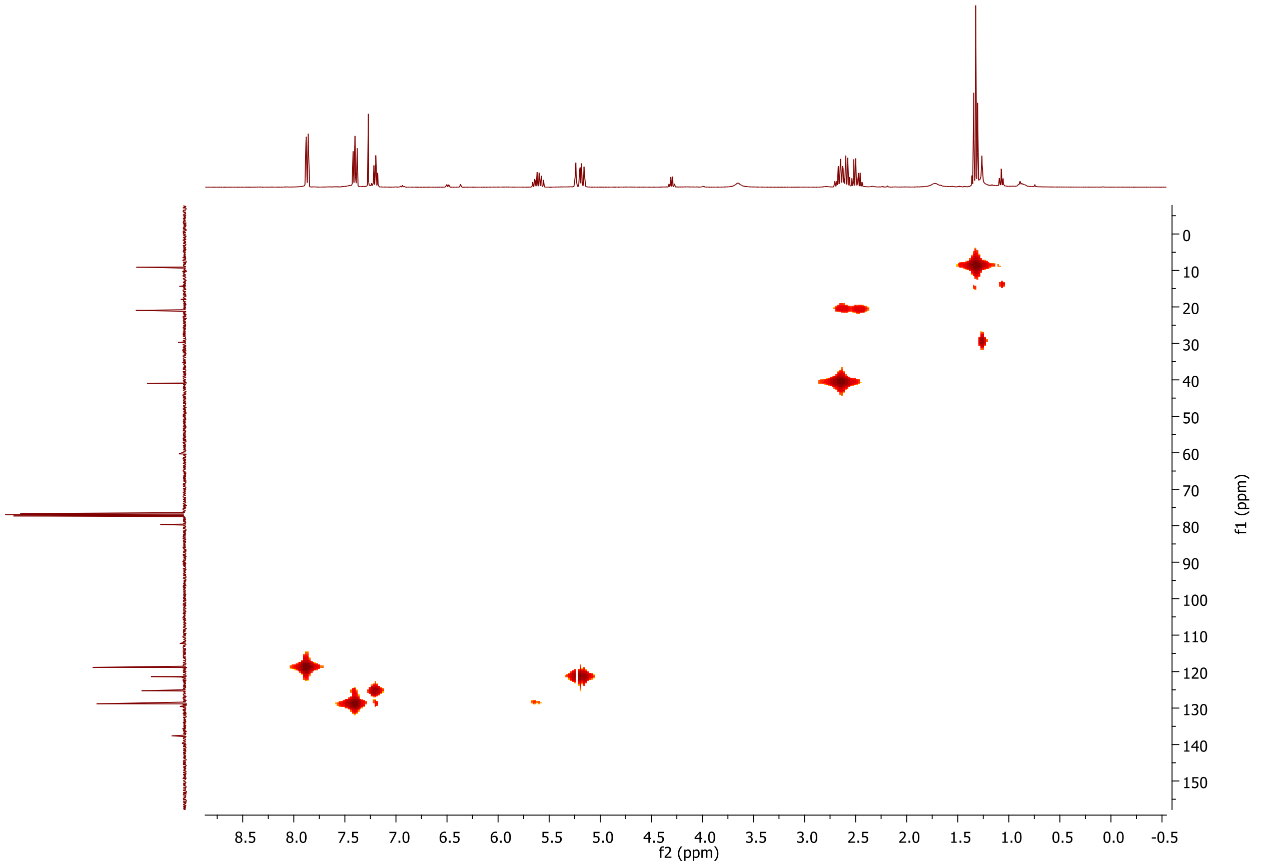

8.5
8.0
7.5
7.0
6.5
6.0
5.5
5.0
4.5
4.0
3.5
3.0
2.5
2.0
1.5
1.0
0.5
0.0
-0.5
f2 (ppm)
0
10
20
30
40
50
60
70
80
90
100
110
120
130
140
150
f1 (ppm)

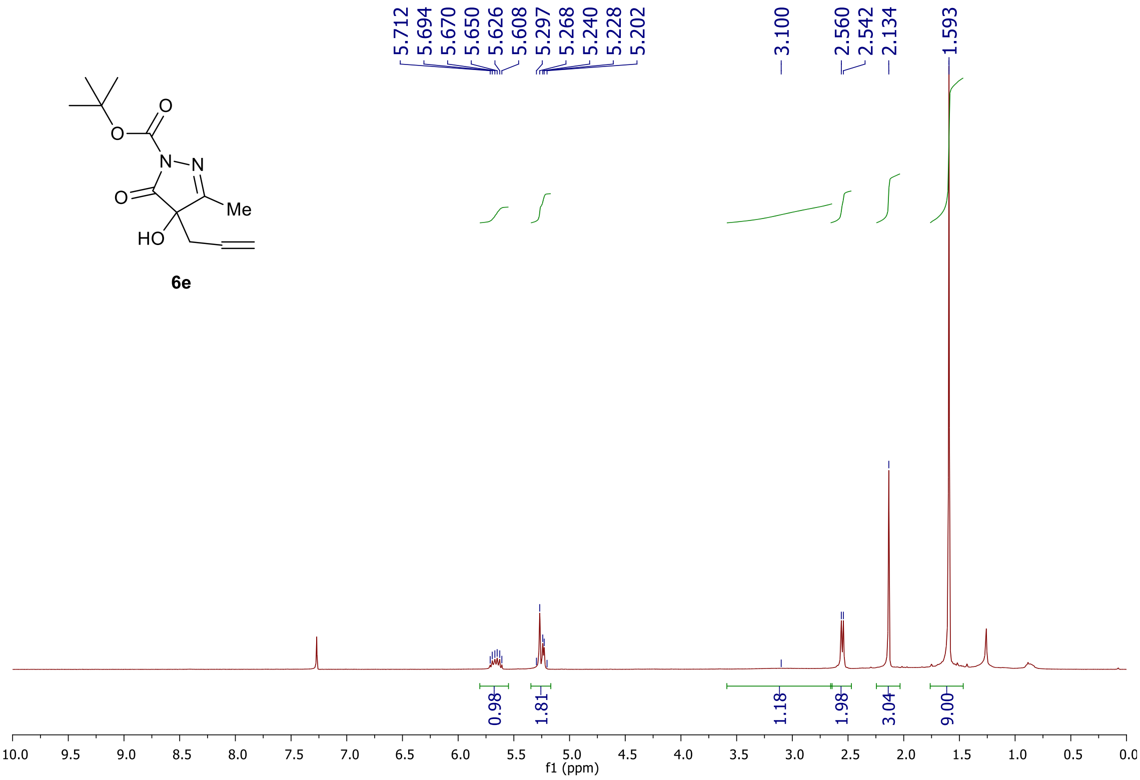
O
O
N
N
O
Me
HO
6e
5.712
5.694
5.670
5.650
5.626
5.608
5.297
5.268
5.240
5.228
5.202
3.100
2.560
2.542
2.134
1.593
0.98
1.81
1.18
1.98
3.04
9.00
10.0 9.5 9.0 8.5 8.0 7.5 7.0 6.5 6.0 5.5 5.0 4.5 4.0 3.5 3.0 2.5 2.0 1.5 1.0 0.5 0.0
f1 (ppm)

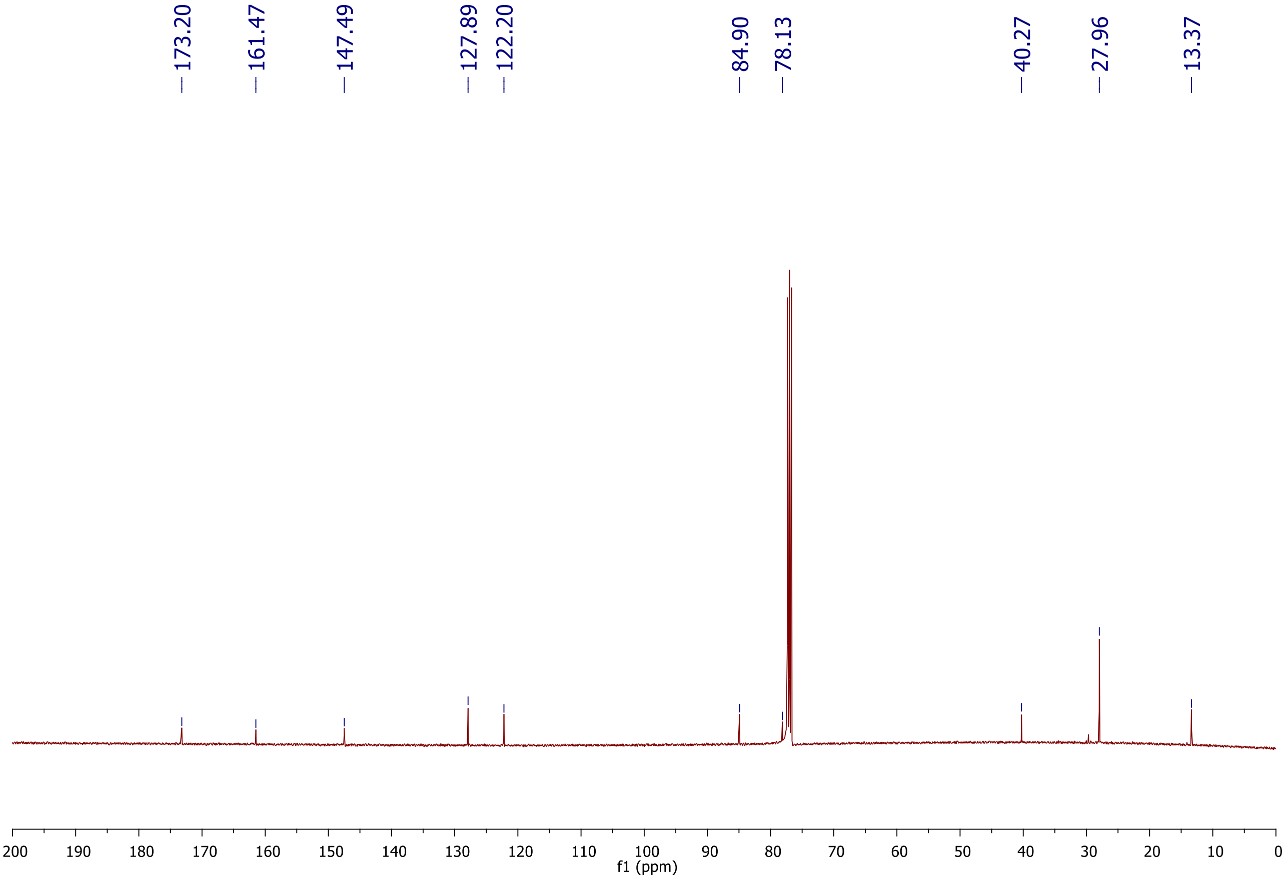
173.20
161.47
147.49
127.89
122.20
84.90
78.13
40.27
27.96
13.37
200 190 180 170 160 150 140 130 120 110 100 90 80 70 60 50 40 30 20 10 0
f1 (ppm)

6. DFT optimized geometries and energies

29

I

G= -762.638451 ha

H	0.65094385016073	0.45956123717303	0.36647656871698
C	0.32211912165911	0.18645158139041	1.36988940057471
C	-0.51414775940142	-0.53725129186878	3.94124694004111
C	0.56722338593556	-1.10552813746985	1.83560132848579
C	-0.33436032103601	1.12328551636414	2.17389682511666
C	-0.74662413325344	0.75099823064081	3.45675382170635
C	0.14805008917492	-1.47288963379147	3.12606213597188
H	1.07693536557474	-1.83405083628576	1.20971519494572
H	-0.52351879699131	2.13155764377248	1.80458514390921
H	-1.26102976397859	1.46983890924901	4.09637530852996
H	-0.83921008462009	-0.82200524443379	4.93741353518091
N	0.40238502261649	-2.78440390114653	3.59040353855318
N	0.91364440965443	-3.73290947202527	2.67207602724213
C	1.04481897203475	-4.86737342477389	3.27514801855753
C	1.52002767693619	-6.09454365720667	2.59285907823589
H	2.44875378660244	-6.45601322004967	3.05983562326647
H	1.69718967110561	-5.90226822114674	1.52831888258814
H	0.77377345353410	-6.89653166265196	2.70265269715890
C	0.18334986721940	-3.33196184262962	4.84279656096858
O	-0.30324522301092	-2.79006202163444	5.83165740432416
O	-0.25136933944763	-5.76598860851336	5.11663306195774
C	-0.29890931079497	-5.95162422608064	6.55284838737703
H	0.71678057128556	-6.18322007849221	6.92328769148960
H	-0.63834808852132	-5.02515027806099	7.04391011258198
C	-1.20915722891331	-7.04457471198620	6.85088459528456
H	-2.63803712853552	-8.75704209599401	7.34532642069928
C	-1.96689278897363	-7.95316749316577	7.11217056417192
C	0.69283810151856	-4.78054064076994	4.73387309754918
H	1.62001662246557	-4.86259241841226	5.33830203481442

28

II

G= -762.185274 ha

H	1.296651	0.630363	0.711024
C	0.772430	0.307190	1.612755
C	-0.568225	-0.546516	3.912604
C	0.767371	-1.047047	1.944893
C	0.112774	1.247488	2.413275
C	-0.552700	0.804461	3.561633
C	0.094872	-1.493330	3.102541
H	1.277089	-1.778589	1.321859
H	0.118373	2.305391	2.146441

H	-1.070516	1.521127	4.202452
H	-1.082608	-0.896351	4.804537
N	0.098808	-2.853417	3.425754
N	0.834885	-3.734600	2.633145
C	0.637016	-4.944832	3.180660
C	1.284853	-6.158063	2.595882
H	1.897968	-6.684346	3.344003
H	1.930188	-5.871696	1.754415
H	0.535377	-6.877970	2.231794
C	-0.224688	-4.874897	4.287401
C	-0.599643	-3.520938	4.479480
O	-1.349827	-2.975838	5.346253
O	-0.672206	-5.945377	5.054166
C	-0.222932	-5.840668	6.428780
H	0.881592	-5.805409	6.453612
H	-0.604631	-4.901817	6.865792
C	-0.704455	-6.987281	7.184896
H	-1.457071	-8.790434	8.369701
C	-1.103744	-7.944603	7.813254

28
II’
G= -762.131463 ha (+33.7 kcal/mol with respect to II)

H	-0.83566582371782	-0.07892438792224	0.20273805565639
C	-0.52654492581661	-0.09667903973193	1.24877472375725
C	0.26378687421778	-0.16708636492517	3.93314915217977
C	-0.26688279385759	-1.32399742695144	1.85907724547352
C	-0.39891496176463	1.09798735087761	1.96473735952923
C	-0.00273849485804	1.05070002185847	3.30493355153491
C	0.12790941381108	-1.36480132815879	3.20794022098721
H	-0.36591331400809	-2.25437693863094	1.30447954134094
H	-0.60545446354479	2.05474578191555	1.48391205696006
H	0.10742733851512	1.97437890594744	3.87529920402851
H	0.57229217892693	-0.19863419480745	4.97395176285687
N	0.38921944972162	-2.61061082460556	3.81590539334589
N	0.49319622156442	-3.75672620678471	2.98900130640491
C	0.69516993763779	-4.78796755028338	3.74916637450661
C	0.85763753370319	-6.15354849343666	3.19257934550982
H	1.87120716338501	-6.53255899820840	3.39912242846200
H	0.68365534750552	-6.15471254926085	2.10866076068482
H	0.16051069299771	-6.84300591041828	3.69360613809852
C	0.49705954476528	-2.91943946533518	5.16139899288608
O	0.40616292162174	-2.15337418126312	6.11782787706373
O	-0.34716450344149	-5.02817440206920	5.97895243263539
C	-0.07658266911309	-6.36436510066355	6.39335857832070
H	0.81004626847115	-6.45918014868412	7.02813331725767
C	-0.84379021333361	-7.38355553980049	5.97769926753254

H	-1.51266854384218	-8.97266607011590	4.84169102233756
C	-1.68955979667498	-8.32227839885776	5.71417856393795
C	0.72050990263039	-4.43311640774117	5.21048068634994
H	1.69408971449821	-4.65603213194237	5.68424464036065

28
TS1
G= -762.107186 ha (+49.0 kcal/mol with respect to II)

C	0.833521	0.048228	0.737314
C	0.008147	0.118871	2.054257
O	-0.463124	-0.168837	0.314685
H	7.269123	2.008474	0.165616
C	6.278618	2.398580	0.405718
C	3.727491	3.384279	0.995058
C	5.205656	1.513563	0.517334
C	6.089528	3.774382	0.576634
C	4.810141	4.256595	0.874138
C	3.921248	2.004101	0.807668
H	5.344715	0.443177	0.372913
H	6.930151	4.462758	0.480574
H	4.649321	5.325849	1.024016
H	2.734626	3.760687	1.227967
N	2.830093	1.119601	0.893257
N	3.079674	-0.254056	1.106671
C	1.505065	1.368894	0.531283
O	1.018923	2.447120	0.171208
C	1.957486	-0.902177	0.890328
C	1.900459	-2.378760	1.040796
H	2.848450	-2.750099	1.452630
H	1.720664	-2.879737	0.075718
H	1.073325	-2.663834	1.706174
H	0.765068	0.312254	2.948455
H	-0.572780	1.044630	2.129524
C	-0.640990	-1.066643	2.580423
H	-1.707278	-2.869772	3.498617
C	-1.206770	-2.016557	3.086584

28
III
G= -762.191648 ha (-4.0 kcal/mol with respect to II)

H	1.327839	0.077818	0.544465
C	0.890847	-0.081326	1.531516
C	-0.230658	-0.512068	4.058141
C	0.698569	-1.385747	1.987059
C	0.535330	1.013668	2.326259
C	-0.024871	0.785400	3.587667
C	0.136672	-1.611037	3.257611

H	0.975114	-2.238400	1.370824
H	0.691203	2.031530	1.966620
H	-0.314985	1.627988	4.217576
H	-0.668442	-0.686285	5.037095
N	-0.051261	-2.926750	3.712883
N	-0.007622	-3.987961	2.760019
C	-0.195332	-5.100555	3.395416
C	-0.255372	-6.398205	2.675791
H	0.598585	-7.036016	2.954110
H	-0.257625	-6.250679	1.587541
H	-1.164255	-6.937907	2.981728
C	-0.265254	-3.389164	5.000963
O	-0.312020	-2.707147	6.028263
O	-1.794158	-5.216933	5.149426
C	-0.499097	-4.921612	4.882347
C	0.555388	-5.617377	5.818466
H	0.372068	-5.205741	6.826120
H	1.580946	-5.324895	5.530582
C	0.443315	-7.063753	5.847056
H	0.253642	-9.342336	5.869935
C	0.347434	-8.274509	5.862519

28

TS2

G= -762.202999 ha (+11.1 kcal/mol with respect to II)

C	-0.083291	-0.069478	0.176406
C	-0.182447	-0.128732	2.306752
H	-4.669837	4.715197	-1.167733
C	-4.582474	3.661752	-0.895230
C	-4.331793	0.964012	-0.201672
C	-3.314251	3.113122	-0.705654
C	-5.732452	2.878015	-0.747737
C	-5.592177	1.530381	-0.399502
C	-3.175568	1.754882	-0.357501
H	-2.419045	3.720384	-0.821198
H	-6.721756	3.310967	-0.901580
H	-6.477635	0.904499	-0.273152
H	-4.222933	-0.083176	0.068006
N	-1.896944	1.214982	-0.160285
N	-0.793865	2.083855	-0.123051
C	0.270772	1.315961	0.041545
C	1.641668	1.891791	0.128013
H	2.103578	1.645222	1.096715
H	1.608220	2.983552	0.015837
H	2.292629	1.472258	-0.654219
C	-1.516853	-0.145235	-0.063552
O	-2.273898	-1.134521	-0.163108

O	0.761210	-1.121320	0.023930
C	1.144062	-1.854360	1.334419
H	0.770356	-2.883442	1.222025
H	2.244549	-1.870786	1.337303
C	0.560828	-1.146691	2.458248
H	-0.785114	0.667320	2.722676

28

IV

G= -762.173640 ha (+7.3 kcal/mol with respect to II)

H	0.296318	-0.046249	-0.675626
C	0.162312	0.097856	0.398205
C	-0.170336	0.440628	3.154005
C	0.280606	-1.001547	1.248504
C	-0.120701	1.370347	0.905850
C	-0.283228	1.527677	2.285859
C	0.113798	-0.839164	2.637172
H	0.505212	-1.988892	0.852508
H	-0.212334	2.226550	0.236461
H	-0.505418	2.512088	2.701373
H	-0.299888	0.566770	4.225517
N	0.242807	-1.952303	3.486878
N	0.446145	-3.228570	2.897851
C	0.133431	-2.027475	4.874666
O	-0.140708	-1.107485	5.649039
C	0.495280	-3.467406	5.218902
C	0.513687	-4.097126	3.862207
C	0.679886	-5.551386	3.613230
H	0.722558	-5.764739	2.538142
H	-0.157429	-6.107814	4.061569
H	1.598954	-5.915353	4.097368
O	-0.341372	-4.068027	6.173579
C	0.522222	-4.463290	7.361787
H	0.382017	-5.550364	7.499689
H	0.097715	-3.949479	8.241855
C	1.917657	-4.080132	7.069671
C	1.942180	-3.511090	5.858797
H	2.774630	-3.097027	5.284942

28

TS3

G= -762.204273 ha (+10.3 kcal/mol with respect to II)

O	-0.093514	-0.004048	-0.104157
C	0.181056	0.023652	1.960086
H	5.078997	2.278648	-5.733726
C	4.237048	2.628524	-5.133640
C	2.083333	3.504956	-3.579423

C	3.708556	1.789610	-4.152785
C	3.705429	3.904787	-5.347208
C	2.629164	4.331209	-4.562902
C	2.625192	2.222534	-3.365247
H	4.123516	0.798552	-3.984316
H	4.124162	4.557142	-6.114635
H	2.198479	5.321722	-4.718193
H	1.244565	3.837420	-2.973846
N	2.105546	1.372946	-2.373472
N	2.479949	-0.001216	-2.399888
C	1.224918	1.660089	-1.333696
O	0.672128	2.740876	-1.116821
C	1.136684	0.368613	-0.510519
C	1.904370	-0.580190	-1.391965
C	2.021077	-2.037739	-1.140169
H	2.649642	-2.524342	-1.896467
H	1.020264	-2.495290	-1.146325
H	2.444302	-2.216182	-0.139656
H	-0.064627	-1.018486	2.192224
H	-0.598607	0.740282	2.240368
C	1.491778	0.422316	1.887472
C	2.135014	0.642028	0.761626
H	3.162668	0.952746	0.564780

28
V
G= -762.218713 ha (-21.0 kcal/mol with respect to II)

O	-0.090603	-0.000310	-0.886142
C	-0.065071	-0.106927	2.818847
H	4.881680	2.211466	-6.143787
C	4.141976	2.606528	-5.428938
C	2.252738	3.593440	-3.593871
C	3.683006	1.777963	-4.395018
C	3.671829	3.926083	-5.559529
C	2.727189	4.406260	-4.634228
C	2.731445	2.264749	-3.464256
H	4.042657	0.747189	-4.288055
H	4.036508	4.571933	-6.372881
H	2.344468	5.435356	-4.721301
H	1.520734	3.964682	-2.866655
N	2.282678	1.427886	-2.427673
N	2.658324	0.060091	-2.457381
C	1.461951	1.737173	-1.334294
O	0.988545	2.844845	-1.071572
C	1.169349	0.375469	-0.619040
C	2.128971	-0.521189	-1.422721
C	2.247329	-1.982161	-1.167955

H	2.711598	-2.518645	-2.018201
H	1.221354	-2.367464	-0.990798
H	2.835769	-2.178962	-0.246153
H	-0.159351	-1.133323	3.218196
H	-0.714753	0.664239	3.271940
C	0.785567	0.185261	1.852069
C	1.605414	0.468819	0.859516
H	2.644673	0.791019	1.065930

29

IVH

G = -762.697709 ha

H	0.15826	-0.11345	-1.05424
O	0.61266	0.09479	2.28822
C	-0.80547	-0.02365	2.18540
H	-6.22085	0.56681	6.28711
C	-5.41907	1.16367	5.84983
C	-3.35454	2.67870	4.71874
C	-4.45069	0.53349	5.06796
C	-5.37059	2.54393	6.07001
C	-4.33552	3.29150	5.50058
C	-3.41391	1.29156	4.49912
H	-4.48726	-0.53995	4.89390
H	-6.13145	3.03195	6.68042
H	-4.28036	4.36807	5.66995
H	-2.54909	3.26232	4.28106
N	-2.44117	0.64520	3.70416
N	-2.31945	-0.76558	3.80532
C	-1.53995	1.19236	2.81402
O	-1.36131	2.37356	2.54198
C	-1.38889	-1.15965	3.00348
C	-0.96260	-2.57419	2.88633
H	-1.52678	-3.21007	3.57788
H	0.11297	-2.66002	3.10458
H	-1.10964	-2.93318	1.85588
C	1.20491	0.09719	0.96103
H	1.93197	-0.73051	0.89217
H	1.74708	1.04594	0.80765
C	0.05034	-0.06586	0.02832
C	-1.09549	-0.14117	0.70887
H	-2.10314	-0.25816	0.31618

29

VH

G= -762.672957 ha

H	-0.07312	-0.94015	3.08027
O	-0.16548	-0.16960	2.48259
C	1.79316	-3.04608	3.04178
H	1.69833	7.23541	2.19967
C	1.88422	6.38205	2.85328
C	2.35794	4.18150	4.51710
C	1.64436	5.09314	2.37575
C	2.35158	6.58329	4.15589
C	2.58520	5.47824	4.98027
C	1.87979	3.99032	3.21075
H	1.27866	4.93198	1.36390
H	2.53587	7.59290	4.52453
H	2.95831	5.62120	5.99508
H	2.54803	3.32377	5.15720
N	1.63815	2.68311	2.72683
N	1.53580	2.48821	1.32755
C	1.31554	1.54861	3.44450
O	1.20662	1.42149	4.65700
C	1.10224	0.44076	2.37025
C	1.20735	1.26141	1.09893
C	1.00538	0.71289	-0.26259
H	1.13507	1.49539	-1.01924
H	-0.00412	0.28304	-0.34721
H	1.71825	-0.10394	-0.45488
H	1.61967	-3.78835	2.25782
H	1.75029	-3.38956	4.07878
C	2.06330	-1.79917	2.75057
C	2.26467	-0.53761	2.46335
H	3.26878	-0.13098	2.31580

30

VI

G= -763.416010 ha

H	1.252085	0.696785	0.672169
C	0.744550	0.365786	1.580550
C	-0.554139	-0.507336	3.897664
C	0.754256	-0.989850	1.906889
C	0.089569	1.297632	2.394791
C	-0.554861	0.844901	3.551417
C	0.103052	-1.446124	3.073094
H	1.259191	-1.715531	1.273130
H	0.082574	2.356605	2.132131
H	-1.067487	1.555444	4.203201
H	-1.052476	-0.864980	4.795493
N	0.122775	-2.806560	3.391092

N	0.886815	-3.673136	2.608519
C	0.680872	-4.890569	3.138504
C	1.352483	-6.093571	2.559109
H	1.949591	-6.623935	3.317220
H	2.017667	-5.795190	1.737307
H	0.617799	-6.814861	2.167986
C	-0.207493	-4.840049	4.224672
C	-0.599243	-3.491187	4.419742
O	-1.381467	-2.957257	5.264781
O	-0.671072	-5.923450	4.959048
C	-0.219554	-5.905351	6.319846
H	0.888655	-5.904885	6.343930
H	-0.548690	-4.967898	6.807334
C	-0.742787	-7.082466	7.074101
H	-0.409570	-7.130372	8.116144
C	-1.550526	-8.028107	6.583521
H	-1.890167	-8.857678	7.205440
H	-1.895481	-7.997729	5.549384

30

TS1A

G= -763.325014 ha (+57.1 kcal/mol with respect to VI)

C	0.272458	0.417396	1.692227
C	-0.201316	0.572455	-0.189815
H	-4.940098	-0.488821	5.909026
C	-4.365894	0.293996	5.409637
C	-2.880252	2.278365	4.115979
C	-3.292609	-0.068088	4.596629
C	-4.712443	1.638862	5.587546
C	-3.959518	2.619505	4.933020
C	-2.533593	0.921803	3.936702
H	-3.024365	-1.112949	4.456471
H	-5.554624	1.916527	6.223096
H	-4.212893	3.674507	5.057898
H	-2.290846	3.037570	3.608287
N	-1.460836	0.545070	3.122810
N	-1.144069	-0.812865	3.012561
C	-0.074237	-0.864785	2.210836
C	0.561663	-2.169117	1.868656
H	0.388428	-2.436441	0.814075
H	0.160350	-2.969010	2.505326
H	1.653635	-2.118843	2.002356
C	-0.566491	1.366344	2.388174
O	-0.590004	2.628385	2.329452
O	1.367643	0.752960	0.887074
H	-1.230901	0.234587	0.025670
H	-0.204593	1.652459	-0.359463

C	0.337856	-0.246867	-1.298490
H	-0.433964	-0.454053	-2.054554
C	1.559352	-0.764524	-1.461575
H	1.794053	-1.363172	-2.343193
H	2.322069	-0.621217	-0.698077

28

VII

G= -763.413778 ha (+1.4 kcal/mol with respect to VI)

O	-0.090603	-0.000310	-0.886142
C	-0.065071	-0.106927	2.818847
H	4.881680	2.211466	-6.143787
C	4.141976	2.606528	-5.428938
C	2.252738	3.593440	-3.593871
C	3.683006	1.777963	-4.395018
C	3.671829	3.926083	-5.559529
C	2.727189	4.406260	-4.634228
C	2.731445	2.264749	-3.464256
H	4.042657	0.747189	-4.288055
H	4.036508	4.571933	-6.372881
H	2.344468	5.435356	-4.721301
H	1.520734	3.964682	-2.866655
N	2.282678	1.427886	-2.427673
N	2.658324	0.060091	-2.457381
C	1.461951	1.737173	-1.334294
O	0.988545	2.844845	-1.071572
C	1.169349	0.375469	-0.619040
C	2.128971	-0.521189	-1.422721
C	2.247329	-1.982161	-1.167955
H	2.711598	-2.518645	-2.018201
H	1.221354	-2.367464	-0.990798
H	2.835769	-2.178962	-0.246153
H	-0.159351	-1.133323	3.218196
H	-0.714753	0.664239	3.271940
C	0.785567	0.185261	1.852069
C	1.605414	0.468819	0.859516
H	2.644673	0.791019	1.065930

30

TS2A

G= -763.390193 ha (+16.2 kcal/mol with respect to VI)

C	0.129149	0.184074	2.407852
C	0.238502	0.121798	0.291152
H	5.200464	-3.904687	4.265310
C	4.998902	-2.900585	3.887354
C	4.457286	-0.333636	2.920581

C	3.679511	-2.519130	3.645984
C	6.056894	-2.013729	3.656982
C	5.770769	-0.733162	3.171494
C	3.392532	-1.227048	3.160540
H	2.856372	-3.207670	3.824829
H	7.086566	-2.316672	3.851710
H	6.582252	-0.028713	2.978139
H	4.232838	0.660646	2.543267
N	2.066077	-0.855252	2.913197
N	1.058547	-1.826670	3.009528
C	-0.076960	-1.194976	2.746384
C	-1.381393	-1.914044	2.748716
H	-2.089924	-1.440463	3.445112
H	-1.245791	-2.964727	3.036644
H	-1.848290	-1.880117	1.749717
C	1.542988	0.431217	2.627798
O	2.195455	1.496737	2.558233
O	-0.817619	1.158826	2.502329
C	-1.366726	1.624413	1.058005
H	-2.220779	0.951890	0.897653
H	-1.696910	2.655308	1.222534
C	-0.238441	1.416013	0.171068
H	0.483619	2.232999	0.073986
H	1.251729	-0.148333	-0.018323
H	-0.468522	-0.712355	0.282594

33

VI-Me

G= -802.724276 ha

H	-1.55898658801072	2.16433949789991	1.31695245640064
C	-0.80276008041154	1.37891812808605	1.38616758240646
C	1.11131474596592	-0.63988116077909	1.58196889090431
C	-0.93818419348374	0.39907614110209	2.37097312115010
C	0.27490815325077	1.36684370672043	0.49380522618116
C	1.22674740820723	0.34594577898979	0.60312866544823
C	0.02417247225473	-0.62779470315685	2.48198209283865
H	-1.77528443060813	0.40325087482434	3.06588779465718
H	0.37157211451655	2.13731222497982	-0.27291679264746
H	2.07718140233510	0.31620450264468	-0.08178030570320
H	1.85168528390628	-1.43256936997341	1.66877479727216
N	-0.08025285680931	-1.62540982987819	3.45380489697863
N	0.84940575941368	-2.66615243489344	3.45452768731411
C	0.50311614984119	-3.43861338686904	4.49768729412763
C	1.28066724496725	-4.67393221483797	4.81865708878678
H	2.06093173891339	-4.83590147750548	4.06275019586467
H	0.62943624254490	-5.56113099310289	4.84646860554112
H	1.76429679964647	-4.60066222170792	5.80552065753098

C	-1.01830834879561	-1.72912778444533	4.53005350209871
O	-1.96123242610106	-0.91629221822187	4.77845290514149
C	-0.60776430074351	-2.91978686152050	5.18205391010099
O	-1.18860754738744	-3.44950578669266	6.32588747432615
C	-2.51250659089876	-3.95613359959916	6.09311887934444
H	-2.47754177382587	-4.71113452536324	5.28186194846684
H	-3.16115962764980	-3.13447062893659	5.73371295085732
C	-3.08103145583651	-4.56240153668765	7.33487160672294
H	-4.08735511002210	-4.98015411129748	7.21436498676526
C	-2.47327696974845	-4.62710126050059	8.52537114183570
H	-1.46993933300884	-4.20202792160812	8.62154385938210
C	-3.06704799619592	-5.24619409545737	9.75551048833471
H	-3.14153275317523	-4.51164128174986	10.57470672610692
H	-2.43467987719201	-6.06730624845893	10.13171866464056
H	-4.07198325585883	-5.64756520200337	9.56241100082364

33

7

G= -802.719798 ha

H	-1.27666523785814	2.35545511524356	4.12848672492430
C	-0.77099225443080	1.47290901302424	3.73288099429206
C	0.50872751596271	-0.79957601042052	2.73845432981251
C	-0.99007004261126	0.23898878691977	4.34724212945657
C	0.07656735309722	1.58912389667524	2.62633654312114
C	0.71070220782361	0.44268890574351	2.13647350443022
C	-0.34609454545623	-0.91341352731943	3.85253977513899
H	-1.64737297494041	0.15183331703521	5.20809308484421
H	0.23952357863616	2.55775806855331	2.15247913468083
H	1.37372411314547	0.51131725900291	1.27197037577306
H	1.00223313112533	-1.69016873370708	2.35726875007277
N	-0.54073717840513	-2.16744963676782	4.45429642796723
N	0.01461425784626	-3.31474805821119	3.81476645660564
C	-0.29168010169611	-4.35251105459345	4.52712796039988
C	0.10516443797434	-5.71843225471769	4.09649227917097
H	0.48209865886735	-5.71748385751292	3.06516543597356
H	-0.76465919328977	-6.38571436464842	4.17733804848617
H	0.88499814187181	-6.12500244891172	4.75923996953202
C	-1.24237294694270	-2.50359104494830	5.60513053912960
O	-1.81974588550680	-1.71950434136690	6.36364633094052
C	-1.20578330864742	-4.05012603097328	5.70963383603868
O	-2.44511436800095	-4.56013973281528	5.48399570050839
C	-0.57628925271951	-4.40517971486040	7.11087191027229
H	0.39385563256460	-3.88974337306081	7.21407184554719
C	-0.42931163723201	-5.87132508484200	7.34710293655419
H	0.53117225101798	-6.32767665225910	7.08036431061097
C	-1.41120338255784	-6.67123568929260	7.78978007767653
H	-2.37494018978707	-6.21751102794920	8.04670590192788

H	-1.27301669505231	-3.95520787176149	7.83707147510030
C	-1.30140993132096	-8.15766388760961	7.94998504976632
H	-0.29541966464146	-8.51595807599489	7.68633385937746
H	-2.02835840785647	-8.68257868415939	7.30818480447278
H	-1.51914408097951	-8.47213320349424	8.98446949739477

33
8
G= -802.712573 ha

H	-0.72406279446374	2.29989604564422	4.45353031572029
C	-0.34843325105823	1.37763127939175	4.00744027044281
C	0.60611315090503	-0.99623123356846	2.89152702124084
C	-0.80692244194190	0.15499346781916	4.49929514272748
C	0.58178738211230	1.43180915504130	2.96450181860417
C	1.05555473512814	0.23583546026953	2.41601201796939
C	-0.33436498304798	-1.04704572309301	3.93738586386972
H	-1.52975569175515	0.11861786368002	5.30996753573357
H	0.93498808238419	2.39192462699388	2.58712493896020
H	1.78388685356617	0.25738128366450	1.60295444867769
H	0.97548891097604	-1.92624576761954	2.46611907648566
N	-0.77674727819222	-2.29097680328933	4.42189391945599
N	-0.14733109528134	-3.46730591762122	3.92365379312207
C	-0.73815688817683	-4.48698766327186	4.46119641155641
C	-0.42611754172268	-5.87120098538718	4.02331558964503
H	0.32080654825194	-5.87354527562649	3.21847094333624
H	-1.35812650040784	-6.33700264640535	3.66737833419964
H	-0.07181573009847	-6.48853399253685	4.86074849289980
C	-1.90367024033157	-2.58930797875797	5.18343244706793
O	-2.74010945563560	-1.77673548873788	5.57919766021273
C	-1.86114263220294	-4.12940831369527	5.42958624731436
O	-2.99810494351760	-4.79989009248850	5.30993455322621
C	-1.19360283945629	-4.17015220234404	6.97514765287425
H	-0.41576474462099	-3.38781894541362	7.00093540416267
C	-0.54139457011716	-5.48685620544170	7.22180972870897
H	0.48078327094918	-5.58914032764889	6.84189608350255
C	-1.10484441862366	-6.54852171881755	7.81977333323111
H	-0.56217239249259	-7.49006933589986	7.92669052359931
H	-2.11739445993107	-6.51403959721116	8.22441161346975
C	-2.31746058706627	-3.84516141659883	7.95161030213948
H	-1.97902000527422	-3.97006260416825	8.99103406020564
H	-3.16574455312257	-4.52000828026107	7.76953826366848
H	-2.67414889573403	-2.81684066660024	7.81448619196917

35
1h
G= -840.550118 ha

C	-0.36683938387973	-0.00250369041925	0.75585988440317
C	-0.77445316025040	0.87647403178022	-0.42232813458128
H	-0.56121014647901	1.93267056199507	-0.20910036884390
H	-1.85230770356959	0.76653939303530	-0.60225804664416
H	-0.23558147388464	0.58049051028862	-1.33209222396008
C	-1.07513814937638	0.42746021016888	2.03520782466126
H	-0.86460221896323	1.48255096069876	2.25742604603945
H	-0.77070407728497	-0.18714962279149	2.89044782875365
H	-2.15934297156445	0.31425259593140	1.89279344002907
C	-0.56692816826938	-1.47793952302286	0.42661806921481
H	0.08792126285748	-1.78307056704262	-0.40075695693909
H	-1.60913716078304	-1.62828877768499	0.11312162926759
H	-0.38313653674155	-2.12704152756616	1.28959905529300
O	1.10900858577124	0.31604941631441	0.86787325047497
C	1.97748795087023	-0.23277942058136	1.71186829639017
O	3.14451772940379	0.12318303329320	1.74099148730041
N	1.46311351345728	-1.14677279890399	2.68734876121515
N	1.97271927879493	-2.29969558839828	2.62676088516907
C	1.52207102697070	-3.17380728428113	3.64992270247893
C	2.09300442565249	-4.40068654780205	3.55804816384436
H	2.81717911636946	-4.55599241229598	2.75568749540846
C	0.54981992846597	-2.70102942273340	4.67587145150085
H	0.91693996043653	-1.77410048245212	5.13849680077318
H	0.37926409185324	-3.46329061969722	5.44090071182018
H	-0.41229258575170	-2.45663382017451	4.20105392866430
C	1.79119413367283	-5.54188341539655	4.45324498133393
O	0.65819510207365	-5.83761236288420	4.81099556461858
O	2.82410967645834	-6.31252757378173	4.85065536046366
C	4.20284652316046	-5.91960160795829	4.53039925027061
H	4.37042982805132	-6.09485856204771	3.45788744799895
H	4.32382842693646	-4.84869517084389	4.74733634521109
C	5.11995403893073	-6.76742009772694	5.38148893408735
H	4.97565002473203	-7.83622710448162	5.16995098166100
H	4.94282453397144	-6.58720804478547	6.45101534108290
H	6.16359457790745	-6.50785466775207	5.15366381153839

35

1h-int

G= -878.213701 ha

C	-1.15233942405636	-0.03564057800865	0.89005682441516
C	-1.61371527396110	0.96125793768426	1.95199564345513
H	-2.20731751377998	0.45475604005130	2.72571207859820
H	-2.23432053615127	1.73970655043322	1.48663028293910
H	-0.74924921375039	1.43944259592477	2.43140983176436
C	-2.35154128700430	-0.76223972397446	0.28056248394518
H	-2.89663753234270	-1.31381655456806	1.05998090158259
H	-2.03963562582081	-1.46290272262977	-0.50127453108656

H	-3.03605694003407	-0.02140282821145	-0.15720680078588
C	-0.28918048200389	0.65881842995979	-0.16312346364187
H	0.58122440369258	1.13480831417271	0.30956004332979
H	-0.88261626526581	1.44168601645189	-0.65723405177188
H	0.05871431193955	-0.05117901563876	-0.92093790604145
O	-0.32818230685474	-0.97768234327474	1.68498369059023
C	0.26314921886173	-2.03813652468722	1.09680044500252
O	0.18050647000530	-2.34516023825750	-0.08860848287410
N	1.58902148385768	-3.95470289592361	1.57726357623752
N	0.99067114133827	-2.76848323672791	2.02677780011690
C	2.19976719494935	-4.45180463177027	2.65974704648708
C	2.93544661424740	-5.74937686914153	2.58118094891491
H	3.99757139911959	-5.62609244281004	2.84339660378576
H	2.87060991187174	-6.16155268042758	1.56483746026290
H	2.51271878009263	-6.48145293893228	3.28635209273736
C	2.05621970440855	-3.62276567467921	3.78705679453188
O	2.59588870098221	-3.84491393996413	5.04539875880810
C	1.57450436169651	-4.09750175540110	6.06389665797885
H	0.83180190210394	-3.28443560262647	6.02953597897998
H	2.11792183148498	-4.05517382769220	7.01853817895001
C	0.92934666554764	-5.42918402319406	5.85150684040842
H	1.54756212804936	-6.31002429298094	6.05380182004580
C	-0.30657445572222	-5.57341031669747	5.36112187020871
H	-0.73954242753247	-6.55639121388551	5.16522510801790
C	1.26378396756173	-2.50514691607405	3.43529664521388
O	0.86033049506133	-1.51918644651469	4.10361840984160
H	-0.92733042148901	-4.70161205678409	5.13794470424331

35
9
G= -878.215454 ha (-1.1 kcal/mol with respect to 1h-int)

C	-0.96503340705233	0.22212735306732	0.66230890590788
C	-1.17333808465908	1.45337299946590	1.54054053507226
H	-1.82915618866398	1.21692113902868	2.38881516616132
H	-1.63880991962292	2.25249107391357	0.94770210043271
H	-0.21270990404407	1.81855673761351	1.92834018381601
C	-2.30235895384318	-0.32573436871007	0.16762495537725
H	-2.94978971956559	-0.58164327963106	1.01810539086704
H	-2.16314507341395	-1.21308992215157	-0.45942572521006
H	-2.80612771911871	0.45048172681010	-0.42651600697063
C	0.00818357042754	0.52499692623606	-0.47580426491041
H	0.98053636294673	0.83856797630328	-0.07163501508295
H	-0.39423607770873	1.35097402534344	-1.08010500042428
H	0.15292116390485	-0.34760216905906	-1.12211470283365
O	-0.34291051685123	-0.73968674634215	1.61717229289559
C	-0.00404330013379	-1.97443708302040	1.21965770782090
O	-0.11895215061393	-2.44990919799086	0.09910204051697

N	1.13381223714480	-3.94814644642458	1.99112165749499
N	0.53127997196060	-2.69359540152210	2.29657985061125
C	1.58471953518653	-4.44622421875467	3.09842366061815
C	2.32195769429652	-5.73697426922507	3.10581874197431
H	3.27141367818052	-5.60548487584856	3.64515104276282
H	2.52229692480589	-6.08595097772001	2.08465029310427
H	1.75068581212924	-6.50484507084466	3.64930748399674
C	1.43565333252567	-3.53267380381724	4.31043394276931
O	2.65427435236948	-3.01025946235519	4.64214677328387
C	0.63908395704777	-4.15875756730140	5.49537229237610
H	-0.32834765563586	-4.56573467933463	5.15519174084584
H	0.42367920233429	-3.30320933228739	6.16208077158069
C	1.42124488369043	-5.19066149643187	6.23726357581191
H	2.45225488708917	-4.90385841760069	6.46758731023900
C	0.96719326630084	-6.39395711128920	6.60971626339893
H	1.60107940757667	-7.10368081647059	7.14566651532806
H	-0.05457861109971	-6.71415527708730	6.38641840522793
C	0.58380906404726	-2.40236352561518	3.67866100663326
O	0.10137900749780	-1.42960578491619	4.24134609433150

38
1h-int-Me
G= -917.522311 ha

C	-1.20558420087816	-0.11428951110239	1.16873026474878
C	-1.41389632742532	1.03115720626652	2.15835442393398
H	-1.80853508943739	0.65015624032337	3.10951042818105
H	-2.12951801321267	1.75507097958623	1.74417062099926
H	-0.46462858809395	1.54752514135652	2.35610693353503
C	-2.51262847111640	-0.87452646353279	0.94330734297544
H	-2.88492261759254	-1.28161367730660	1.89394844437816
H	-2.37711044856006	-1.69570658275444	0.23119967620006
H	-3.26876324260357	-0.18157215531605	0.54734058419499
C	-0.59554430622356	0.40328132188475	-0.13430060813905
H	0.36705624196847	0.89499206089364	0.06539597531400
H	-1.27461986126476	1.14605007225546	-0.57793860101078
H	-0.44033072174524	-0.41055908334152	-0.85100437617496
O	-0.23322863515839	-0.97214439434039	1.88778387625387
C	0.18530900230251	-2.13586283057814	1.34962743661306
O	-0.10888696381933	-2.56384738768614	0.23759585988270
N	1.53316927354542	-4.04259170846641	1.80512920607392
N	1.01571064175415	-2.81101433417714	2.23364277026628
C	2.18646829513347	-4.52052259377228	2.87109354562345
C	2.87637361511450	-5.84301647086747	2.80063303505706
H	3.96242359433354	-5.73666332188681	2.94950055879977
H	2.70199121462212	-6.31195299847168	1.82224887504772
H	2.50622465588333	-6.51675169444944	3.58800344615288
C	2.13707914436345	-3.64306647108529	3.97035435465184

O	2.67169731140249	-3.86092903818805	5.23077576037220
C	1.62616971002766	-4.12913820551492	6.23194319097717
H	0.90991642729103	-3.29400656689956	6.21129616429593
H	2.15693919529829	-4.11908422376094	7.19346181451390
C	0.96941810805843	-5.44991011193448	5.98549805674889
H	1.50937982900824	-6.32950754829147	6.34933509819275
C	-0.15859345963066	-5.64502730299263	5.28037817555368
H	-0.47879558061189	-6.68225120081027	5.12624038732512
C	1.37262534150535	-2.50818532311784	3.61218667841847
O	1.02796963897380	-1.49105733037063	4.26763884173159
C	-1.03766806367035	-4.60947757593039	4.65123926134755
H	-0.99040390942212	-4.69562933848817	3.55237765388801
H	-0.74930252225400	-3.58422638845270	4.91136372591542
H	-2.09053727220879	-4.76780594073700	4.93584970060219

38

10

G= -917.522454 ha (-0.09 kcal/mo with respect to 1h-int-Me)

C	-0.91922742811442	0.34939504877902	0.60611919286130
C	-1.12439259857913	1.57011504420107	1.49973352556806
H	-1.80848455298985	1.33362411545863	2.32524866692904
H	-1.55509594423449	2.38994380782040	0.90888472236960
H	-0.16678629555582	1.90632694554115	1.92002622997963
C	-2.25395129427941	-0.15738669834567	0.06293492043732
H	-2.93197042341056	-0.41205586928920	0.88961663171736
H	-2.11718591496988	-1.03723541130597	-0.57517402050190
H	-2.72144457031064	0.64068501126882	-0.53192792371443
C	0.09322708981938	0.64829845157294	-0.49838817017488
H	1.05958757641825	0.93574422439361	-0.06143271331448
H	-0.27368010431609	1.49089594221344	-1.10240785746515
H	0.23880928190746	-0.21821982211678	-1.15273847229927
O	-0.34816891898247	-0.64268735037641	1.56053564504903
C	-0.02567072385624	-1.87854462729882	1.15175587713957
O	-0.11783683094108	-2.33211188778000	0.01992272670068
N	1.05641664337153	-3.88359597110674	1.92236921713781
N	0.46183192669901	-2.62621291389731	2.23132107251612
C	1.47678477736420	-4.40100284695315	3.03308472951107
C	2.19390992732148	-5.70291922205181	3.04022118621748
H	3.14462973159070	-5.58807040179456	3.58106060260640
H	2.38982617659315	-6.05388708778586	2.01882251820489
H	1.60993634239912	-6.46141689976507	3.58350096852194
C	1.30694441569674	-3.50337011462205	4.25344443247716
O	2.52034432561290	-2.98337492596425	4.61299722264196
C	0.48650023389233	-4.15039410070469	5.40977783213028
H	-0.46254381451341	-4.55688120791921	5.02562595617941
H	0.23260767472088	-3.30688994301535	6.07834944367641
C	1.27453203661301	-5.17356563201116	6.16043904253044

H	2.28954560843905	-4.85339383477646	6.41329625532667
C	0.88623529231823	-6.40729686445772	6.52207668600300
H	1.61196982498488	-7.02825888972592	7.05976179177959
C	0.47737802382251	-2.35965903755543	3.62009584716107
O	-0.01671234244450	-1.39384891260789	4.18534199277483
C	-0.44366709313307	-7.05400560953376	6.26431351449253
H	-0.32293579839429	-7.97968376837285	5.67725845313787
H	-1.13435283905801	-6.39574467190751	5.72103272757018
H	-0.92918377578036	-7.34819237934648	7.20939112453588

38

11

G= -917.511633 ha (+6.7 kcal/mol with respect to 1h-int-Me)

N	-0.52678884265303	-3.15487165412564	4.61200053455938
N	-0.77992316328174	-4.45098066392046	4.07779034987054
C	-1.56481866691999	-5.06732435115199	4.90286229065780
C	-2.08759507803759	-6.41937040201024	4.56069252930375
H	-2.03653955331315	-6.59763693464331	3.47791587200381
H	-3.12788326349265	-6.49309120442388	4.90527515540244
H	-1.51542301693398	-7.20826468998332	5.07045564240271
C	-1.28692090775701	-2.89912029186777	5.77607507624580
O	-1.34586142125491	-1.82993997341288	6.36357491232930
C	-2.05763914269041	-4.22458714569934	6.08252885995499
O	-3.39297472309581	-4.05408614559569	6.01312292981418
C	-1.49720746133162	-4.75279790162084	7.50223787748927
H	-0.40087569999266	-4.61417727199390	7.47407208609453
C	-1.76565360855565	-6.21664239955524	7.67421069444023
H	-1.05521817158845	-6.89082154452892	7.18481577813543
C	-2.80119590909955	-6.75784330586513	8.33127482576549
H	-2.93702255893682	-7.84038833590918	8.37972988766980
H	-3.54605154371568	-6.14413019858381	8.84081141473391
C	-2.09785614024822	-3.89538351867146	8.61657737609502
H	-1.79677486613530	-4.27827360771366	9.60274920538124
H	-3.19445712417977	-3.91404314169363	8.55050187037158
H	-1.77502076747948	-2.85162090943979	8.52482459294207
C	0.29818182315776	-2.25205916799902	3.93363065601962
O	0.51321982730260	-1.11897060221735	4.33920935782232
O	0.80055806374618	-2.82159593134931	2.82336918316582
C	1.72801571741207	-2.05902652386623	1.93705355467354
C	2.01851330687274	-3.07402880130508	0.83368678498579
H	2.48453708383213	-3.97659553109302	1.25147268638361
H	2.70598622020510	-2.63312563053206	0.09855974432956
H	1.09109383917973	-3.36058310930320	0.31916936908936
C	1.01810575514036	-0.83105406085944	1.36925199956215
H	0.81027902855639	-0.09141416303767	2.14991548430059
H	0.07403933433101	-1.12505515390065	0.88996663869665
H	1.66165946945180	-0.37027077251873	0.60622103198504

C	3.00201814999025	-1.70705301956615	2.70230204061606
H	2.80056758225508	-0.98558176379109	3.50125989398106
H	3.72899384154115	-1.26752410093559	2.00465446575000
H	3.44793258771914	-2.61266607531532	3.13717734697552

27
Diaza-diene
G= -686.465945 ha

H	-2.36632425298844	-2.37996672588868	0.69167541815417
C	-1.39586303665126	-2.23615616120938	1.17528309050709
H	-1.15576506403596	-3.08931902570141	1.82266315886574
H	-0.60430258861462	-2.11015277024779	0.42519014733750
O	-1.53680748208065	-1.03129211506029	1.96403879401558
C	-0.43253382726858	-0.66313247542350	2.67226984008549
O	0.61355323703220	-1.30312601323627	2.64583684480488
C	-0.72600802838565	0.56696852290313	3.41984053703130
H	-1.72899168581169	0.97567502219814	3.29234225991290
C	0.11983920408911	1.24131831542230	4.24819669774303
C	1.52987268858734	0.87950717015495	4.57833792127223
H	2.20311291663814	1.70109154844049	4.29261576267839
H	1.82314184062216	-0.03904946298690	4.06533587963057
H	1.63850967198310	0.75648522644563	5.66584517672753
N	-0.47875864802886	2.39148635306623	4.81086233582618
N	0.28648486043317	3.03438055129686	5.59667668428346
C	-0.28066853194765	4.19314618377672	6.16953222930452
C	-1.19444961058555	6.52076671870046	7.41730784086913
C	-1.57657284622888	4.67507632361661	5.89211606412586
C	0.54915291617360	4.88434770339038	7.07048916169336
C	0.09103710142107	6.04431321591925	7.69396501700468
C	-2.02383072382005	5.83368638513950	6.51603593990202
H	-2.20683183767948	4.13250534761913	5.18838363951227
H	1.54762010948318	4.49109983961319	7.26557033456842
H	0.73478377554298	6.57828076903576	8.39384469891840
H	-3.02405605213582	6.21287500787001	6.30299336479685
H	-1.55434410574286	7.43018454514543	7.89975116042829

6
MeOH
G= -115.756779 ha

H	-0.00014304528359	-0.00514982763827	0.00625498343941
C	0.00800358013180	0.01605500919686	1.10395359292386
H	1.05564744011367	0.03642040082710	1.44792953380821
H	-0.49609277285317	0.93678529370738	1.44275097746907
O	-0.67853208384112	-1.16459749109498	1.54762819736836
H	-0.66388311826759	-1.16351338499809	2.52048271499109

7
Propargylic alcolate
G= -191.429602 ha
O -0.00009383191219 -0.00175811918553 0.97724832827981
C -0.01169564375348 -0.02048716497663 -0.38241280320848
H 1.00937119205655 -0.01926073724030 -0.86944025690732
H -0.52008887815763 0.86546951670738 -0.86871594274148
C -0.70203608161721 -1.21317138880107 -0.96739999699288
H -1.78552915812690 -3.09767083089857 -1.69448044074575
C -1.28092759848913 -2.21412127560528 -1.35679888768391

9
Allylic alcolate
G= -192.655382 ha
O -0.57165317914026 0.24285991045188 0.80563000623509
C 0.03788272722894 -0.02157231301094 -0.39242548180408
H 1.15798637747880 -0.18933595253253 -0.31382115910902
H -0.05083924837858 0.80572142463001 -1.15952167436492
C -0.53323182630853 -1.26680780584943 -1.04133536414996
H -0.45360534828448 -2.17507039373643 -0.42799264431983
C -1.19102426106278 -1.31565804048199 -2.20980900773288
H -1.64213169659521 -2.23503972944122 -2.59263775524631
H -1.30438354493789 -0.41709710002937 -2.82608691950808

12
Methallylic alcolate
G= -231.965403 ha
O -0.02673067453175 0.00207207825840 1.30493886147065
C -1.16694904358875 -0.40953471176054 1.90272665519833
H -1.51487802658599 -1.44767208083821 1.58826765106370
H -2.08389142228488 0.21837211030711 1.65123793203812
C -1.17808170422865 -0.46959154925060 3.42441175107438
H -2.11634756379396 -0.81161029527050 3.88928582695002
C -0.13588953337755 -0.13779452927144 4.19880771100049
H 0.77064164386534 0.19498360339107 3.67783560773846
C -0.10698314245901 -0.17999851892379 5.69957099404976
H 0.09610975671020 0.81490237518692 6.13252022626659
H 0.69101622353190 -0.84464703899819 6.07350899649870
H -1.06293651325690 -0.53681144283022 6.11234778665080

7. References

(1) (a) O. A. Attanasi, P. Filippone, A. Mei, S. Santeusanio, *Synthesis* 1984, 671. (b) O. A. Attanasi, P. Filippone, A. Mei, S. Santeusanio, *Synthesis* 1984, 873. (c) L. Preti, O. A. Attanasi, E. Caselli, G. Favi, C. Ori, P. Davoli, F. Felluga, F. Prati, *Eur. J. Org. Chem.* 2010, 4312.
